# Bayesian Hierarchical Modeling Approaches for Combining Information From Multiple Data Sources to Produce Annual Estimates of National Immunization Coverage

**DOI:** 10.1002/bimj.70189

**Published:** 2026-10-06

**Authors:** C. Edson Utazi, Warren C. Jochem, M. Carolina Danovaro‐Holliday, Padraic Murphy, Sujit K. Sahu, Marta Gacic‐Dobo, Andrew J. Tatem

**Affiliations:** ^1^ WorldPop, School of Geography and Environmental Science University of Southampton Southampton UK; ^2^ World Health Organization Geneva Switzerland; ^3^ United Nations Children's Fund New York USA; ^4^ Mathematical Sciences University of Southampton Southampton UK

**Keywords:** Bayesian inference, household survey data, multiple likelihood model, official data, reported administrative data, single likelihood model

## Abstract

Estimates of national immunization coverage (ENIC) are crucial for guiding policy and decision‐making in national immunization programs and setting the global immunization agenda. World Health Organization (WHO) and UNICEF estimates of national immunization coverage (WUENIC) are produced annually for various vaccine–dose combinations and all WHO Member States using information from multiple data sources and a deterministic computational logic approach. This approach, however, is incapable of characterizing the uncertainties inherent in coverage measurement and estimation. It also provides no statistically principled way of exploiting and accounting for the interdependence in immunization coverage data collected for multiple vaccines, countries, and time points. Here, we develop Bayesian hierarchical modeling approaches for producing accurate ENIC and associated uncertainties. We propose and explore two candidate models: a base single likelihood (BSL) model and an irregular data multiple likelihood (IDML) model, both of which differ in their handling of missing data and characterization of the uncertainties associated with the multiple input data sources. We provide a simulation study that demonstrates a high degree of accuracy of the estimates produced by the proposed models, and which also shows that the IDML model generally outperformed the BSL model in most cases we considered. We apply the methodology to produce coverage estimates for select vaccine–dose combinations for the period 2000–2019. A contributed R package, imcover, implementing the No‐U‐Turn Sampler in the Stan programming language enhances the utility and reproducibility of the methodology.

**Abbreviations**
Admin: Reported administrative coverage data
AFG: Afghanistan
AFR: WHO African region
AMR: WHO Region of the Americas
AR(1): First‐order autoregressive model
BSL: Base Single Likelihood
BHM: Bayesian Hierarchical Modeling
DJI: Djibouti
DTP1: Diphtheria–Tetanus–Pertussis‐containing Vaccine dose 1
DTP3: Diphtheria‐Tetanus‐Pertussis‐containing Vaccine dose 3
EMR: WHO Eastern Mediterranean Region
ENIC: Estimates of National Immunization Coverage
EPI: Expanded Program on Immunization
EUR: WHO European Region
IDML: Irregular Data Multiple Likelihood
JRF: Joint Reporting Form
MCMC: Markov Chain Monte Carlo
MCV1: Measles‐containing Vaccine dose 1
NUTS: No‐U‐Turn Sampler
Official: Country‐reported official coverage estimates
PCV3: Pneumococcal Conjugate Vaccine dose 3
SEAR: WHO South East Asian Region
Survey: Household surveys of vaccination coverage
UN: United Nations
UNICEF: United Nations International Children's Emergency Fund (UNICEF)
WHO: World Health Organization
WPR: WHO Western Pacific Region
WUENIC: WHO and UNICEF Estimates of National Immunization Coverage
yovi: year of vaccine introduction

## 1 Introduction

Accurate estimates of immunization coverage are required at the global, regional, national, and subnational levels to inform policies and guide interventions aimed at improving coverage and accelerating progress toward disease elimination and eradication (Burton et al. 2009; Gavi, The Vaccine Alliance 2020; World Health Organization 2020). In particular, globally comparable estimates of national immunization coverage (ENIC) are crucial for profiling countries and understanding where attention should be focused to strengthen global immunization service delivery. Immunization coverage is also an important indicator for measuring and monitoring progress toward the goals and targets set out in global policy frameworks such as the Sustainable Development Goals (United Nations General Assembly 2015) and the Immunization Agenda 2030 (IA2030) (World Health Organization 2020). Since 1998, the World Health Organization (WHO) and the United Nations International Children's Emergency Fund (UNICEF) have jointly published ENIC annually for 195 countries and territories and different vaccine–dose combinations (Burton et al. 2012;;2009; Danovaro‐Holliday et al. 2021).

WHO and UNICEF estimates of national immunization coverage (referred to as WUENIC) are produced through integrating information from multiple data sources using a computational logic approach (Burton et al. 2012;;2009). Fundamentally, the approach uses deterministic ad hoc estimation rules to supplement administrative and official ENIC reports to WHO and UNICEF (World Health Organization and United Nations Children's Fund 2022) with estimates obtained via nationally representative household surveys. The approach is implemented on a country‐by‐country basis, enabling the incorporation of expert knowledge and country‐specific adjustments (e.g., vaccine stockouts, conflict, and other data quality assessments). Although the approach encourages reproducibility, representing an improvement over informal, manual estimation procedures, it is incapable of characterizing the uncertainties inherent in the multiple input data sets and those associated with the output coverage estimates. Also, the approach provides no statistically principled way of accounting for and exploiting the different sources of variation or dependence that may exist in immunization coverage data collected for multiple vaccines, countries, and years. The entire time series produced by the approach is usually updated when new data become available; however, the approach is also unsuited for producing estimates of immunization coverage for future time points, which may be useful to guide program planning. To address these challenges, WHO and UNICEF made a call in 2019 for alternative approaches for producing ENIC (World Health Organization 2019a, 2019b).

Bayesian hierarchical modeling (BHM) approaches (Cressie and Wikle 2011; Sahu 2022) offer a robust, flexible framework to combine information from multiple data sources to estimate a phenomenon of interest, while accounting for the full range of uncertainty present in these data sources. BHM approaches have been widely applied in different thematic areas ranging from environmental studies to health and medicine (see, e.g., Sahu 2022). Godwin and Wakefield (2021) used a BHM to combine information from household surveys and censuses to produce smoothed estimates of child mortality after explicitly modeling (and removing) the biases associated with each data source. Their model formulations included single likelihoods and modeling the source‐specific biases using both fixed and independent and identically distributed (iid) random effects, with a given data source identified as the reference data in their fixed effect specification. Also, Manzi et al. (2011) used a BHM with a single likelihood to combine prevalence estimates from seven different studies to estimate smoking prevalence in 48 local areas of England. The bias associated with each data source was modeled using a random effect assumed to follow a normal distribution with a non‐zero source‐specific mean and variance. Wang et al. (2012) utilized a multiple likelihood BHM approach to estimate crop yield using data from three monthly surveys with different temporal supports, and one of the surveys was considered to be the “gold standard.” Data from the gold standard data source and those from the other two data sources were first modeled using two separate likelihoods, with the mean parameters from the models linked at a later stage in a latent process model to model the true crop yield. A detailed review of studies utilizing information from multiple data sources to estimate a quantity of interest in a BHM is provided in Lohr and Raghunathan (2017). In general, these methods are context‐specific and tailored to the intended applications.

In immunization coverage estimation, the use of BHM approaches has mostly focused on producing estimates of coverage at a high resolution (e.g., 1 or 5 km grid squares covering an area of interest) and for subnational administrative areas (e.g., the district level), where the intermediate levels of the model relate to characterizing the spatial and spatiotemporal variation in the data using geostatistical and conditional autoregressive models (Local Burden of Disease Vaccine Coverage Collaborators 2021; Utazi et al. 2021, 2020). Similar applications also exist in the estimation of other health and development indicators (e.g., Burstein et al. 2019; Giorgi et al. 2021; Sahu et al. 2006). While estimates of immunization coverage produced using these approaches are extremely valuable for uncovering the spatial heterogeneities in coverage that often exist within countries, ENIC and other indicators still remain the benchmark for policy and decision‐making, resource allocation, and monitoring and evaluating progress at the global level.

Here, we develop novel BHM approaches for producing ENIC as an alternative or complement to the WUENIC computational logic approach. Similar to the WUENIC approach, our methodology utilizes multiple input data and enables the incorporation of expert opinions and judgments, which can be implemented before or during model‐fitting. However, it also crucially provides a mechanism to leverage various sources of dependence in the input data to improve the estimation of coverage and associated uncertainties. We propose and explore two candidate models, namely a base single likelihood (BSL) model and an irregular data multiple likelihood (IDML) model, both of which differ in their handling of missing data and characterization of the uncertainties arising from the multiple input data sources. The proposed models are implemented for each of the six WHO regions but can also be implemented at the country level, although regional models provide a richer framework to exploit inter‐country variation. The methodology is supported by the development and GitHub publication of a contributed R package imcover to enhance its utility and to encourage reproducibility.

In what follows, we present and explore the data used in this work. We also outline the steps taken to process the data, including a recall‐bias adjustment for survey data as in the WUENIC approach (Brown et al. 2015; Burton et al. 2012, 2009). We then proceed to present the proposed methodology and its implementation in a Bayesian framework using the Stan package in R (Stan Development Team 2020). A simulation study assessing both the in‐sample and out‐of‐sample predictive performance of the methodology is presented in Section 5. Modeled estimates of coverage and associated uncertainties are presented and discussed in Section 6, as well as comparisons with corresponding WUENIC estimates (2020 revision published in 2021). In Section 7, a description of the accompanying imcover software package is provided. We conclude with a discussion on both the methodology and the modeled outputs and outline directions for future work.

## 2 Data Description and Processing

We assembled publicly available, aggregate data on national immunization coverage from the WHO immunization data portal (https://immunizationdata.who.int ; accessed on March 2, 2022). The data portal provides access to information collected through the WHO/UNICEF Joint Reporting Form (JRF) for Immunization Data (World Health Organization and United Nations Children's Fund 2022). We collected data from three main sources of information on national‐level immunization coverage:

i. reported administrative coverage data (admin);
ii. country‐reported official coverage estimates (official);
iii. household surveys of vaccination coverage (survey).

Administrative coverage data are estimates of immunization coverage that countries report annually to WHO and UNICEF through the JRF. Official estimates are coverage data which have been independently reviewed by countries against other in‐country datasets and which represent their assessment of the most likely coverage. In some cases, official estimates are the same as administrative estimates, but in other cases, they are based on surveys or “corrections” for known inaccuracies in the admin estimates (e.g., incomplete reporting) or other information (e.g., data quality assessments). Survey estimates are obtained from reports of nationally representative household surveys implemented in WHO Member States. There are four main survey data sources, which are also used for producing WUENIC: (i) the Expanded Program on Immunization cluster surveys (World Health Organization 2018) and surveys using previous WHO recommendations for vaccination coverage surveys (Danovaro‐Holliday et al. 2018); (ii) the Demographic and Health Surveys (ICF International 2022); (iii) the Multiple Indicator Cluster Surveys (United Nations Children's Fund 2022); and (iv) other country surveys. All three immunization coverage data sources (i.e., admin, official, and survey) are subject to different biases (Cutts et al. 2016, 2013; Scobie et al. 2020); for example, admin estimates can be made inaccurate by incomplete reporting, and survey estimates may not be representative for certain populations such as those living in conflict areas. These biases are listed in Supplementary Table S1. In practice, however, survey estimates are often considered more reliable but are produced more infrequently (Burton et al. 2012; Cutts et al. 2016; Galles et al. 2021; Lim et al. 2008). This expert view is recognized in our modeling approach in Section 3, but given the biases associated with all three data sources, we do not consider any of these as the gold standard or reference data source, which is in line with the consensus among immunization monitoring experts (Danovaro‐Holliday et al. 2021; World Health Organization 2019b).

From each of these immunization coverage data sources, we obtained annual coverage data for the years 2000 to 2019 for five vaccines, namely diphtheria–tetanus–pertussis‐containing vaccine doses 1 and 3 (DTP1 and DTP3), measles‐containing vaccine doses 1 and 2 (MCV1 and MCV2), and pneumococcal conjugate vaccine dose 3 (PCV3). We used the year 2019 as the latest year, as the performance of childhood immunization programs was affected by the COVID‐19 pandemic in most countries. We also obtained mid‐year estimates of each country's population sizes for the study period from the UN Population Division 2019 revision (United Nations, Department of Economic and Social Affairs, Population Division 2019). These population data were used as weights when producing regional estimates of immunization coverage from the national estimates, as explained in Section 4.2. The population data obtained varied for different vaccines, as these correspond to the target populations for these vaccines. In the case of DTP1, DTP3, MCV1, and PCV3, these were estimates of numbers of surviving infants (i.e., under 1 year old, even when MCV1 is recommended in the second year of life in some countries), while for MCV2, the population data was based on the target age provided in the national immunization schedule (World Health Organization 2022). Finally, we obtained information on the year of vaccine introduction (yovi) from the WHO repository. Since MCV2 and PCV3 vaccines were not administered throughout the study period in all the countries, we restricted our analyses to the periods when both vaccines were fully rolled out in each country.

All WHO Member States spread across six WHO regions (namely, African Region (AFR), Region of the Americas (AMR), Eastern Mediterranean Region (EMR), European Region (EUR), South‐East Asian Region (SEAR), and Western Pacific Region (WPR)) and for which data were available were included in this work (see Supplementary Figure S2). After obtaining the raw datasets described above, a multi‐step data cleaning and harmonization process was implemented to create analysis‐ready data for model fitting, as illustrated in Supplementary Figure S3. In the first stage of processing, standard vaccine‐source‐specific input data files were created for the study period from the raw data by extracting the relevant data required for the analysis. This was followed by a recall‐bias adjustment step implemented for DTP3 and PCV3 using survey data, to account for maternal recall biases when no documented evidence of vaccination was available to surveyors (Brown et al. 2015). This is detailed in the supplementary file. Further, to ensure that modeled estimates of DTP1 and DTP3 were consistent, i.e., that DTP1 is greater than or equal to DTP3 for all country–vaccine–time combinations, we opted to model DTP1 and the ratio of DTP1 and DTP3, i.e., DTP3/DTP1 (later on in the modeling step, we converted these ratios to DTP3 estimates by multiplying corresponding DTP1 and DTP3/DTP1 estimates). To calculate the ratios, we first preserved the differential between DTP1 and DTP3 where estimates of the former were greater than 100 by calculating the ratios using the original input data. We then rounded down those estimates greater than 100% to 99.9% (this is necessary for the logit‐transformation step in Section 3; admin estimates can sometimes exceed 100% due to the reasons outlined in Supplementary Table S1) and adjusted corresponding DTP3 estimates using the ratios obtained previously. Finally, for each country, vaccine, time, and data source, we scaled the coverage data to the unit interval and logit‐transformed these as a final pre‐modeling step. During this step, estimates of other vaccines greater than 1 were set to 0.999 and, to avoid undefined values in the transformation, which may happen when a country has nominally a vaccine in their immunization schedule but the vaccine becomes unavailable for any given reason, any estimates equal to zero were adjusted to 0.001. The outcome of these processing steps is a collection of country‐specific immunization coverage estimates from three major data sources. The three sources of information (admin, official, and survey) form three time series, although with different levels of variation over time and observation time points and completeness. The challenge of the proposed modeling approach outlined in Section 3 is to draw information from these different sources to estimate a true, but unobserved, national immunization coverage estimate.

Summaries of the processed data are presented in Table 1 at the global level and Figure 1 for each WHO region. For illustrations of the patterns of missingness in these data, see Supplementary Figures S2 and S4. In all, about 46% of the data were from administrative sources, while 47% and 7% were from official sources and surveys, respectively. 186 countries had administrative data, 194 countries had official data, while 131 had survey data for at least one of the five vaccines at any time point during the study period. When considering data from all sources at the global level, Table 1 shows that PCV3 had the smallest number of data points, the most variability and the lowest coverage due to being only universally recommended by WHO in 2009. DTP1 had the highest coverage and lowest variation, whereas DTP3 and MCV1 had very similar distributions.

**TABLE 1:** Summary of processed national immunization coverage data for the period 2000–2019 for all WHO Member States and data sources.

Vaccine/	No. of	Summary statistics (%)						
data	countries/	Min.	Q1	Med.	Mean	Std.	Q3	Max.
source	data points					dev.		
DTP1	189 (6787)	10.00	89.00	96.00	91.43	11.57	99.00	99.99
DTP3	189 (6763)	0.92	81.00	91.00	85.75	15.16	96.00	99.98
MCV1	194 (7577)	2.10	81.00	92.00	86.47	14.88	97.00	99.99
MCV2	176 (4191)	1.00	78.00	91.52	84.03	18.70	96.97	99.99
PCV3	148 (2144)	1.00	76.00	89.00	79.97	23.26	95.00	99.99
Admin	186 (12567)	0.92	83.36	93.00	87.21	15.79	97.40	99.99
Official	194 (12872)	1.00	84.00	93.00	87.44	15.27	97.20	99.99
Survey	131 (2023)	2.10	67.95	83.90	78.03	19.35	93.00	99.90
All vaccines/sources	194 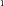 (27462)	0.92	82.20	92.53	86.64	16.02	97.00	99.99

Shown in the second column are numbers of countries with input data and numbers of non‐missing data points. (The number of Member States was 191 in 2000 but increased with the establishment of new countries.)

**FIGURE 1:**
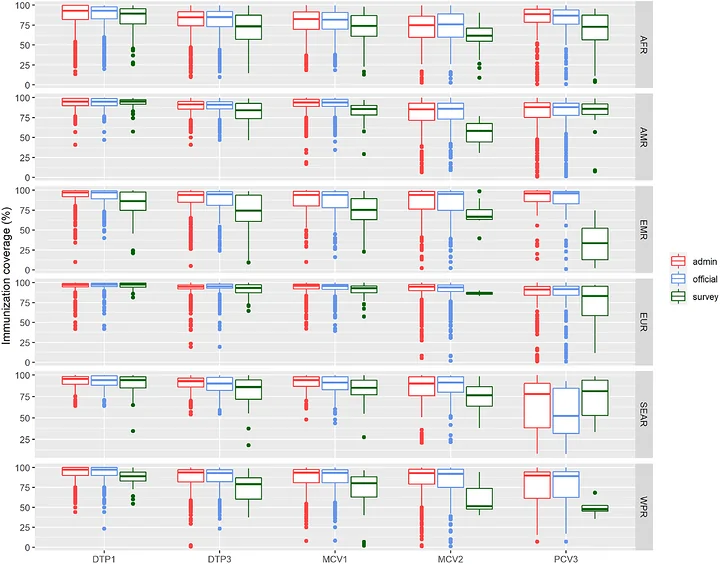
Distribution of processed national immunization coverage data (all vaccine–doses included) by WHO region and data source.

**FIGURE 2:**
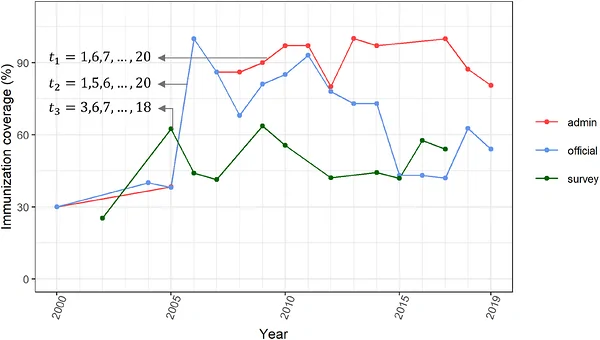
Data illustration for the irregular data multiple likelihood model using estimates of MCV1 coverage for Nigeria.

Further, Figure 1 shows that within each region, coverage data obtained from surveys were more likely to be lower on average than data obtained from official and administrative sources (except in the case of PCV3 in SEAR). We also observe that administrative and official data have very similar distributions as expected. We note that the processing steps outlined here have been developed in conjunction with the WHO/UNICEF immunization coverage working group. However, these can be improved upon to allow, for example, the exclusion of coverage data deemed implausible for estimation based on expert evaluation (unlikely zero estimates of coverage are excluded from the current analysis, although such estimates could sometimes reflect a vaccine stock‐out, as noted previously). The methodology proposed in Section 3 can produce coverage estimates for all desired cases using available input data, although with greater uncertainty where input data are missing, hence offering the flexibility to use the most accurate input data for coverage estimation.

## 3 The Proposed Methodology

Our aim is to develop BHM approaches for estimating national immunization coverage and associated uncertainties using multiple data sources. We propose two candidate models termed the BSL model and the IDML model. The BSL model assumes that observations are made regularly in time and uses a single likelihood to capture the variability in the data, implicitly treating unobserved data included in the likelihood as unknown quantities which must be estimated when the model is fitted. It is considered here as a suitable alternative against which to compare the IDML model, which acknowledges that observations are available at irregular time points and restricts the likelihood strictly to observed data points. As the name implies, the IDML model specifies a separate probability distribution for each data source to more flexibly estimate the variability in the data.

In general, let ${\tilde{p}}_{ijt}^{\left(k\right)}$ denote the kth type estimate of vaccination coverage (proportion) for the ith country i(i=1,⋯,C), jth vaccine j(j=1,…,V) and year t(t=1,…,T), where for

i. k=a, ${\tilde{p}}_{ijt}^{\left(a\right)}$ is the administrative estimate,
ii. k=o, ${\tilde{p}}_{ijt}^{\left(o\right)}$ is the official estimate,
iii. k=s, ${\tilde{p}}_{ijt}^{\left(s\right)}$ is the survey estimate.

Figure 2 provides a plot of these estimates in percentage forms.

The three versions of the estimates ${\tilde{p}}_{ijt}^{\left(k\right)}$ are available to us, but the numerator (number vaccinated) and denominator (target population or sample size) estimates corresponding to these estimated proportions are not always available for modeling. Also, for survey estimates, corresponding uncertainties are unavailable for our analysis. Hence, we are not able to use the binomial distribution or a weighted likelihood for modeling the true vaccination coverage $p_{ijt}$. Instead, we treat the logit‐transformed estimates,

$$y_{ijt}^{\left(k\right)}\equiv \text{logit}\left({\tilde{p}}_{ijt}^{\left(k\right)}\right)=\log\left(\frac{{\tilde{p}}_{ijt}^{\left(k\right)}}{1-{\tilde{p}}_{ijt}^{\left(k\right)}}\right)$$

as observed data varying over the real line. Note that the logit transformation used here is natural for transforming proportions to the real line. As is well known, possible alternatives to the logit transformation are the inverse cumulative distribution function (cdf) transformation such as the probit, i.e., ${\tilde{p}}_{ijt}=\Phi \left(y_{ijt}\right)$, where Φ(·) is the cdf of the standard normal distribution. In this work, however, we only adopt the logit transformation since it is able to accommodate more extreme values than the probit transformation. Hence, $0\leq {\tilde{p}}_{ijt}\leq 1$ for all i, j, and t.

The transformed estimates are then assumed to follow the normal distribution‐based linear models. Indeed, see Supplementary Figure S6 where the histograms of the logit and probit‐transformed estimates (panels (b) and (c)) show better bell‐shaped curves compared to the histogram of the proportions (panel (a)), which is negatively skewed as expected. We also observe deviant peaks in the right tails of the histograms in panels (b) and (c), which is due to the high frequency of proportions close to 1 in the data.

Before we introduce the models, we note that although we model on the logit‐transformed scale, we obtain and report the model‐based predictions on the original scale of 0% to 100%. Sampling‐based Bayesian computation methods also allow us to obtain the uncertainties of the predictions on the original scale. Details to obtain these predictions are provided in Section 4.2.

### 3.1 Base Single Likelihood (BSL) Model

We assume that all three estimates $y_{ijt}^{\left(a\right)}$, $y_{ijt,}^{\left(o\right)}$ and $y_{ijt}^{\left(s\right)}$ aim to estimate the true mean $\mu_{ijt}$, but each has its own bias. In this model, these biases are captured using a source‐specific random effect, $\nu^{\left(k\right)}$. The BSL model represents the observed $y_{ijt}^{\left(k\right)}$ as

$$y_{ijt}^{\left(k\right)}=\mu_{ijt}+\nu^{\left(k\right)}+e_{ijt}^{\left(k\right)}$$

where

(1) $$\mu_{ijt}=\lambda +\beta_{i}+\alpha_{j}+\gamma_{t}+\phi_{it}+\delta_{jt}+\psi_{ij}+\omega_{ijt}$$

is the true bias‐corrected mean, $e_{ijt}^{\left(k\right)}$ is an error term assumed to follow $N(0,\sigma^{2})$ independently and identically for all values of i,j and t, and λ is the overall mean. The source‐specific term, $\nu^{\left(k\right)}$, is modeled as 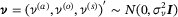, thus capturing the bias of coverage estimates from data source k relative to λ. Consequently, conditional on the shared mean $\mu_{ijt}$, the observations $y_{ijt}^{\left(k\right)}$ – and hence the data sources – are assumed to be independent in the model. In Equation (1), the terms $\beta_{i}$, $\alpha_{j}$, and $\gamma_{t}$ are the country‐level effect, the vaccine effect and the temporal effect, each of which captures overall variation in the data emanating from the respective attributes. Additionally, $\phi_{it},\;\delta_{jt}$, and $\psi_{ij}$ are country–time, vaccine–time, and country–vaccine interaction terms modeling trends that are specific to each country $\left(\phi_{it}\right)$ and vaccine $\left(\delta_{jt}\right)$, and random variation between each country and vaccine $\left(\psi_{ij}\right)$. Also, $\omega_{ijt}$ is a country‐vaccine‐time interaction that captures trends specific to each country‐vaccine combination. The detailed specification of these random effects is presented in Section 3.3.

We note that the source‐specific random effect, $\nu^{\left(k\right)}$, is not included in the shared mean $\mu_{ijt}$ in (1) as its role is to estimate the biases of the data sources relative to $\mu_{ijt}$, making its inclusion in the mean undesirable. However, model (1) offers limited flexibility to penalize the contribution of each data source to the shared mean. This is because such penalization is only achieved via the parameters $\nu^{\left(a\right)}$, $\nu^{\left(o\right)}$, and $\nu^{\left(s\right)}$, which – for identifiability reasons – are specified to be the same for all i,j and t. Furthermore, the error term $e_{ijt}^{\left(k\right)}$ is modeled as iid irrespective of k. We discuss this further in Section 3.2.

### 3.2 Irregular Data Multiple Likelihood (IDML) Model

The BSL model, as given in (1), is defined for all possible combinations of the indices i, j, k, and t. Thus, in principle, we need 3CVT data values to fit the model, where the factor 3 comes from three possible values of k, viz. admin, official and survey. However, for MCV1, for example, we only have 65.1% of these data values. Hence, the remaining 34.9% must be treated as missing in our Bayesian modeling.

The observed time points, denoted by the index t in ${\tilde{p}}_{ijt}^{\left(k\right)}$, as seen in the horizontal axis in Figure 2, are very much misaligned for the three types of estimates. In this figure, the survey and official estimates have been produced only for a few selected years and not for all the years. This presents a difficult problem in modeling based on balanced regular time series as for the BSL model in Section 3.1, since all the data, $y_{ijt}^{\left(k\right)}$, are not available for all regularly spaced values of t from 2000 to 2019. The problem is caused by the presence of a large percentage of missing data. Indeed, if we were to use regular time series models, then we would have 34.9% missing data for MCV1 from the three data series, $y_{ijt}^{\left(k\right)}$, k=a,o, and s. A suitable multiple imputation scheme – prior to or during model fitting – will be necessary to properly handle this large percentage of missing data.

Our proposed novel solution to this missing data problem comes through a multiple likelihood approach based on three different indices: $t_{1}$, $t_{2}$, and $t_{3}$ for admin, official and survey data, respectively, as illustrated in Figure 2. This essentially allows us to account for the irregular nature of the observations in the data, hence the term “irregular data multiple likelihood model.” Thus, we model $y_{ijt_{1}}^{\left(a\right)}$, $y_{ijt_{2}}^{\left(o\right)}$, $y_{ijt_{3}}^{\left(s\right)}$ where the time indices $t_{1}$, $t_{2}$, and $t_{3}$ depend on the country i and vaccine type j combination. For example, in Figure 2, $t_{1}$ (for admin estimates) takes the values 1, 6, 7, …, implying that the administrative estimates are missing for time points 2, 3, 4, and 5, and so on. In our model development, we simply write down the likelihood contributions based on the data from the observed time points. The combined likelihood function then captures all the information contained in the observed data for the underlying model parameters.

The IDML model is given by

$$\begin{array}{cc} y_{ijt_{1}}^{\left(a\right)} & =\lambda^{\left(a\right)}+\mu_{ijt_{1}}+\varepsilon_{ijt_{1}},\;t_{1}=1,\cdots ,T_{1}\\ y_{ijt_{2}}^{\left(o\right)} & =\lambda^{\left(o\right)}+\mu_{ijt_{2}}+\varepsilon_{ijt_{2}},\;t_{2}=1,\cdots ,T_{2}\\ y_{ijt_{3}}^{\left(s\right)} & =\lambda^{\left(s\right)}+\mu_{ijt_{3}}+\varepsilon_{ijt_{3}},\;t_{3}=1,\cdots ,T_{3} \end{array}$$

where

(2) $$\varepsilon_{ijt_{1}}∼N\left(0,\sigma_{1}^{2}\right),\;\varepsilon_{ijt_{2}}∼N\left(0,\sigma_{2}^{2}\right),\;\varepsilon_{ijt_{3}}∼N\left(0,\sigma_{3}^{2}\right)$$

and $\sigma_{k}^{2}$ for k=1,2 and 3 are source‐specific error variance parameters. Also, $\lambda^{\left(a\right)},\;\lambda^{\left(o\right)},$ and $\lambda^{\left(s\right)}$ are source‐specific intercept terms capturing the bias of coverage estimates from data source k relative to the true, bias‐corrected mean, $\mu_{ijt}$. The time indices $t_{1},t_{2}$ and $t_{3}$ are possibly unequally spaced, denoting only the time points for which data are available from a given data source. Similarly, $T_{1},T_{2}$ and $T_{3}$ are the total numbers of times data are available from the respective data sources. We note that if, for example, there are no survey data for country i and vaccine j, then $T_{3}=0$.

The shared mean for model (2) is given by

(3) $$\mu_{ijt}=\beta_{i}+\alpha_{j}+\gamma_{t}+\phi_{it}+\delta_{jt}+\psi_{ij}+\omega_{ijt},\;t=1,\cdots ,T$$

Hence, the IDML model also brings out the novel feature that, to estimate $\mu_{ijt}$, we are directly able to combine information from all the available sources for that specific i (country), j (vaccine), and t (time), with appropriate relative weighting as estimated by the variance components $\sigma_{1}^{2}$, $\sigma_{2}^{2}$ and $\sigma_{3}^{2}$. Another likely advantage is that unlike the BSL model, the IDML model does not need to estimate the input data for the missing cases, e.g., the admin estimate $y_{ijt}^{\left(a\right)}$ for MCV1 in Nigeria in 2006. In a Bayesian framework, these missing values in the likelihood are treated as parameters which are estimated when the model is fitted. Thus, the IDML model yields a lower dimensional parameter space, which is advantageous in Markov chain Monte Carlo (MCMC)‐based Bayesian computation, and consequently, reduced posterior uncertainty.

We note that the shared mean in (3), which enforces conditional independence between the data sources as in (1), does not include the source‐specific intercept terms $\lambda^{\left(a\right)}$, $\lambda^{\left(o\right)}$, and $\lambda^{\left(s\right)}$. This is because these terms play a similar role as $\nu^{\left(k\right)}$ in the BSL model in accounting for the biases arising from the various data sources and are also not desirable in the true, bias‐corrected mean. However, unlike $\nu^{\left(k\right)}$, which is modeled as a random effect, these terms are modeled as fixed effects. Also, we note that unlike (1), which includes an overall intercept term λ, the shared mean for the IDML model in (3) does not include an intercept term, which is a direct consequence of the different approaches adopted in accounting for the biases arising from the data sources in both models.

In model (2), the data sources are weighted by their respective variance parameters – $\sigma_{1}^{2},\sigma_{2}^{2},$ and $\sigma_{3}^{2}$, which in turn determine their contributions to the overall mean function in Equation (3). The influence of each data source in the model can thus be controlled by adjusting the values of these parameters either directly or through the prior distributions placed on them. For example, to increase the influence of survey estimates in the model, an informative prior that encourages smaller values relative to $\sigma_{1}^{2}$ and $\sigma_{2}^{2}$ could be placed on $\sigma_{3}^{2}$. In contrast, the BSL specification does not provide a mechanism for this adjustment since it assumes that $\sigma_{1}^{2}=\sigma_{2}^{2}=\sigma_{3}^{2}=\sigma^{2}$. We investigated assigning separate prior distributions to $\nu^{\left(a\right)}$, $\nu^{\left(o\right)}$, and $\nu^{\left(s\right)}$, but this did not lead to any meaningful changes in the modeled estimates obtained from the shared mean in (1), meaning that the implicit assumption in ν in model (1) is that the data sources are exchangeable and are similarly susceptible to bias. Hence, the IDML specification has more flexibility in terms of handling contributions from the data sources to the likelihood than the BSL model. We additionally note that the components of ν in model (1) could be included as fixed effects as in model (2), but this will lead to the removal of the overall intercept term, λ, in model (1) to avoid the problem of multicollinearity.

It is straightforward to derive a country‐level model from Equations (1) and (2) by dropping the i subscript and excluding the terms: $\beta_{i},\;\phi_{it},\;\psi_{ij}$, and $\omega_{ijt}$ from the model. Although such models can be implemented by individual countries, these are of less interest in the current work as they do not allow borrowing strength across countries.

### 3.3 Specification of the Random Effects

We assume that the main effects $\beta_{i}$ and $\alpha_{j}$ are each iid normal random variables with mean zero and variances $\sigma_{\beta }^{2}$ and $\sigma_{\alpha }^{2}$, respectively. We note that it suffices to model country‐level variation as random as previous work (Utazi et al. 2020) showed a lack of significant spatial dependence in the data. Also, $\alpha_{j}$ can be specified to account for between‐vaccine correlation, but its correlation structure will need to be chosen carefully in this case, in light of the interaction terms included in the model. We assume a first‐order autoregressive (AR(1)) model for the temporal effect $\gamma_{t}$. That is,

(4) $$\gamma_{t}∼N\left(\rho \gamma_{t-1},\sigma_{\gamma }^{2}\right)$$

with $\gamma_{1}∼N\left(0,\sigma_{\gamma }^{2}/\left(1-\rho_{\gamma }^{2}\right)\right)$. The country–time interaction effect

$$\phi ∼N\left(0,{\left[\sigma_{\phi }^{-2}Q_{\phi }\left(\rho_{\phi }\right)\right]}^{-}\right)$$

where $\phi =(\phi_{11},\cdots ,\phi_{1T},\cdots ,\phi_{C1},\cdots ,\phi_{CT})$ and $Q_{\phi }\left(\rho_{\phi }\right)$ is a CT×CT structured matrix (Clayton 1996; Knorr‐Held 2000) specifying the nature of interdependence between the elements of ϕ, $\sigma_{\phi }^{-2}$ is an unknown precision parameter, $\rho_{\phi }$ is an autoregressive parameter and ${\left[.\right]}^{-}$ denotes the Moore–Penrose generalized inverse of a matrix. Following Clayton (1996), $Q_{\phi }\left(\rho_{\phi }\right)$ can be factorized as the Kronecker product of the structure matrices of the interacting main effects – $\beta_{i}$ and $\gamma_{t}$. Given the nature of these terms, here we assume a Type II interaction (Knorr‐Held 2000) such that $Q_{\phi }\left(\rho_{\phi }\right)=I_{C}⊗R_{\gamma }$, where $I_{C}$ is an identity matrix, and $R_{\gamma }$ is the neighborhood structure of an AR(1) process. This assumption implies that the temporal parameters for each country $\phi_{i,1:T}=\left(\phi_{i1},\cdots ,\phi_{iT}\right)$ follow an AR(1) process independent of all other countries. In other words, this two‐way interaction term captures temporal trends that are different from country to country and do not have any spatial structure. Similarly, the vaccine–time interaction is modeled as

$$\delta ∼N(0,{\left[\sigma_{\delta }^{-2}Q_{\delta }\left(\rho_{\delta }\right)\right]}^{-})$$

where $\delta ={\left(\delta_{11},\cdots ,\delta_{1T},\cdots ,\delta_{V1},\cdots ,\delta_{VT}\right)}^{\prime }$, $\sigma_{\delta }^{-2}$ is also an unknown precision parameter, and $\rho_{\delta }$ is an autoregressive parameter. We also assume a Type II interaction for this parameter such that $Q_{\delta }\left(\rho_{\delta }\right)=I_{V}⊗R_{\gamma }$. Here again, the structure of $Q_{\delta }$ implies that the temporal pattern for vaccine j represented by $\delta_{j,1:T}=\left(\delta_{j1},\cdots ,\delta_{jT}\right)$ is independent of those of other vaccines. Next, $\psi_{ij}$ represents the interaction of the iid terms $\beta_{i}$ and $\alpha_{j}$, hence we assume a Type I interaction (Knorr‐Held 2000) for it, such that

$$\psi ∼N(0,\sigma_{\psi }^{2}I_{CV})$$

where $\psi ={\left(\psi_{11},\cdots ,\psi_{1V},\cdots ,\psi_{C1},\cdots ,\delta_{CV}\right)}^{\prime }$ and $\sigma_{\psi }^{2}$ is a variance parameter. This interaction term models additional unstructured country–vaccine variation. Lastly, the three‐way interaction term, $\omega_{ijt}$, models temporal trends specific to each country–vaccine combination. We assume that

$$\omega ∼N(0,{\left[\sigma_{\omega }^{-2}Q_{\omega }\left(\rho_{\omega }\right)\right]}^{-})$$

where $\omega ={\left(\omega_{111},\cdots ,\omega_{CVT}\right)}^{\prime }$ and all the parameters are as defined previously. We also assume a Type‐II‐like interaction for ω such that $Q_{\omega }\left(\rho_{\omega }\right)=I_{C}⊗I_{V}⊗R_{\gamma }=I_{CV}⊗R_{\gamma }$, which implies that the temporal structure given to country i and vaccine j, represented by $\omega_{ij,1:T}=\left(\omega_{ij1},\cdots ,\omega ijT\right)$, is independent of those of other country–vaccine combinations.

## 4 Bayesian Inference and Computation

In this section, we describe details of implementation of the proposed models in a Bayesian setting. Let y denote all the observed or complete data. Also, let

$$\begin{array}{ccc} y^{\left(a\right)} & = & {\left(y_{111}^{\left(a\right)},\text{…},y_{\mathrm{CV}T_{1}}^{\left(a\right)}\right)}^{\prime }\\ y^{\left(o\right)} & = & {\left(y_{111}^{\left(o\right)},\text{…},y_{\mathrm{CV}T_{2}}^{\left(o\right)}\right)}^{\prime }\;\;\text{and}\\ y^{\left(s\right)} & = & {\left(y_{111}^{\left(s\right)},\text{…},y_{\mathrm{CV}T_{3}}^{\left(s\right)}\right)}^{\prime }, \end{array}$$

where $T_{1}=T_{2}=T_{3}=T$ in model (1). For the BSL model, let $\eta^{B}=(\lambda ,\;\beta ,\;\alpha ,\;\gamma$, ϕ,δ,ψ,ω,ν) denote a latent field comprising the intercept term and the joint distribution of all the parameters in the mean model $\mu =(\mu_{111},\cdots ,\mu_{CVT})$ (i.e., the random effects) given in Equation (1), $\theta_{1}^{B}=\sigma^{2}$ denote the variance of the Gaussian observations, and

$$\theta_{2}^{B}={\left(\sigma_{\beta }^{2},\;\sigma_{\alpha }^{2},\;\rho_{\gamma },\;\sigma_{\rho }^{2},\;\rho_{\phi },\;\sigma_{\phi }^{2},\;\rho_{\delta },\;\sigma_{\delta }^{2},\;\sigma_{\psi }^{2},\;\rho_{\omega },\;\sigma_{\omega }^{2},\sigma_{\nu }^{2}\right)}^{\prime }$$

denote the hyperparameters of the latent field $\eta^{B}$, i.e., the variances and autocorrelation parameters of the random effects. Similarly, for the IDML model, let

$$\begin{array}{ccc} \eta^{I} & = & (\lambda^{\left(a\right)},\;\lambda^{\left(o\right)},\;\lambda^{\left(s\right)},\;\beta ,\;\alpha ,\;\gamma ,\;\phi ,\;\delta ,\;\psi ,\;\omega ),\\ \theta_{1}^{I} & = & {\left(\sigma_{1}^{2},\;\sigma_{2}^{2},\;\sigma_{3}^{2}\right)}^{\prime }\;\text{and}\\ \theta_{2}^{I} & = & {\left(\sigma_{\beta }^{2},\;\sigma_{\alpha }^{2},\;\rho_{\gamma },\;\sigma_{\rho }^{2},\;\rho_{\phi },\;\sigma_{\phi }^{2},\;\rho_{\delta },\;\sigma_{\delta }^{2},\;\sigma_{\psi }^{2},\;\rho_{\omega },\;\sigma_{\omega }^{2}\right)}^{\prime }. \end{array}$$

We complete our Bayesian specification by placing appropriate prior distributions on the parameters as follows. For the BSL model, we assume the following prior distributions:

$$\begin{array}{ccc} \lambda & ∼ & N(0,1),\\ \sigma & ∼ & \text{Cauchy}(0,2)I(\sigma >0),\\ \sigma_{\nu } & ∼ & \text{Cauchy}\left(0,2\right)I\left(\sigma_{\nu }>0\right). \end{array}$$

For the IDML model, the prior distributions were

$$\lambda^{\left(a\right)}∼N\left(0,v_{1}\right);\;\lambda^{\left(o\right)}∼N\left(0,v_{2}\right);\;\lambda^{\left(s\right)}∼N\left(0,v_{3}\right);$$

$$\begin{array}{ccc} \sigma_{1} & ∼ & \text{Cauchy}\left(0,2\right)I\left(\sigma_{1}>0\right);\\ \sigma_{2} & ∼ & \text{Cauchy}\left(0,2\right)I\left(\sigma_{2}>0\right);\\ \sigma_{3} & ∼ & \text{Cauchy}\left(0,0.2\right)I\left(0\leq \sigma_{3}\leq 0.4\right). \end{array}$$

These prior distributions were chosen based on trial runs, during which we set $v_{1}=v_{2}=v_{3}=0.25$. The highly informative truncated Half‐Cauchy prior on $\sigma_{3}$ was chosen to attribute greater likelihood weight to survey estimates in the model subject to expert belief that these are more accurate and less biased (Cutts et al. 2016; Galles et al. 2021; Lim et al. 2008), and to adjust for the higher proportions of missingness in this data source, as noted previously. We note that by choosing this highly informative prior distribution for $\sigma_{3}$, the assessment of the relative contribution of each data source to the likelihood post‐model fitting is of less interest in our work. For all other parameters in both models, we used default uniform priors available in Stan (Stan Development Team 2015).

Letting $\theta^{B}={\left(\theta_{1}^{B},\theta_{2}^{B}\right)}^{\prime }$, the joint posterior distribution of the BSL model, based on a complete data likelihood, can be written as

(5)

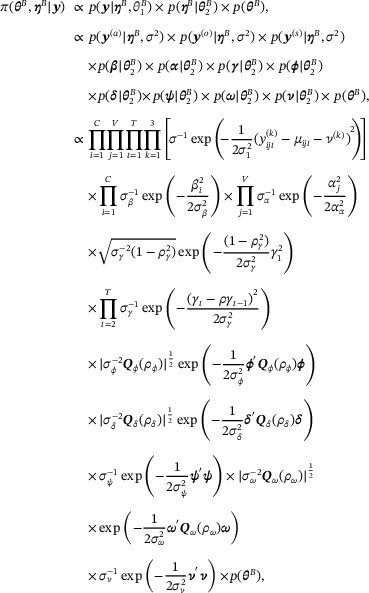

where $p(\theta^{B})$ denotes the joint prior distribution on the parameters. Given that we assumed that the parameters are a priori independent, $p(\theta^{B})$ simply represents the product of the prior distributions assigned to them.

Similarly, letting $\theta^{I}={\left(\theta_{1}^{I},\theta_{2}^{I}\right)}^{\prime }$, the joint posterior distribution of the IDML model can be written as

(6) $$\begin{array}{ccc} \pi (\theta^{I},\eta^{I}|y) & & \propto p\left(y|\eta^{I},\theta_{1}^{I}\right)\times p\left(\eta^{I}|\theta_{2}^{I}\right)\times p\left(\theta^{I}\right),\\ & & \propto p\left(y^{\left(a\right)}|\eta^{I},\theta_{1}^{I}\right)\times p\left(y^{\left(o\right)}|\eta^{I},\theta_{1}^{I}\right)\times p\left(y^{\left(s\right)}|\eta^{I},\theta_{1}^{I}\right)\\ & & \;\times \;p\left(\beta ,\alpha ,\gamma ,\phi ,\delta ,\psi ,\omega |\theta_{2}^{I}\right)\times p(\theta^{I}),\\ & & \propto \prod_{i=1}^{C}\prod_{j=1}^{V}\left[\prod_{t_{1}=1}^{T_{1}}\sigma_{1}^{-1}\exp\left(-\frac{1}{2\sigma_{1}^{2}}{\left(y_{\mathrm{ij}t_{1}}^{\left(a\right)}-\lambda^{\left(a\right)}-\mu_{\mathrm{ij}t_{1}}\right)}^{2}\right)\right.\\ & & \;\times \prod_{t_{2}=1}^{T_{2}}\sigma_{2}^{-1}\exp\left(-\frac{1}{2\sigma_{2}^{2}}{\left(y_{\mathrm{ij}t_{2}}^{\left(o\right)}-\lambda^{\left(o\right)}-\mu_{\mathrm{ij}t_{2}}\right)}^{2}\right)\\ & & \;\left.\times \prod_{t_{3}=1}^{T_{3}}\sigma_{3}^{-1}\exp\left(-\frac{1}{2\sigma_{3}^{2}}{\left(y_{\mathrm{ij}t_{3}}^{\left(s\right)}-\lambda^{\left(s\right)}-\mu_{\mathrm{ij}t_{3}}\right)}^{2}\right)\right]\\ & & \;\times p\left(\beta ,\alpha ,\gamma ,\phi ,\delta ,\psi ,\omega |\theta_{2}^{I}\right)\times p\left(\theta^{I}\right), \end{array}$$

where $p\left(\beta ,\alpha ,\gamma ,\phi ,\delta ,\psi ,\omega |\theta_{2}^{I}\right)=p\left(\beta |\theta_{2}^{I}\right)\times p\left(\alpha |\theta_{2}^{I}\right)\times p\left(\gamma |\theta_{2}^{I}\right)\times p\left(\phi |\theta_{2}^{I}\right)\times p\left(\delta |\theta_{2}^{I}\right)\times p\left(\psi |\theta_{2}^{I}\right)\times p\left(\omega |\theta_{2}^{I}\right)$, which are all the same as corresponding expressions provided in Equation (5).

The goal of inference in Equations (5) and (6) is to estimate the posterior distributions of the components of $\eta^{B}$, $\eta^{I}$, $\theta^{B}$, $\theta^{I}$ and, for Equation (5), the missing values in y, all of which are in turn used to obtain the underlying, smoothed coverage estimates $\mu_{ijt},\;\forall \;i\in \left\{1,\cdots ,C\right\},\;j\in \left\{1,\cdots ,V\right\}\text{and}\;t\in \left\{1,\cdots ,T\right\}$ as given in Equations (1) and (3). As noted previously, the missing values in y in Equation (5) are estimated through their posterior predictive distributions in a Bayesian context (Gelman et al. 2013).

We fitted both models by running MCMC using the NUTS (No‐U‐Turn Sampler) algorithm (Hoffman and Gelman 2014) within the Stan package in R Stan Development Team (2020). We implemented four chains, each of which was run for 4000 iterations, including a burn‐in of 2000 iterations. We assessed convergence using the MCMC convergence statistic, $\hat{R}$, which we ensured was below 1.05 (Vehtari et al. 2021) for each parameter in the models, and by examining the trace plots of the parameters. We also provide an R package for implementing the proposed models, further details of which are provided in Section 7.

### 4.1 Smoothed Overall Estimates

Note that the mean models in (1) and (3) are well defined for both the BSL and IDML models as discussed above. We use the posterior distribution of $\mu_{ijt}$ to produce our model‐based vaccination coverage estimates. After eliminating the source‐specific bias components, $\nu^{\left(k\right)}$ and $\lambda^{\left(k\right)}$ in our notation, we obtain the coverage estimate as follows. We suppose that the inverse logit‐transform

(7) $$p_{ijt}=\frac{1}{1+\exp\left(-\mu_{ijt}\right)}$$

is the source‐free and true bias‐corrected vaccination coverage proportion.

The posterior distribution of $p_{ijt}$ given all the data is summarized to provide source‐free estimates of vaccination coverage. The posterior distribution of $p_{ijt}$ is easy to calculate using MCMC sampling. For example, we obtain MCMC samples $\theta^{\left(\ell \right)}$, ℓ=1,…,L for all the unknown parameters and random effects and missing data collectively denoted by θ. Using $\theta^{\left(\ell \right)}$ we evaluate $\mu_{ijt}^{\left(\ell \right)}$ and subsequently $p_{ijt}^{\left(\ell \right)}$ for ℓ=1,…,L. These samples are then used to estimate the true $p_{ijt}$ along with the uncertainty estimates. Note that these uncertainty estimates are obtained exactly for vaccination coverage at the original scale.

### 4.2 Prediction and Aggregation to the Regional Level

In addition to parameter estimation, it is often the goal in Bayesian analysis to estimate missing observations or to predict future observations. Typically, Bayesian in‐sample and out‐of‐sample prediction (the latter is also known as forecasting) are both based on the posterior predictive distribution. For example, the one‐step‐ahead prediction at any time point t can be obtained by evaluating the conditional distribution of $p_{ijt+1}$ given all the data y. According to (7), we have

$$p_{\mathrm{ijt}+1}=\frac{1}{1+\exp\left(-\mu_{\mathrm{ijt}+1}\right)},$$

where, for example, for the BSL model,

$$\mu_{ijt+1}=\lambda +\beta_{i}+\alpha_{j}+\gamma_{t+1}+\phi_{it+1}+\delta_{jt+1}+\psi_{ij}+\omega_{ijt+1}$$

from (1). Hence to predict $p_{ijt+1}$ we also need the values of the time‐advanced parameters $\gamma_{t+1}$, $\delta_{jt+1}$ and $\omega_{ijt+1}$ at time t. For a given t within the modeled time period T, these parameters are already sampled within the implemented MCMC scheme. For t≥T, we use the assumed model dynamics, e.g., (4), to sample these parameters. That is, we set

$$\gamma_{t+1}^{\left(\ell \right)}∼N\left(\rho^{\left(\ell \right)}\gamma_{t}^{\left(\ell \right)},\sigma_{\gamma }^{2(\ell )}\right)$$

if $\gamma_{t+1}$ has not been sampled already. The other dynamic parameters are treated similarly. Hence, to estimate (or to predict if t>T) $p_{ijt}$, using either of the proposed models, we evaluate the posterior distribution of $p_{ijt}$ given y by drawing samples $p_{ijt}^{\left(\ell \right)}$ for ℓ=1,…,L for a large number of MCMC replicates L.

In Stan, these out‐of‐sample predictions can be computed post model‐fitting. As with in‐sample estimation, the final predictions can be obtained by summarizing the inverse logit of the posterior samples of $\mu_{ij(t+1)}^{\left(\ell \right)}$. We note that by default, in‐sample estimates of $\mu_{ijt}$ are estimated for desired in‐sample country–vaccine–time combinations even when no data are observed for these cases, since $\mu_{ijt}$ is estimated ∀i∈{1,⋯,C},j∈{1,⋯,V},t∈{1,⋯,T}. As explained earlier, in‐sample estimates of $\mu_{ijt}$ are processed post‐model‐fitting using yovi data to obtain modeled estimates for desired country–vaccine–time combinations.

Further, we obtain modeled estimates of immunization coverage for each WHO region as population‐weighted averages taken over all the countries falling within the region. That is, for region $R_{r}\;\left(r=1,\cdots ,6\right)$, vaccine j, time t, and posterior sample ℓ,

(8) $$p_{rjt}^{\left(\ell \right)}\left(R\right)=\sum_{i\in R_{r}}p_{ijt}^{\left(\ell \right)}\times q_{i}^{r}$$

where $q_{i}^{r}$ is the proportion of surviving infants or target population for MCV2 of region $R_{r}$ living in country i. It is straightforward to compute Equation (8) using the posterior distributions of $p_{ijt}$ under each model.

### 4.3 Model Comparison, Evaluation, and Validation

To choose between the proposed models in our application, we considered the Watanabe‐Akaike information criterion (WAIC) (Watanabe 2013). The WAIC is a fully Bayesian criterion that is based on the log of the predictive density for each data point, hence it assesses the ability of the fitted model to predict the input data. Accessible discussions regarding WAIC are provided by Gelman et al. (2014) and Sahu (2022).

To further evaluate the ability of the proposed models to predict the in‐sample and out‐of‐sample data (i.e., $p_{ijt}$) in a simulation experiment (due to lack of true values of $p_{ijt}$ in our application – see Section 5), we computed the following metrics:

$$\begin{array}{ccc} \mathrm{Average}\mathrm{bias}:AvBias & = & \frac{1}{m}\sum_{k=1}^{m}\left({\hat{p}}_{k}-p_{k}\right)\\ \mathrm{Root}\mathrm{mean}\mathrm{squared}\mathrm{error}:RMSE & = & \sqrt{\frac{1}{m}\sum_{k=1}^{m}{\left({\hat{p}}_{k}-p_{k}\right)}^{2}}\\ \mathrm{Mean}\mathrm{absolute}\mathrm{error}:MAE & = & \frac{1}{m}\sum_{k=1}^{m}\left|{\hat{p}}_{k}-p_{k}\right|\\ 95\%\mathrm{coverage}:95\%\text{coverage} & = & 100\times \sum_{k=1}^{m}I\left({\hat{p}}_{k}^{l}\leq p_{k}\leq {\hat{p}}_{k}^{u}\right) \end{array}$$

and the Pearson's correlation between observed and predicted values. Here, m denotes all the values of $p_{\left(.\right)}$ used for validation (across all vaccines, countries and time points), ${\hat{p}}_{k}$ and $p_{k}$ are the predicted (i.e., the posterior means) and observed values, ${\hat{p}}_{k}^{l}$ and ${\hat{p}}_{k}^{u}$ are the lower and upper limits of the 95% credible intervals of the predictions and I(.) is an indicator function. The actual coverage of the 95% prediction intervals assesses the accuracy of the uncertainty estimates associated with the predictions, while all the other metrics evaluate the accuracy of the point estimates. The closer the 95% coverage rates are to the nominal value of 95%, the better the predictions. Similarly, the closer the root mean squared error (RMSE), mean absolute error (MAE), and average bias (AvBias) (in absolute value) are to zero, the better the predictions. Correlations close to 1 indicate strong predictive power.

## 5 Simulation Study

We conducted a simulation study to examine the predictive performance of the proposed models with respect to in‐ and out‐of‐sample estimation of $p_{ijt}$. Consistent with our modeling philosophy, this allowed us to evaluate how well the proposed models can combine information from all three (inherently biased) data sources to estimate $p_{ijt}$ when its true value is known. In the study, we set C=20,T=20 and V=5, mimicking a moderately‐sized WHO region. We then used the following true parameter values to generate estimates of $\mu_{ijt}$, as described in Equations (1) and (3) for the BSL and IDML models, respectively: $\sigma_{\beta }^{2}=1,\;\sigma_{\alpha }^{2}=1,\;\rho_{\gamma }=0.5,\;\sigma_{\gamma }^{2}=1,\;\rho_{\phi }=0.3,\;\sigma_{\phi }^{2}=0.25,\;\rho_{\delta }=0.4,\sigma_{\delta }^{2}=0.64,\;\sigma_{\psi }^{2}=1,\;\rho_{\omega }=0.7,\;\sigma_{\omega }^{2}=0.64$. Additionally, for the IDML model, we set $\lambda^{\left(a\right)}=0.07,\;\lambda^{\left(o\right)}=0.02,\;\lambda^{\left(s\right)}=0.05$. For the BSL model, we set $\sigma^{2}=1$ and λ equal to the means of the corresponding parameters in the IDML model, i.e., λ=0.05.

Owing to the key role that the parameters $\sigma_{1}^{2},\;\sigma_{2}^{2},\;\sigma_{3}^{2}$ and $\sigma_{\nu }^{2}$ play in capturing the amount of residual variability or bias attributable to the different data sources in the proposed models, we examined the effect of their varying values on the estimation of $p_{ijt}$ (Equation (7)) by considering the following scenarios.

**Scenario 1:** Variance of $\nu^{\left(k\right)}\left(\text{i.e.,}\;\sigma_{\nu }^{2}\right)$ in BSL model set equal to the average of the conditional variances of admin ($\sigma_{1}^{2}$), official ($\sigma_{2}^{2}$) and survey estimates ($\sigma_{3}^{2}$) in IDML model ($\sigma_{1}^{2}=1,\;\sigma_{2}^{2}=0.64,\;\sigma_{3}^{2}=0.16$ and $\sigma_{\nu }^{2}=0.6$).

**Scenario 2:** Large differences between the conditional variances of admin, official and survey estimates and larger variance for $\nu^{\left(k\right)}(\sigma_{1}^{2}=9,\;\sigma_{2}^{2}=4,\;\sigma_{3}^{2}=0.25$ and $\sigma_{\nu }^{2}=4$).

**Scenario 3:** Same conditional variances for admin, official, and survey estimates and smaller variance for $\nu^{\left(k\right)}\;(\sigma_{1}^{2}=1,\;\sigma_{2}^{2}=1,\;\sigma_{3}^{2}=1$ and $\sigma_{\nu }^{2}=0.1)$.

These true parameter estimates were chosen to encourage, as much as possible, an even distribution of values of $p_{ijt}$ on the unit interval. Adding the other components of Equations (1) and (2) to the simulated values of $\mu_{ijt}$, we obtained the simulated admin, official, and survey estimates for all values of C, T, and V. To mimic the patterns of missingness in MCV2 and PCV3 in our application (see, e.g., Supplementary Figure S4), we randomly selected t=10 or t=15 as the starting points for the observations for the last two vaccines. Further, we deleted 15% of each of the simulated admin and official data and 20% of the simulated survey data to reflect the overall pattern of missing values in our application. In all, for each model, the simulated data had a total of 3864 observations, 68% of which were either admin or official data, while the remaining 32% were survey data.

We placed the same prior distributions as before on both models, except that for the IDML model, we placed a Half‐Cauchy(0,2) prior on $\sigma_{3}$, making it the same as the priors on $\sigma_{1}$ and $\sigma_{2}$, since the goal here is to compare both models under similar conditions (the effect of the prior specification on $\sigma_{3}$ on the performance of the IDML model is examined further in Section 6). We set the starting point for the one‐ and two‐step ahead predictions at t=11, meaning that we would use the first 10 observations as base years and then make predictions for the remaining ten time points, i.e., t=11,⋯,20.

In Table 2, we report the results of the study showing the validation statistics computed using the true and modeled estimates of $p_{ijt}$ under each model. Also, in Supplementary Figure S7, we show examples of the simulated data and the modeled estimates (in‐sample prediction) for five countries and three vaccines under scenario 1. For in‐sample prediction, the IDML model generally outperformed the BSL model across the three scenarios, yielding both more accurate point and uncertainty estimates. In particular, we observe that the BSL model had relatively large AvBias estimates under Scenarios 1 and 2, demonstrating that it under‐predicted the true values of $p_{ijt}$ in those cases (see, e.g., Supplementary Figure S7). This under‐prediction also resulted in very poor 95% coverage under Scenario 2, suggesting that the BSL model may not be well suited for in‐sample prediction when there is a considerable amount of variation (or bias) arising from the different data sources. This poorer performance by the BSL model persisted even when $\sigma_{t}$ was set to 0.1 in scenario 1 to encourage lesser variability in the true values of $p_{ijt}$ across the countries (Supplementary Figure S8).

**TABLE 2:** Simulation study: validation statistics for in‐sample, one‐step‐ahead and two‐step ahead predictions.

Validation metrics	In‐sample prediction					
	Scenario 1		Scenario 2		Scenario 3	
	**BSL**	**IDML**	**BSL**	**IDML**	**BSL**	**IDML**
AvBias	−8.81	**0.79**	−9.25	**0.51**	**−1.00**	1.12
RMSE	11.22	**3.47**	11.95	**3.87**	5.74	**4.98**
MAE	9.09	**1.73**	9.50	**1.87**	3.78	**2.92**
95% coverage	79.00	**98.50**	28.20	**98.40**	74.10	**98.00**
Correlation	0.98	**0.99**	0.97	**0.99**	0.98	**0.99**
	**One‐step‐ahead prediction**					
AvBias	−3.53	**−2.11**	**−1.58**	−2.54	**−0.21**	−1.82
RMSE	21.40	**18.60**	19.40	**18.55**	20.74	**19.10**
MAE	18.54	**15.82**	15.89	**15.74**	17.29	**16.20**
95% coverage	**99.00**	99.70	**98.70**	99.80	**98.20**	99.70
Correlation	0.74	**0.81**	0.79	**0.81**	0.75	**0.80**
	**Two‐step‐ahead prediction**					
AvBias	−3.33	**−2.78**	**−2.20**	−3.19	**−1.23**	−2.49
RMSE	21.38	**19.42**	20.10	**19.37**	21.34	**19.87**
MAE	18.36	**16.70**	16.68	**16.64**	18.01	**17.00**
95% coverage	**98.79**	99.74	**98.58**	99.79	**98.47**	99.74
Correlation	0.74	**0.79**	0.77	**0.79**	0.73	**0.78**

The better result is shown in bold in each case.

For out‐of‐sample prediction, the RMSE, MAE and correlation estimates are worse off as expected (Table 2). However, mixed results were obtained when considering AvBias and 95% coverage. We note that the high values of the actual 95% coverage for both models are not surprising since out‐of‐sample predictions are often made with wider uncertainty intervals (Supplementary Figures S9–S12 ‐ these also include the 80% and 50% credible intervals for comparison). Also, these high values are partly due to the large variation in the source‐specific data and the true values of $p_{ijt}$. We rather focus on using the other metrics to evaluate the out‐of‐sample performance of the models. Again, the IDML model generally yielded more accurate estimates of $p_{ijt}$ than the BSL model according to the RMSE, MAE, and correlation, although it tended to produce relatively more biased estimates, especially under scenarios 1 and 2. Unlike in‐sample prediction, the performance of the BSL model appears to be more stable and more comparable to that of the IDML model across the three scenarios in out‐of‐sample prediction. This is because the out‐of‐sample predictions mostly captured the average trend in the observations under both models. Overall, these results show that the IDML model outperformed the BSL model and is, therefore, better suited for both in‐ and out‐of‐sample prediction with regard to the estimation of the true, underlying coverage estimates, $p_{ijt}$.

## 6 Results

Here, we present and discuss the results of the application of the proposed methodology to produce modeled ENIC for all WHO Member States.

### 6.1 Model Choice, Validation, and Parameter Estimates

We first fitted both the BSL and IDML models to the national immunization coverage data to further examine their performance and suitability for the data. With the IDML model, we considered two cases – one in which we placed a Half‐Cauchy(0,2) prior on $\sigma_{3}$ to depict an unrestricted scenario, and the other where we placed a truncated Half‐Cauchy(0,0.2) prior on $\sigma_{3}$ to improve the contribution of survey data to the likelihood. For the BSL model, we used the same priors described previously.

In Table 3, we report the WAIC statistics for these models, which reveal an interesting pattern. With a less restrictive Half‐Cauchy(0,2) prior on $\sigma_{3}$, the IDML model clearly outperformed the BSL model in all cases except in the AFR region. When examining the contributions of the penalty and goodness‐of‐fit (GOF) terms to the calculated WAIC, we observe that although the BSL model has smaller penalties as expected, since it includes a fewer number of parameters, the IDML model consistently provided better fits according to the GOF statistics. However, the use of a truncated Half‐Cauchy(0,0.2) prior on $\sigma_{3}$ in the IDML model, though deliberate, resulted in poorer fits to the data since this prior biases the modeled estimates toward survey data. Hence, the BSL model yielded smaller WAIC statistics in this case. These results further demonstrate the flexibility of the IDML model to adjust the modeled estimates based on expert opinions. All modeled outputs and results presented in the remainder of this work are, therefore, based on the IDML model only.

**TABLE 3:** WAIC statistics for the base single likelihood (BSL) and irregular data multiple likelihood (IDML) models for each WHO region.

Region	BSL			IDML		
	GOF	penalty	WAIC	GOF	penalty	WAIC
Half‐Cauchy(0,2) prior placed on $\sigma_{3}$ in the IDML model						
AFR	21524.8	**1053.9**	**23632.6**	**20905.1**	1543.5	23992.1
AMR	8024.3	**1314.8**	10653.9	**1370.3**	2242.1	**5854.5**
EMR	6905.6	**733.0**	8371.6	**5699.9**	1093.4	**7886.7**
EUR	7990.8	**1453.4**	10897.6	**5416.9**	2325.0	**10066.9**
SEAR	5094.2	**189.4**	5473.0	**4892.6**	269.6	**5431.8**
WPR	8656.1	**850.7**	10357.5	**7276.0**	1302.8	**9881.6**
Truncated Half‐Cauchy(0,0.2) prior placed on $\sigma_{3}$ in the IDML model						
AFR	**21524.8**	**1053.9**	**23632.6**	22530.8	1420.1	25371.0
AMR	**6163.8**	2599.3	11362.4	8024.3	**1314.8**	**10653.9**
EMR	**6905.6**	**733.0**	**8371.6**	7019.2	1113.2	9245.6
EUR	7990.8	**1453.4**	**10897.6**	**6779.1**	2438.1	11655.3
SEAR	5094.2	**189.4**	**5473.0**	**4963.7**	357.8	5679.3
WPR	**8656.1**	**850.7**	**10357.5**	9141.6	1237.8	11617.2

The better result is shown in bold in each case.

In Table 4, we report estimates of parameters of the model for AFR region. Parameter estimates for other regions are presented in Supplementary Tables S2–S6. We observe that estimates of the source‐specific intercept terms, ${\hat{\lambda }}^{\left(a\right)}$ and ${\hat{\lambda }}^{\left(o\right)}$), are consistently positive while those of ${\hat{\lambda }}^{\left(s\right)}$ are consistently negative in all the regions. However, only ${\hat{\lambda }}^{\left(s\right)}$ was significant in some of the regions. This further demonstrates that survey data where available, on average, tend to have lower values than admin and official data. Estimates of the residual standard deviation for survey data are all close to the upper bound of the prior placed on $\sigma_{3}$, demonstrating the strong effect of the prior in the fitted models. However, ${\hat{\sigma }}_{1}$ and ${\hat{\sigma }}_{2}$ are considerably higher than ${\hat{\sigma }}_{3}$ in all the regions except AMR and EUR. In both regions, the estimates of these parameters are very close, indicating that survey estimates are, on average, more likely to have greater or similar variation as other data sources in both regions (see e.g., Figure 1).

**TABLE 4:** Posterior estimates of the parameters of the irregular data model (IDML) for the AFR region.

Parameter	Mean	Std. dev.	2.5%	50%	97.5%
${\hat{\lambda }}^{\left(a\right)}$	0.4922	0.2868	−0.0728	0.4887	1.0557
${\hat{\lambda }}^{\left(o\right)}$	0.3661	0.2870	−0.2023	0.3656	0.9286
${\hat{\lambda }}^{\left(s\right)}$	−0.4421	0.2869	−1.0068	−0.4421	0.1195
${\hat{\sigma }}_{1}$	1.3951	0.0209	1.3546	1.3947	1.4364
${\hat{\sigma }}_{2}$	1.1700	0.0182	1.1352	1.1698	1.2059
${\hat{\sigma }}_{3}$	0.3992	0.0008	0.3970	0.3994	0.4000
${\hat{\sigma }}_{\beta }$	0.8900	0.1091	0.6982	0.8803	1.1283
${\hat{\sigma }}_{\alpha }$	1.5378	1.1932	0.0950	1.3068	4.5089
${\hat{\rho }}_{\gamma }$	0.5084	0.5363	−0.7621	0.7546	0.9963
${\hat{\sigma }}_{\gamma }$	0.0786	0.0591	0.0060	0.0673	0.2061
${\hat{\rho }}_{\phi }$	0.4089	0.0714	0.2682	0.4113	0.5445
${\hat{\sigma }}_{\phi }$	0.5260	0.0235	0.4801	0.5259	0.5714
${\hat{\rho }}_{\delta }$	0.9712	0.0255	0.8992	0.9791	0.9963
${\hat{\sigma }}_{\delta }$	0.2303	0.0356	0.1629	0.2297	0.3029
${\hat{\sigma }}_{\psi }$	0.3726	0.0569	0.2498	0.3753	0.4774
${\hat{\rho }}_{\omega }$	0.7106	0.0563	0.5961	0.7131	0.8104
${\hat{\sigma }}_{\omega }$	0.3823	0.0299	0.3249	0.3819	0.4428

When considering the main effects – $\beta_{i}$, $\alpha_{j}$, and $\gamma_{t}$ – these results indicate that in the AFR region, the vaccine random effect, $\alpha_{j}$, accounted for much (74.8%) of the total variation $\left({\hat{\sigma }}_{\beta }^{2}+{\hat{\sigma }}_{\alpha }^{2}+{\hat{\sigma }}_{\gamma }^{2}\right)$ explained by these terms. For other regions, the vaccine random effect accounted for between 79.1% and 99.6% of the total variation explained by the main effects. This demonstrates substantial variation in coverage levels between the vaccines across all the regions. There is also considerable variation in coverage levels among countries within each region, as the estimates of $\sigma_{\beta }$ show. However, the temporal main effect, $\gamma_{t}$, explains very little variation in the data in each region (except for the AFR region), which is likely due to the significant effect of temporally correlated interaction terms in the models. Similarly, when considering the estimated variances of the interaction terms, the country–time interaction, $\phi_{it}$, explained the most variation in the data compared to other interaction terms in the AFR region. This was also the case in the SEAR region. For AMR, EMR and WPR regions, the most variation was explained by the country–vaccine–time interaction $\omega_{ijt}$, whereas for the EUR region, this was explained by the country–vaccine interaction, $\psi_{ij}$. In all the regions, the vaccine–time interaction, $\delta_{jt}$, explained the least variation in the data compared to other interaction terms. These results indicate the presence of strong within‐country trends in the data. Estimates of the autoregressive parameter for $\gamma_{t}$, ${\hat{\rho }}_{\gamma }$, are not significant across all the regions, further indicating the little contribution of this global term in the fitted models (although its inclusion supports the structure of the interaction terms). However, the autocorrelation parameters of all the time‐varying interaction terms are estimated to be significantly positive in all the regions, suggesting strong positive temporal trends which are tied to other sources of variation (country and vaccine) in the data.

### 6.2 Modeled Estimates of National Immunization Coverage and Comparisons With WUENIC Estimates

In Figure 3 and Supplementary Figures S13–S17, we present plots of modeled estimates of coverage and associated uncertainties for EMR and other WHO regions, respectively. Time series plots of the modeled estimates overlaid with the input admin, official, and survey data, as well as corresponding WUENIC estimates (2020 revision published in 2021) are also shown in Supplementary Figure S18. In general, we observe that the patterns in immunization coverage are similar for DTP1, DTP3, and MCV1 due to these vaccines being introduced much earlier (since the 1970s) in the study countries, and therefore exhibit more stable trends, compared to MCV2 and PCV3. Both newer vaccines tend to have different patterns since these are mostly driven by the length of time since introduction and the speed of uptake. We also note that the fitted models produced plausible estimates of coverage relative to the input data and adjust well to survey data in some cases for the example countries, as shown in Supplementary Figure S18. For EMR, coverage appears high and more stable in many countries, although some countries had lower coverage at the beginning of the study period (e.g., Afghanistan (AFG) and Djibouti (DJI)), but made substantial progress, which appears to stagnate in later years. The uncertainty estimates are low in most cases but generally higher for country–vaccine–time combinations for which input data were scanty or unavailable (e.g., DJI‐MCV2 in Figure 3) and for out‐of‐sample predictions. In general, the predictions for 2020 and 2021 show changes in coverage, but not substantially from the preceding years. We note that these predictions did not take into account any disruptions to routine immunization caused by the pandemic. Hence, they represent a counterfactual non‐pandemic scenario.

**FIGURE 3:**
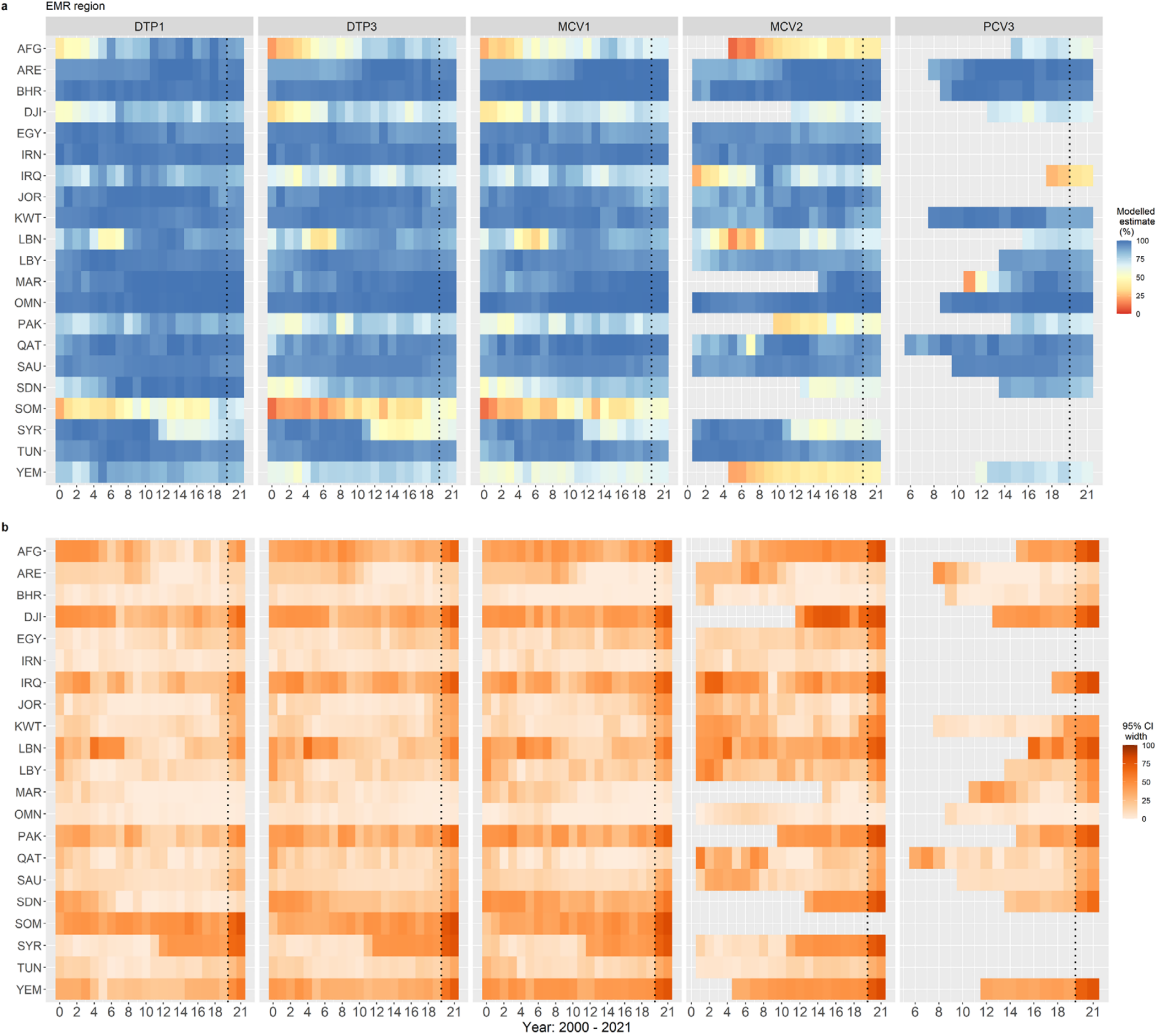
Modeled estimates of immunization coverage (a) and corresponding uncertainty estimates (b) for the EMR region. Predictions for 2021 and 2022 are shown on the right‐hand side of the black dotted vertical lines.

In Figure 4 and Supplementary Figures S19–S25, we show comparisons between WUENIC and the modeled estimates for the period 2000 to 2019. As noted previously, the WUENIC estimates were produced using a deterministic computational logic approach that involved supplementing administrative and official estimates using survey estimates (Burton et al. 2009, 2012; World Health Organization and United Nations Children's Fund 2022). There is generally a good level of correspondence between both estimates across the regions despite the differences in the methodologies used to produce them. The best correlations seem to have occurred in AFR, EMR, and SEAR regions, but the largest differences also occurred in AFR where the modeled estimates tend to be higher than WUENIC in some countries for DTP1, DTP3, and MCV1. At the global level, we obtained a median difference of −1.90% with an interquartile range of 5.44% (Supplementary Table S7) between WUENIC and the modeled estimates. The highest median difference (in absolute value) was observed in AMR (‐4.62%) while the largest interquartile range was observed in AFR (10.35%). Overall, these results strongly indicate that the modeled estimates are close to WUENIC.

**FIGURE 4:**
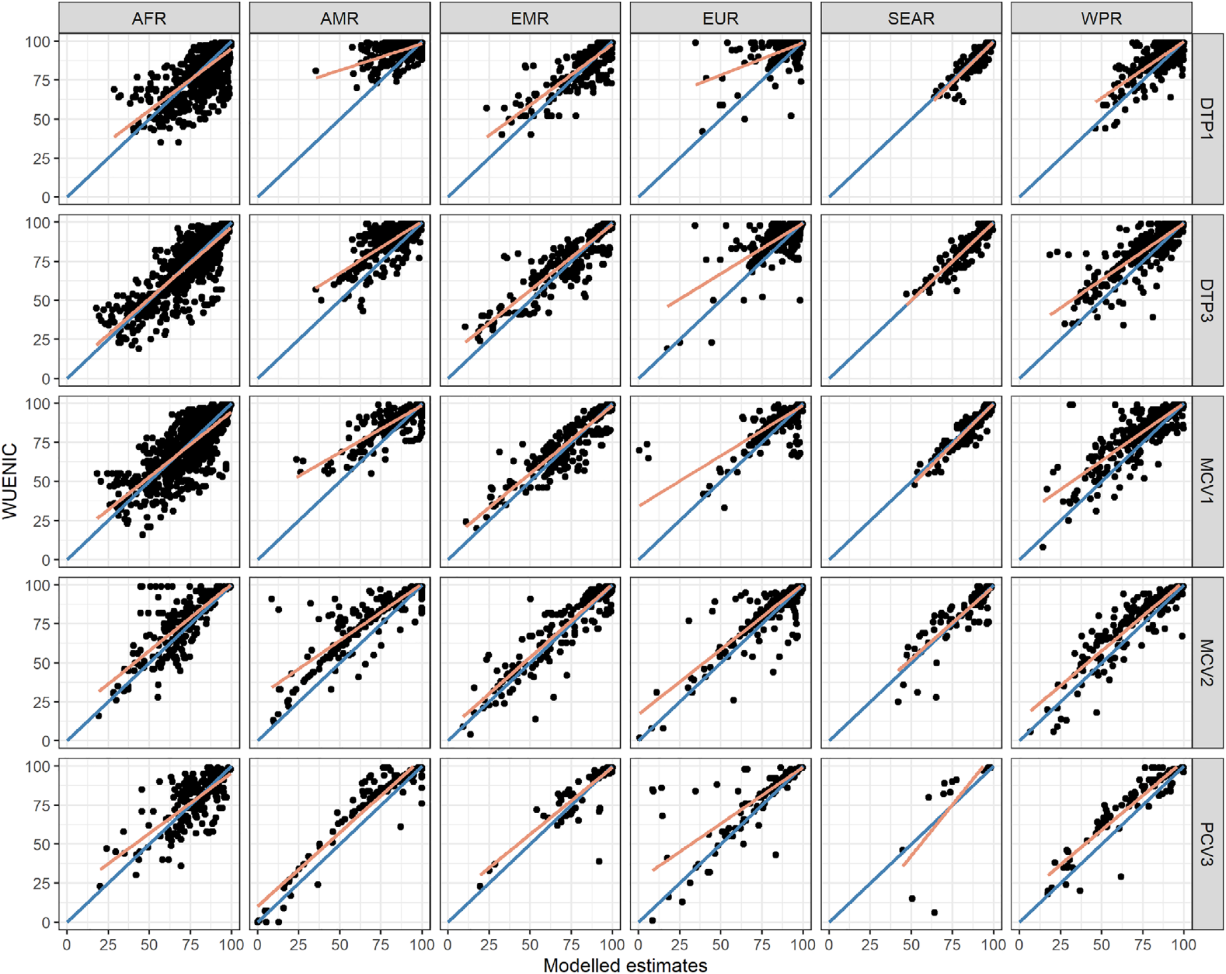
Plots of WUENIC versus modeled estimates by WHO regions and vaccine type. The blue lines illustrate a perfect agreement between both estimates while the light red lines are simple least square fits to the estimates within each region.

Furthermore, the trends in the regional estimates of immunization coverage are displayed in Supplementary Figure S26a,b. For the AFR region, for example, all five vaccines except MCV2 showed increasing trends which appear to level off or regress toward the end of the study period. Similar or different patterns were observed in other regions. The uncertainties associated with these regional estimates show robust estimation in most cases.

## 7 Software

We developed the imcover package in the R statistical programming language to support the proposed Bayesian modeling approaches for immunization coverage estimation. imcover is an interface to the Stan programming language implementing the NUTS. The package includes functionality to replicate the analyses described in Section 3 using both the BSL and IDML models. The package is designed to support reanalysis of the WUENIC data with functionality to retrieve input data from the WHO website, process the files, fit models and produce coverage estimates for required country‐ and regional–vaccine–time combinations. The software is available from https://wpgp.github.io/imcover/ and is described in more detail along with a full processing script in the supplementary file (Sections 2 and 4).

## 8 Discussion

We have laid out a new methodology for producing ENIC, as an alternative or complement to the WUENIC approach. Our methodology is based on a Bayesian hierarchical model that accounts for the full range of uncertainties present in both the input data and the modeled estimates. We adopted a progressive approach in our model development and investigated two candidate models – the BSL and IDML models – which differ in how missing data are treated in their likelihoods. The methodology is implemented in a user‐friendly R package, imcover. We found that the IDML model, which provides a lower dimensional parameter space under missingness, and hence lower posterior uncertainty, performed better in most cases in terms of model fit and predictive performance (Table 3). Also, regarding computational efficiency, the IDML model is preferable as it takes an average of 1.6 hours to run 4,000 iterations (including a burn‐in of 2000 iterations) for each WHO region on a high‐specification computer. This is roughly half of the time required to run the BSL model.

The work presented here builds upon and improves our previous study (Utazi et al. 2020), which applied aspects of the WUENIC computational logic (e.g., denominator adjustment for admin estimates and choosing survey data when the differences between survey estimates and denominator‐adjusted admin estimates were >10%) to process and harmonize the multiple input data. The processed data were then modeled using a BHM, similar to the models developed in the current work, but only accounting for (random) spatial, temporal, and vaccine‐related variations and their interactions, to obtain smoothed coverage estimates and associated uncertainties. While this earlier approach produced coverage estimates that were similar to WUENIC, the ad hoc method adopted in combining information from the multiple input data prior to model fitting meant that the full range of uncertainty inherent in the data was not properly accounted for. The current methodology addresses this limitation by explicitly modeling the uncertainty associated with each data source using appropriate probability distributions.

Another model‐based approach for producing ENIC was proposed by Lim et al. (2008), but this relied primarily on survey data and was implemented in a non‐Bayesian framework that lacked the flexibility to incorporate prior beliefs into the modeling process. More recently, Galles et al. (2021) used a model‐based strategy that integrated official and survey data through a multi‐step spatio‐temporal Gaussian process regression. Their approach involved first adjusting official data for bias using survey data, followed by joint modeling of the bias‐adjusted official and survey data. In contrast to both studies, our modeling framework combines information from all three data sources within a unified Bayesian hierarchical structure, assuming conditional independence. This allowed us to produce smoothed, bias‐corrected coverage estimates while capturing the expert belief that survey data are more reliable through our prior specification, instead of treating survey data as the “gold‐standard” or reference data source as in both studies.

The quality of the modeled estimates produced in our work is largely dependent upon the quality and degree of completeness of the input data, as also acknowledged in World Health Organization (2019b). First, as we noted in Section 2, all three input data sets were not simultaneously available for desired country–time–vaccine combinations, with survey estimates being the most incomplete data source and unequally distributed over time and by WHO region. There were also instances where no data were available from all three data sources. As we observed in Section 6, the proposed methodology is more likely to produce more robust and more precise estimates when more (accurate) input data are used for model‐fitting. Second, the different input data sets have their inherent biases (e.g., admin estimates being greater than 100), which are likely the result of inaccurate denominator and/or numerator estimates, large differences between consecutive coverage estimates (in time) and recall bias associated with survey data for multi‐dose vaccines (Cutts et al. 2016). A complete overview and analysis of data quality issues associated with these data sources are provided in Rau et al. (2022); Stashko et al. (2019). Although we implemented some ad hoc measures to correct these biases wherever possible, e.g., recall‐bias adjustment for survey estimates of DTP3 and PCV3 and rounding down of administrative estimates greater than 100 while persevering the differential between multi‐dose vaccines, they are better addressed at the point of data collection and summarization. Hence, efforts should be intensified within countries to improve the quality of data collected via these sources as has been recommended by global advisory bodies (Scobie et al. 2020).

Wherever possible, the data processing steps presented here could be much improved to deal with any remaining data quality issues prior to model‐fitting, as we have highlighted previously. It is possible to “switch‐off” an entire data source for a given country–vaccine combination if it is deemed unfit for model‐fitting, and then utilize input data from other sources for coverage estimation. Making such decisions on a case‐by‐case basis, though arduous, could lead to better quality modeled estimates that reflect the peculiarities of individual countries. There is, however, a need to strike a balance between how much data are available for model‐fitting and the quality of modeled estimates that is desired. Additional data processing steps could also include expertly identifying and excluding any implausible outlying observations (e.g., some coverage estimates ≈1% included in the current analysis) that could bias the modeled estimates.

The modeling approaches outlined here and their implementations are subject to other limitations. The $\hat{R}$ metric used in our work, though a practical diagnostic tool, may fail to detect convergence in some cases such as when there are (i) divergent transitions in the NUTS sampler, (ii) heavy‐tailed posteriors or (iii) warnings in Stan despite low $\hat{R}$. It is therefore recommended to use this in combination with other diagnostics such as trace plots, rank plots and effective sample size, and using input parameter adjustments such as “adapt‐delta” to resolve any divergence issues, or even model reparametrization, etc. (Brooks and Gelman 1998; Papaspiliopoulos et al. 2007; Vehtari et al. 2021). Since the imcover package extends rstan, users have the flexibility to interact with the functions provided using standard rstan parameters for tuning and sampling. Currently, our methodology does not utilize any covariate information for coverage estimation. The inclusion of highly informative covariates such as access to a health facility, antenatal care attendance, and female literacy rates (see, e.g., Galles et al. 2021; Utazi et al. 2022) in the models can help improve the accuracy and precision of the estimates. It will be fairly straightforward to implement this, but we anticipate that it will greatly simplify the structures of some of the terms used to account for residual variation in models (1) and (2), or make these redundant. An effective model selection criterion will therefore be needed to determine the best model parameterization in this setting. Furthermore, our modeled estimates were produced with wide uncertainties in some cases, particularly where input data were scarce. The amount of uncertainty present in the modeled estimates could be further controlled by adjusting the priors on $\lambda^{\left(a\right)}$, $\lambda^{\left(o\right)}$ and $\lambda^{\left(s\right)}$ in the IDML model. The source‐specific term $\nu^{\left(k\right)}$ in model (1) can be allowed to vary by country to potentially capture variation between data sources peculiar to each country. It is possible to use different probability distributions in the IDML model to better account for the uncertainties in each data source, subject to the constraints in the available data. For example, since both the numerator and denominator estimates are available for reported admin data, these could be modeled with a binomial likelihood or a weighted Gaussian likelihood (using a function of the denominator as weights (see, e.g., Shand et al. 2018)) to better account for the binomial variation in the data, while modeling the official and survey estimates using Gaussian likelihoods. Also, for survey estimates, if the associated uncertainties are available, these can be included in the model using a weighted Gaussian likelihood or a Fay–Herriot type model (Fay and Herriot 1979). As noted previously, we could not account for these uncertainties in the current analysis as they were not included in the input data obtained for this work.

Future work will focus on extending the proposed methodology to subnational coverage estimation, which is highly relevant to current global health agenda (United Nations General Assembly 2015; World Health Organization 2020). While this will present additional challenges (see, e.g., Brown 2018), we anticipate that subnational data will have a richer spatial structure unlike country‐level data, which can be accounted for using conditional autoregressive priors (Lee 2011). Furthermore, our predictions for 2020 and 2021 – which represent a counterfactual non‐pandemic scenario – can be used to evaluate the impact of the Covid‐19 pandemic on routine immunization coverage. Lastly, we will extend our analyses to include other vaccines not used for model development.

In conclusion, our work holds a lot of promise as it adds to increased efforts to boost the quality of immunization coverage estimates available for global health policy and decision‐making.

## Conflicts of Interest

The authors declare no conflicts of interest. MCD‐H and MG‐D are staff members of WHO while PM worked for UNICEF at the time of this work. The authors alone are responsible for the views expressed in this article and they do not necessarily represent the views, decisions, or policies of the institutions with which they are affiliated.

## Open Research Badges

This article has earned an Open Data badge for making publicly available the digitally‐shareable data necessary to reproduce the reported results. The data is available in the Supporting Information section.

This article has earned an open data badge “**Reproducible Research**” for making publicly available the code necessary to reproduce the reported results. The results reported in this article were reproduced partially due to incomplete intermediate results.

## Supporting information


**Supporting File 1:** bimj70189‐sup‐0001‐SuppMat.pdf.

**Supporting File 2:** bimj70189‐sup‐0002‐Data.zip.

## Data Availability

The data used for this work are publicly available from the WHO data portal: https://immunizationdata.who.int. The software to run the analysis is available from GitHub: https://wpgp.github.io/imcover/.
