# Supplementary material for: Bayesian Hierarchical Modeling Approaches for Combining Information From Multiple Data Sources to Produce Annual Estimates of National Immunization Coverage
Source: Biom J. 2026 Oct 6;68(5):e70189. doi: 10.1002/bimj.70189 (PMC13652340; doi:10.1002/bimj.70189)
Supplement: Supplementary file 1 [file BIMJ-68-e70189-s001.pdf]

Bayesian hierarchical modelling approaches for combining information from multiple data sources to produce annual estimates of national immunization coverage

Supplementary information

C. Edson Utazi, Warren C. Jochem, M. Carolina Danovaro-Holliday, Padraic Murphy, Sujit K. Sahu, Marta Gacic-Dobo and Andrew J. Tatem

This document accompanies the main paper. It contains additional information, including additional tables and figures referenced in the paper.

1. Recall bias adjustment and processing of survey data

For survey data, we implemented an additional data cleaning step to ensure consistency in the entries in each column, particularly where these were character variables. Each coverage survey estimate was then linked to a 'birth cohort year' which we used as the reference year for the estimate in this work. The birth cohort year was determined using the period of data collection and the age of the birth cohort that the survey estimate relates to, as in WUENIC methodology.

Similar to WUENIC approach, we applied a recall-bias adjustment to DTP3 and PCV3 survey estimates. Estimates based on vaccination cards only or vaccination cards and recall were used for the adjustment. In the pre-cleaned input data file, these estimates were labelled as: "Card" and "Card or History", respectively, in the column for evidence of vaccination. For country-vaccine-year combinations with multiple estimates labelled as "crude" or "valid", the "valid" estimates were retained in the analysis as these were considered more accurate. The formula used for the adjustment is:

$$\mathrm{VD3}_{(\mathrm{card+history})} = \mathrm{VD3}_{(\mathrm{card\ only})} \times \frac{\mathrm{VD1}_{(\mathrm{card+history})}}{\mathrm{VD1}_{(\mathrm{card\ only})}} \tag{1}$$

where VD3 denotes the coverage of third dose of DTP or PCV vaccine and VD1, the first dose. We note that for each vaccine, the adjustment was applied only when all the data needed to compute equation (1) were available. After the adjustment, the original "Card or History" survey estimates of DTP3 and PCV3 were replaced with corresponding bias-adjusted estimates for further processing.

For a given vaccine, country and year, if one survey estimate was available, it was accepted if the sample size was greater than 300 or if the estimate was labelled 'valid'. Otherwise, the estimate was not accepted. Where multiple estimates were

available for the same vaccine, country and year, "Card or History" estimates were prioritized over "Card" only estimates, and either of these were accepted if the corresponding sample size was greater than 300 or if the evidence of vaccination was based on valid doses. We note that for DTP3 and PCV3, only the bias-adjusted estimates were considered when available. When multiple estimates were available from the preceding step (perhaps from different surveys or the same survey) for the same vaccine, country and year, the estimate with the largest sample size was accepted. If the sample sizes were missing, the first valid estimate or the first estimate available was chosen, in the given sequence. The resulting survey estimates were used in the rest of the analyses.

2. Software

In order to support the reproducibility and replication of the immunization coverage modelling methods described in this report, we developed a set of tools in the R programming language (1). The `imcover` package provides functions for assembling the common sources of immunization coverage and for fitting the base single likelihood (BSL) and irregular data multiple likelihood (IDML) models described in Section 3 using full Bayesian inference with Stan (2). The latest version of imcover can be installed from GitHub by typing the command within the R console:

```
remotes::install_github('wpgp/imcover', force = T).
```

In order to properly install the package, a C++ compiler is required. Internally, `imcover` relies on Stan code which is translated into C++ and compiled, allowing for faster computations. On a Windows PC, the Rtools program provides the necessary compiler. This is available from `https://cran.r-project.org/bin/windows/Rtools/` for R version 4.0 or later. On Mac OS X, users should follow the instructions to install XCode. Users are advised to check the Stan installation guide for further information on the necessary system set-up and compilers (`https://github.com/stan-dev/rstan/wiki/RStan-Getting-Started`).

A typical workflow using imcover to produce national- and regional-level immunization coverage estimates is illustrated in Figure 1. The user should first download the time series of reported coverage data, which may come from multiple sources (i. e., administrative, official, and survey estimates). Second, these datasets are processed in several steps to filter, clean duplicate records and correct possible reporting biases and then assemble a single dataset. This stage creates datasets of format `ic.df` within R. This format is an extension of the common data frame and enables some of the specialised processing steps by `imcover`. Third, the model is fitted against the assembled coverage dataset. The user has the option at this stage to specify additional parameters and prior choices for the statistical model. Fourth, after the model has been fitted, a model object is returned. This object's class extends the model objects from `rstan` from which parameter estimates and other results can be extracted in R. In the fifth stage, post-processing is done on the model results. These functions provide support to produce summaries of the

estimates, predictions forward in time, standardized visualizations, and population-weighted regional aggregations of immunization coverage estimates. The details of this workflow are illustrated below with a worked example of coverage data for the WHO AFR region. Further information on the R package can be found within the documentation, including a long-form vignette, see $help(imcover)$.

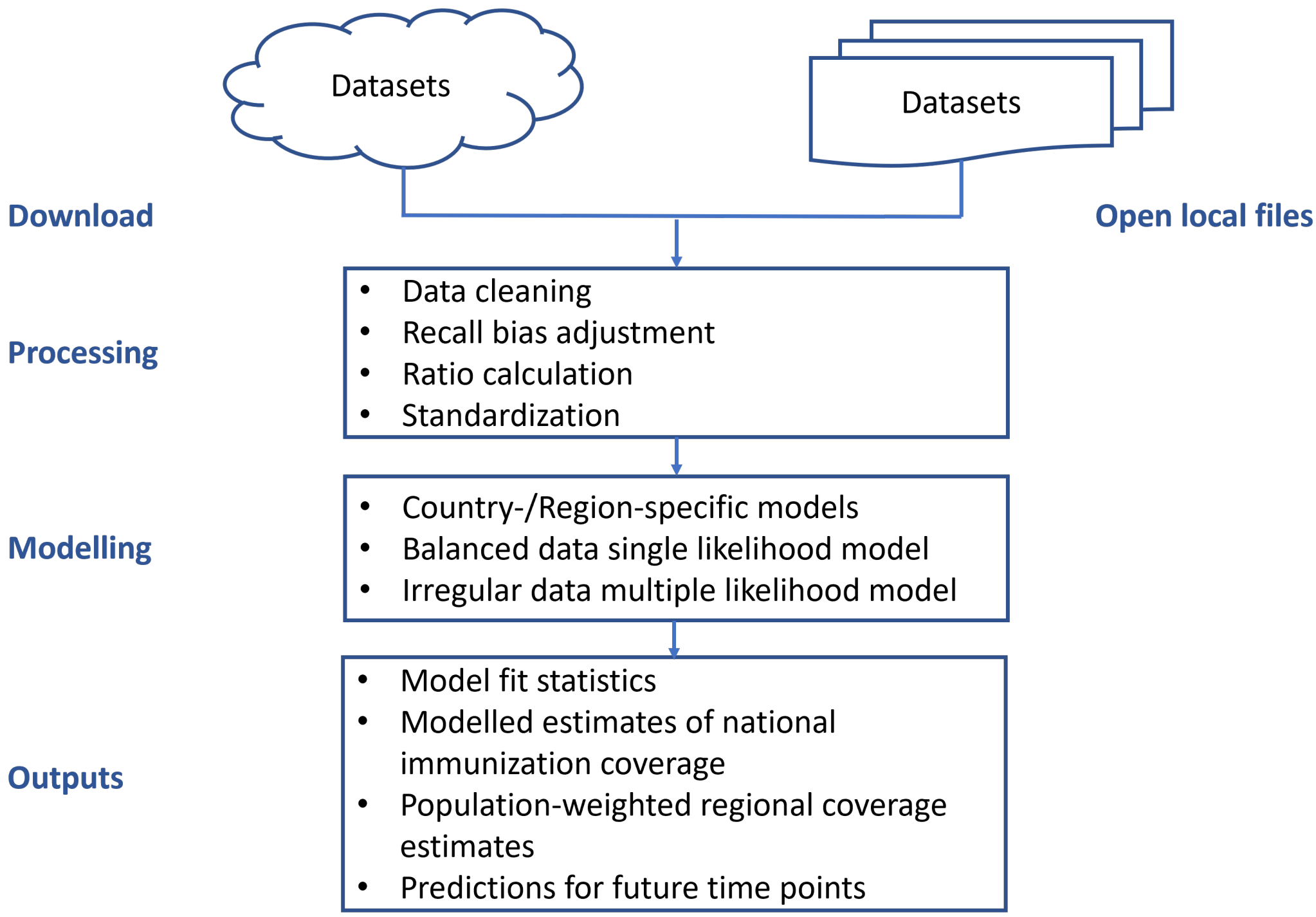

Supplementary Figure 1: Overview of steps supported by the `imcover` package.

Workflow example

In the following sections we provide a worked example to produce time series of modelled estimates of immunization coverage using the model-based approach implemented in R using `imcover`. After loading the package, we first obtain the data from the WHO Immunization Data Portal (`https://immunizationdata.who.int`). An internet connection is required for this step.

```
# load the package within the R environment
library(imcover)

# download administrative and official records
cov <- download_coverage()

```

```
# download survey records
svy <- download_survey()
```

Data downloading is handled by two functions: `download_coverage` and `download_survey`. These handle administrative/official estimates or survey datasets, respectively. By default, the downloaded files are stored as temporary documents in the user's R temporary directory; however, the functions provide the option for users to save the downloaded files to a user-specified location and load them later from a local file path. In this way, a user can also load their own source of immunization coverage data and process it into a standardized format for modeling.

Data processing and formatting

As part of the download function, a series of checks and cleaning steps are applied by default. The goal of these checks is to identify the core attributes in the input data necessary to construct an immunization coverage dataset. Specifically, these attributes include a country, time, vaccine identifier, and percent of the population covered by that vaccine. In the absence of a reported coverage percentage, the number of doses administered and target population can be used to estimate coverage. Identifying the core attributes allows the user to harmonize multiple source files into a unified data format for modelling. Administrative and official coverage estimates are processed similarly. Within `download_survey`, the household survey datasets require some more specialized processing. For instance, multi-dose vaccine reports can have reporting and recall biases. Note that all the processing steps can be carried out using separate functions available in `imcover` if advanced users want more control over pre-processing.

Within the `imcover` package, processed coverage datasets are stored in `ic.df` format, or an "immunization coverage data frame". This format extends the common data frame object of R where observations are rows and attributes are stored in columns. `ic.df` objects support all standard methods and ways of working with data frames in R. This includes selecting records and columns by indices or column names, merging data frames, appending records, renaming, etc. The advantage of the `ic.df` format over a standard data frame is that it includes information to identify the columns containing core coverage information as well as notes on data pre-processing that has been done. These allow users to combine disparate sources of information on immunization coverage into a harmonised analysis dataset without having to adjust for missing or differently named columns.

```
# note the type of object created
class(cov)
#> [1] "ic.df"    "data.frame"
```

The data files available from the WHO website require some additional cleaning before analysis. Notice that the data objects created by `imcover` can work with all standard R commands.

```
# Further data cleaning of immunization records
# drop some record categories (PAB, HPV, WUENIC)
cov <- cov[cov$coverage_category %in% c("ADMIN", "OFFICIAL"), ]
cov$coverage_category <- tolower(cov$coverage_category)  # clean-up

# create a combined dataset
dat <- rbind(cov, svy)

# remove records with missing coverage values
dat <- dat[!is.na(dat$coverage), ]

# mismatch in vaccine names between coverage and survey datasets
dat[dat$antigen == 'DTPCV1', 'antigen'] <- 'DTP1'
dat[dat$antigen == 'DTPCV2', 'antigen'] <- 'DTP2'
dat[dat$antigen == 'DTPCV3', 'antigen'] <- 'DTP3'

# subset records
dat <- ic_filter(dat,
                 vaccine = c("DTP1", "DTP3", "MCV1", "MCV2", "PCV3"),
                 time = 2000:2020)
```

In preparation for the statistical modelling we carry out several additional pre-processing steps. Firstly, some records observe inconsistencies in the levels of coverage between multi-dose vaccines. To maintain consistency, where coverage of later doses cannot exceed earlier doses, we model the ratio between first and third dose. In this example, we only adjust DTP1 and DTP3, but other multi-dose vaccines could be processed in a similar manner.

```
# adjustment - use ratio for DTP3
dat <- ic_ratio(dat, numerator = 'DTP3', denominator = 'DTP1')
```

The `ic.df` object will now store a note that this processing step has been carried out so that the ratio is correctly back-transformed and that coverage estimates and predictions are adjusted appropriately.

Secondly, we need to force coverage estimates to lie between 0% and 100% so that we can model the data with a logit transformation.

```
# maintain coverage between 0-100%
dat <- ic_adjust(dat, coverage_adj = TRUE)
```

Fitting models with `imcover`

The core of `imcover` is the functionality to fit a Bayesian statistical model of multiple time series. A discussed in Section 3, this includes a BSL and an IDML model.

The sources of coverage data (in this example administrative, official and surveys) are taken as multiple, partial estimates of the true, unobserved immunization coverage in a country. A Bayesian estimation approach allows us to incorporate these multiple datasets, place prior beliefs on which sources are more reliable, share information between countries, and to quantify uncertainty in our estimate of the true immunization coverage.

`imcover` provides an interface to Stan (2) for statistical computation. This means that, in addition to `imcover`, many of the R programming language tools for assessing model performance and visualizing results from `rstan` will work for `imcover` results.

For this example we subset the records to the AFR region based on a regional identifier and the most recent years.

```
# enable parallel processing in rstan
options(mc.cores = parallel::detectCores())

# Fit model to a single region of data
fit <- ic_fit(dat[dat$region == 'AFRO', ],
              chains = 4,
              iter = 2000,
              warmup = 1000)
```

The previous command uses the IDML model, and `ic_fit_single` can be used to fit a BSL model to the same data. Other options to these fitting functions include, `prior_sigma` which can be used to set the scale parameter for the truncated Cauchy priors on σ and `upper_sigma` which allows users to set an upper bound on the scale parameter for each data source.

Model outputs

After fitting the model, we can work with the parameter estimates and estimates of coverage. As noted previously, the fitted models returned by `ic_fit` are `rstan`-class objects and are compatible with many other tools, such as `bayesplot`. `imcover` wraps these objects into a new class, `ic.fit`, which, along with the fitted model object, contains additional information on the model type and data pre-processing steps to facilitate most common analyses of immunization coverage.

For example, we can easily make graphs of coverage estimates using a generic `plot` function.

```
# Plot of coverage
plot(ic_filter(fit, vaccine = 'DTP1',
               country = c('BWA', 'SLE', 'GHA', 'NGA')),
    ncol = 2)

# Plot of coverage overlaid with WUENIC estimates
plot(ic_filter(fit, vaccine = 'DTP1',
               country = c('BWA', 'SLE', 'GHA', 'NGA')),
    ncol = 2,
```

```
    wuenic = TRUE)
```

The main output of interest from `imcover` is a table of coverage estimates for each country, vaccine, and time point, along with uncertainty around each estimate. We can extract these data as a table, save them to a data frame, or write them out to a file for use in a report.

```
# Extracting coverage estimates to a data frame
ic <- ic_coverage(fit,
                  stat = "quantile", # customise the summary
    function
                  probs = c(0.025, 0.5, 0.975))
```

Population-weighted regional aggregations of immunization coverage can also be calculated.

```
ic_regional(fit,
            stat = c("mean", "quantile"),
            probs = c(0.025, 0.5, 0.975)
```

One we have a fitted model for a specific time period, we can also use it to predict coverage to time point in the near future.

```
# Predict for future time points (3 years post 2018)
fit1 <- predict(fit1, t = 3) # update icfit to include '
    prediction' info

# Update the graph to include fitted estimates, observed data,
     and predictions
plot(ic_filter(fit, vaccine = 'DTP1',
     country = c('BWA', 'SLE', 'GHA', 'NGA')),
     ncol = 2,
     predition = TRUE)
```

This section has introduced the core functionality of the `imcover` package and its use in modelling national time series of immunization coverage. Providing a coordinated toolset to download, process, and model immunization data should allow for consistent analyses whose methods are transparent and replicable.

3. Supplementary tables and figures

Supplementary Table 1: Data sources, descriptions and associated biases

Data Source	Description	Nature of Biases
Reported administrative coverage data (admin)	Estimates of immunization coverage which countries report annually to WHO and UNICEF through the Joint Reporting Form for Immunization Data (JRF) (3). Administrative estimates of coverage are usually obtained by dividing the number of doses administered to a target population by the total estimated number of people in the target population.	Incomplete reporting (especially by the private sector); inaccurate reporting or aggregation of numerators; inaccurate population denominator estimates; inflation of numerator estimates due to vaccination of individuals outside the target population; etc.
Country reported official estimates (official)	These are coverage data which represent the country's own assessment of the most likely coverage. These data may have been reviewed by countries against other in-country datasets, or corrected for private sector under-reporting or by a factor based on data quality self-assessments, etc. In some cases, official estimates are the same as administrative estimates, but in other cases, are based on surveys or 'corrections' for known inaccuracies in the admin estimates, e.g., incomplete reporting, as described above.	Often based on an ad hoc adjustment of admin data by countries; but it can also be based on survey data when there is no admin system or when a survey is believed to be more accurate. Surveys have their own biases (see below).
Household surveys of vaccination coverage (survey)	These are estimates of immunization coverage obtained from nationally representative household surveys implemented in WHO Member States, mainly from (i) the Expanded Programme on Immunization (EPI) cluster surveys; (ii) the Demographic and Health Surveys (DHS) and (iii) the Multiple Indicator Cluster Surveys (MICS), but also from other designs (e.g., the United States National Immunization Coverage Survey).	Small sample sizes in some cases; inaccurate or outdated sample frame could result in underrepresentation of certain populations; difficulty in sampling populations living in security-compromised areas; accuracy of estimates depends on survey methodology (e.g., supervision, quality control) and availability of home based records of vaccination; data are irregularly available; etc (4; 5).

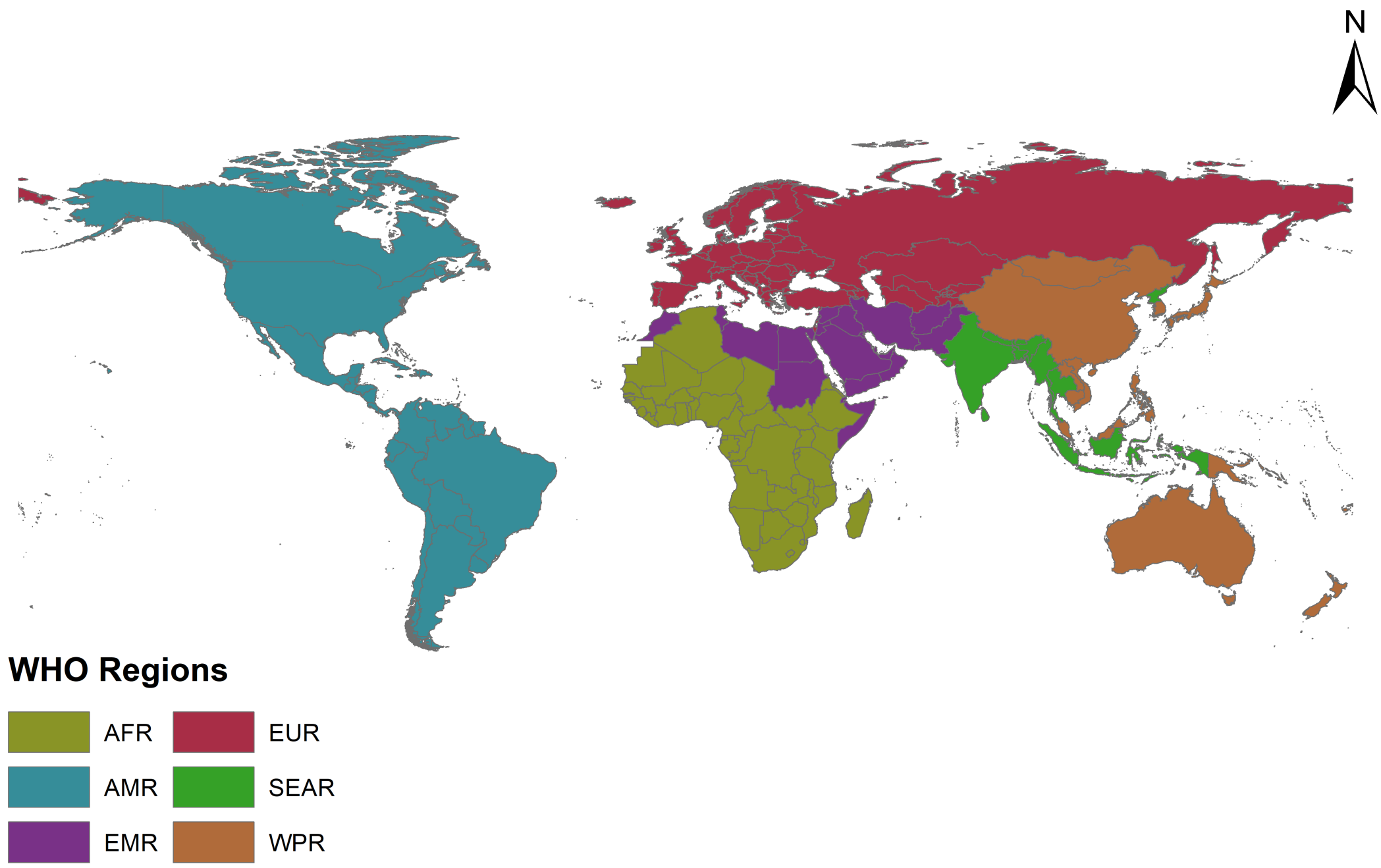

Supplementary Figure 2: WHO Member States and regions.

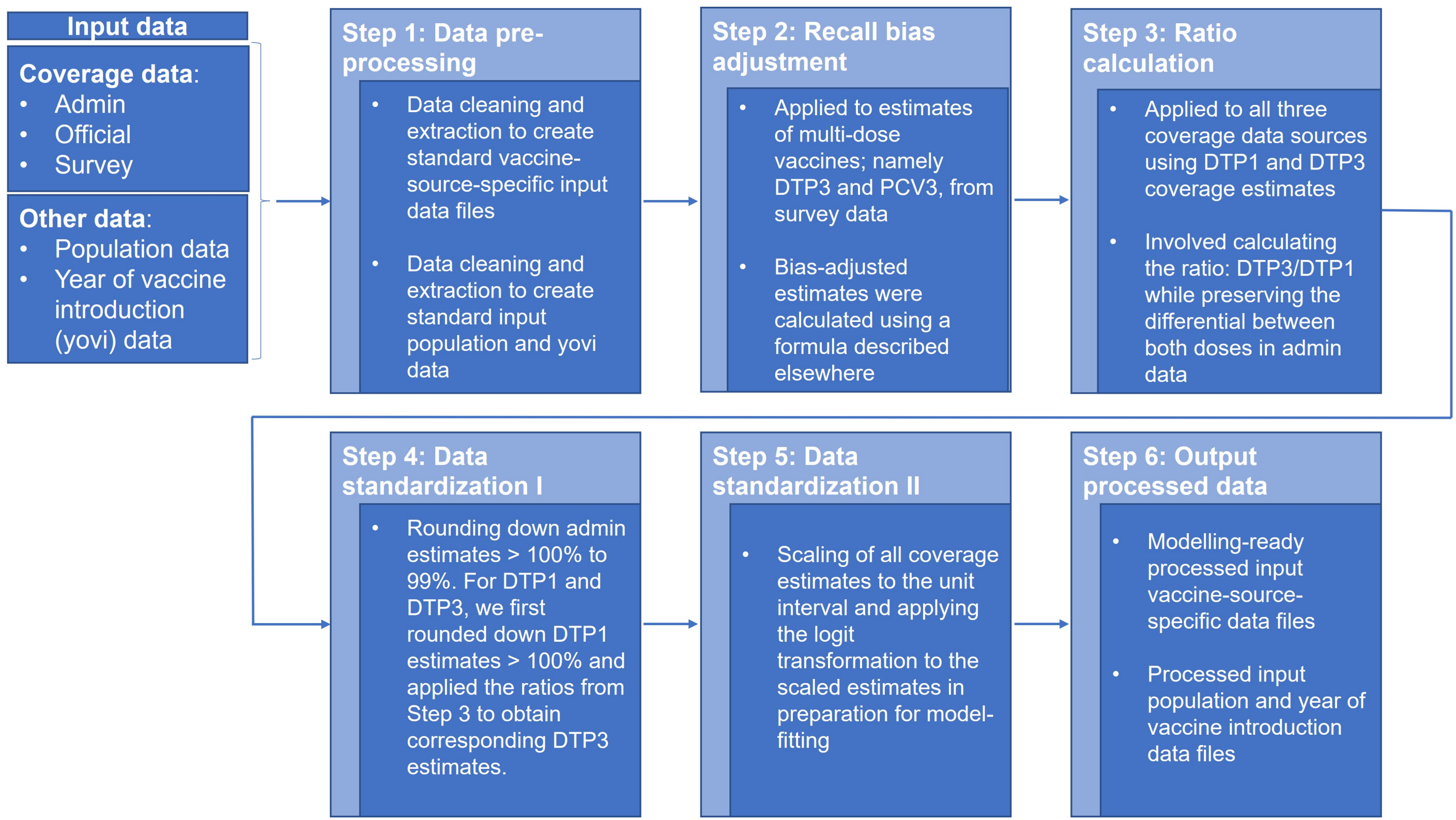

Supplementary Figure 3: A flow diagram illustrating the data processing steps.

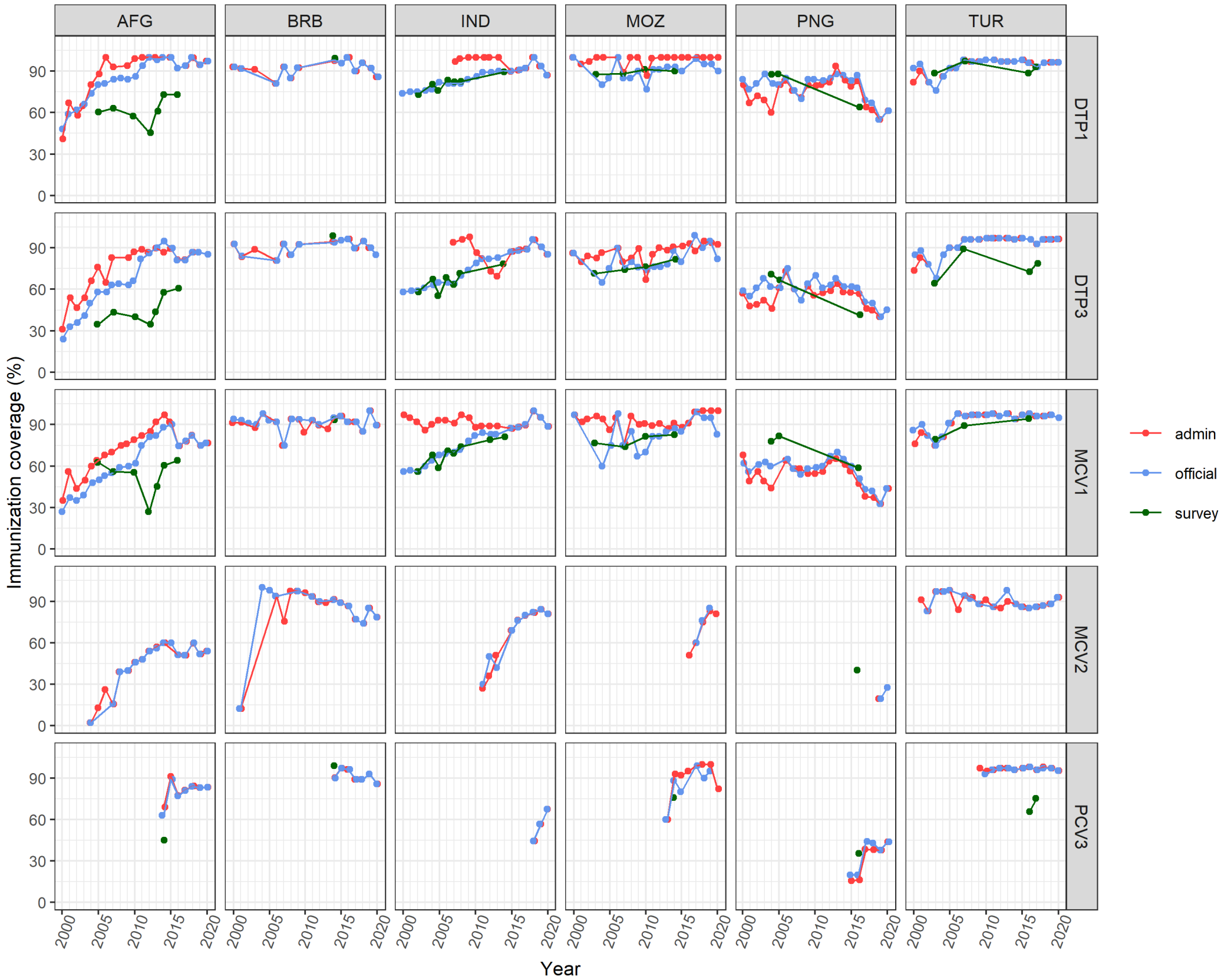

Supplementary Figure 4: Time series plots of the processed input administrative, official and survey data for some example countries.

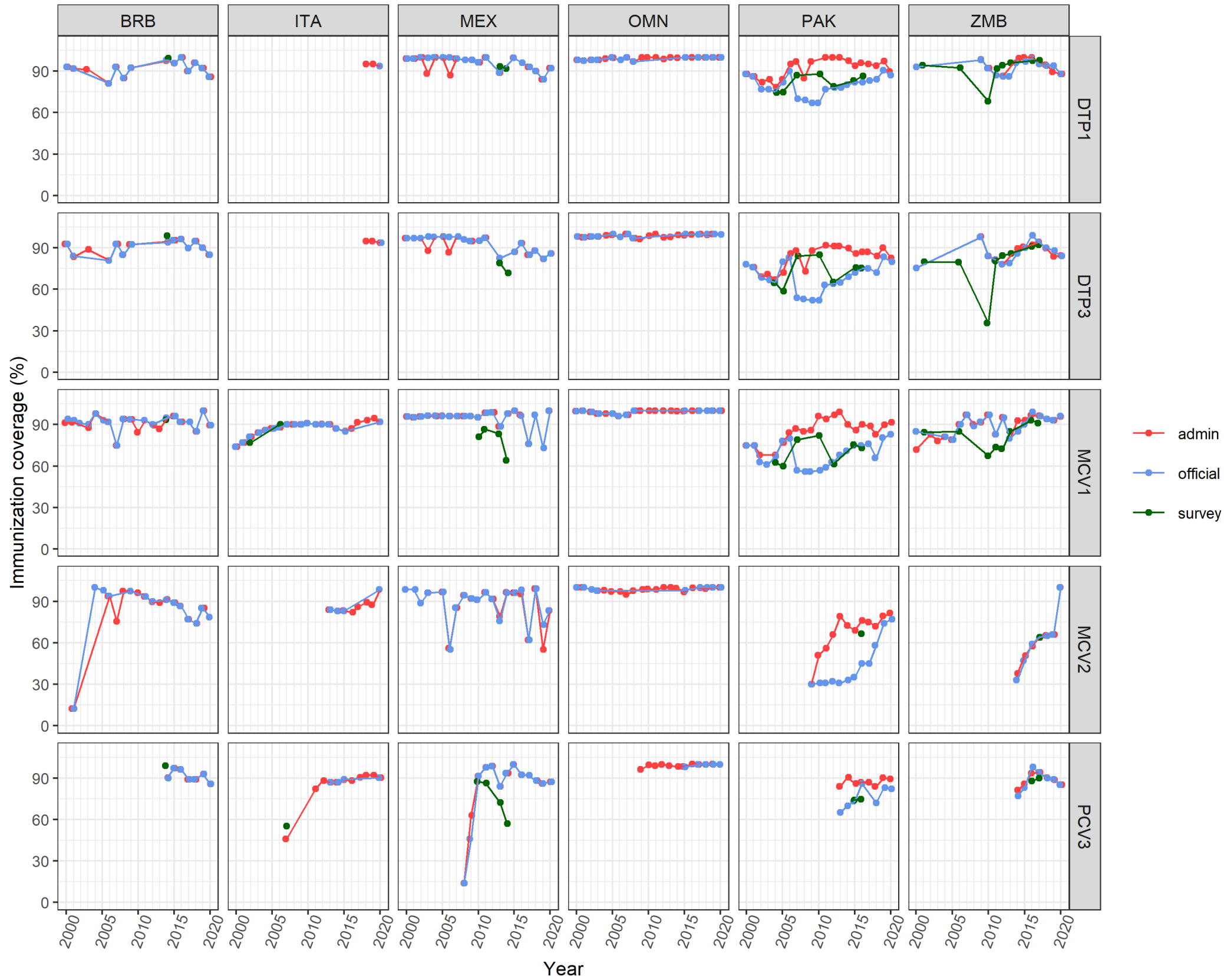

Supplementary Figure 5: Time series plots of the processed input administrative, official and survey data for some example countries.

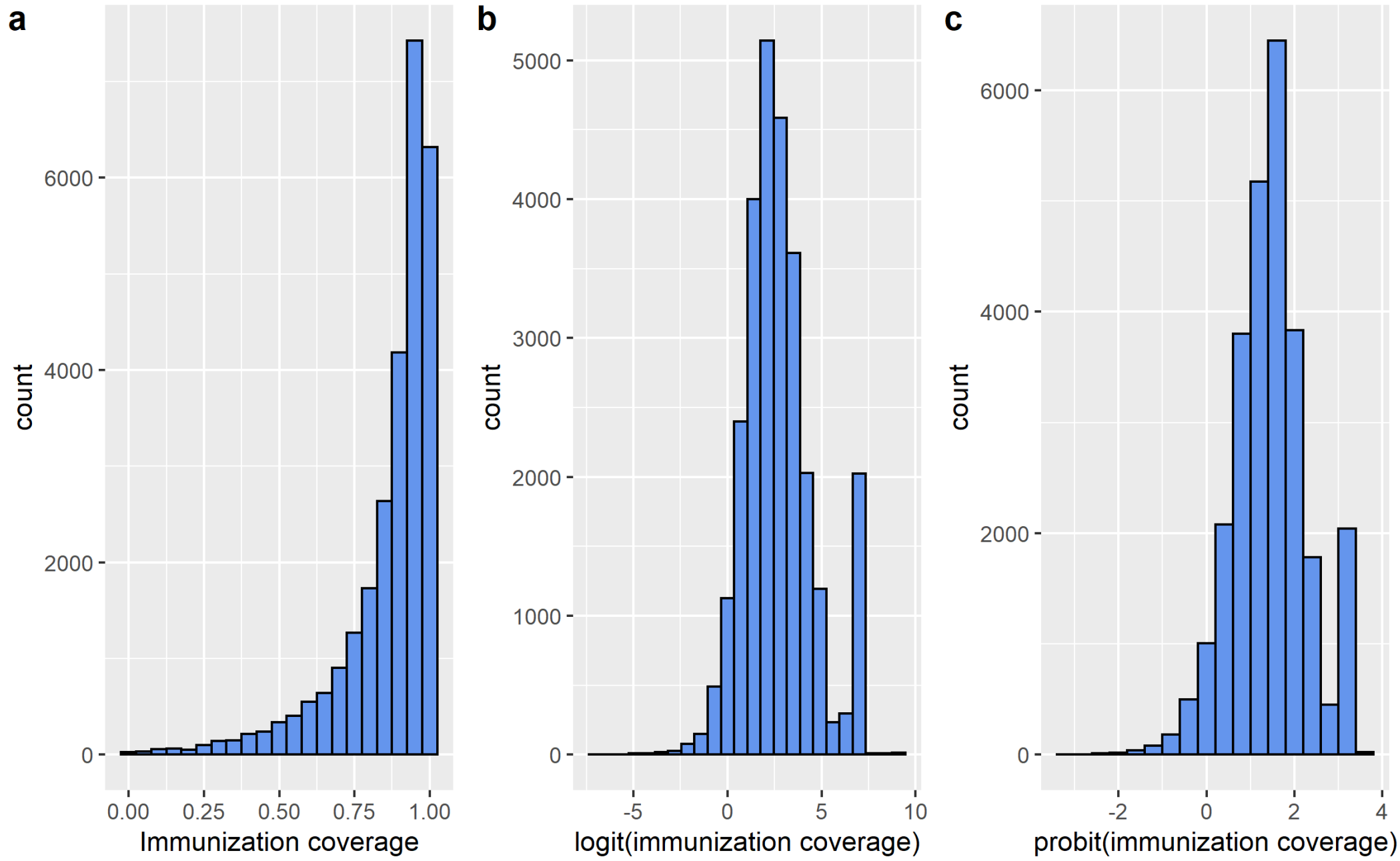

Supplementary Figure 6: Distributions of (a) original, (b) logit-transformed and (c) probit-transformed processed pooled national immunization coverage data.

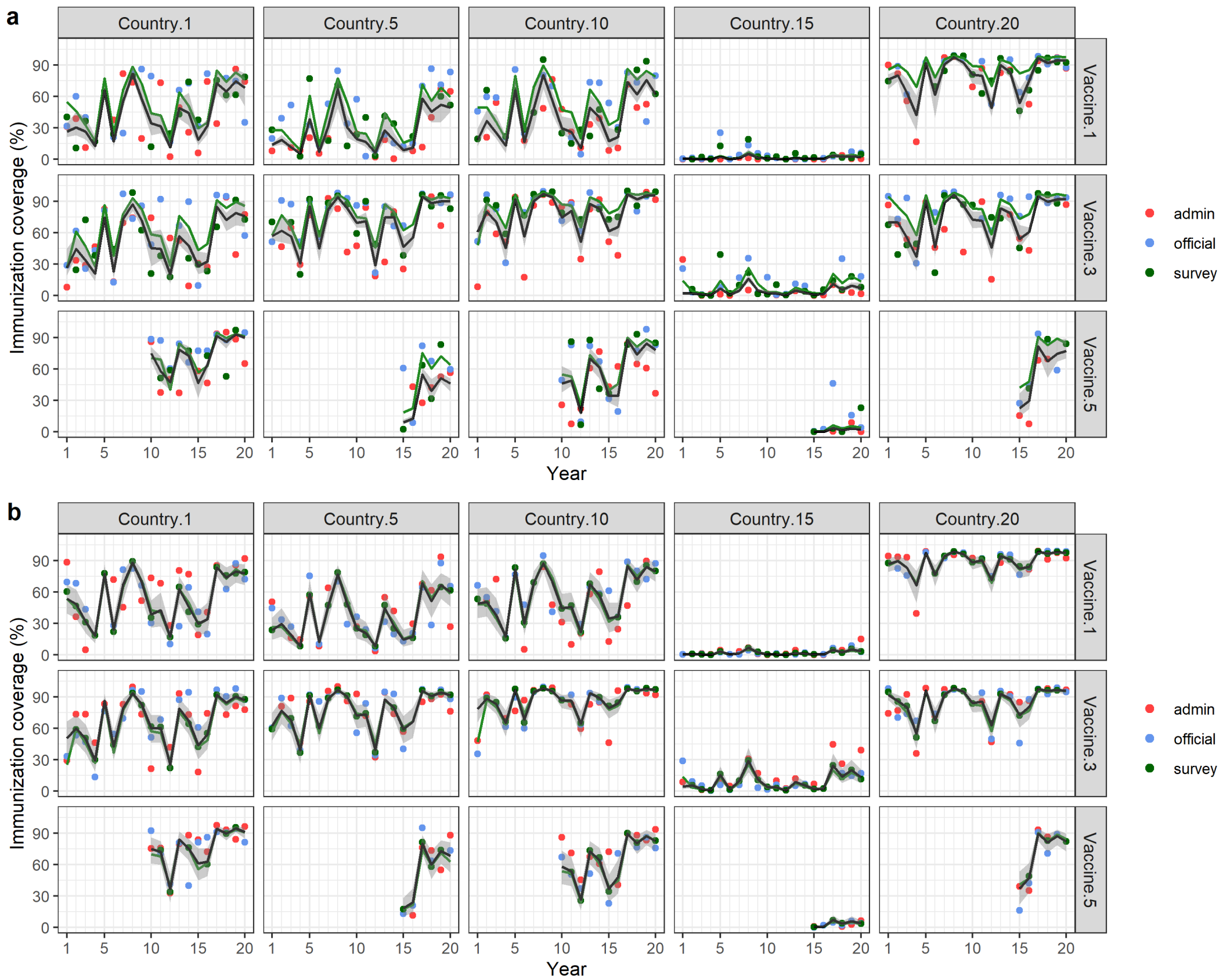

Supplementary Figure 7: Plots of simulated and modelled estimates of national immunization coverage for five countries and three vaccines using (a) the base single likelihood (BSL) model and (b) the irregular data multiple likelihood (IDML) model. The solid black lines and grey-shaded areas are the estimates of p_{ijt} and corresponding 95% credible intervals, and the green lines are the true values. The data were generated under scenario 1 described in Section 5.

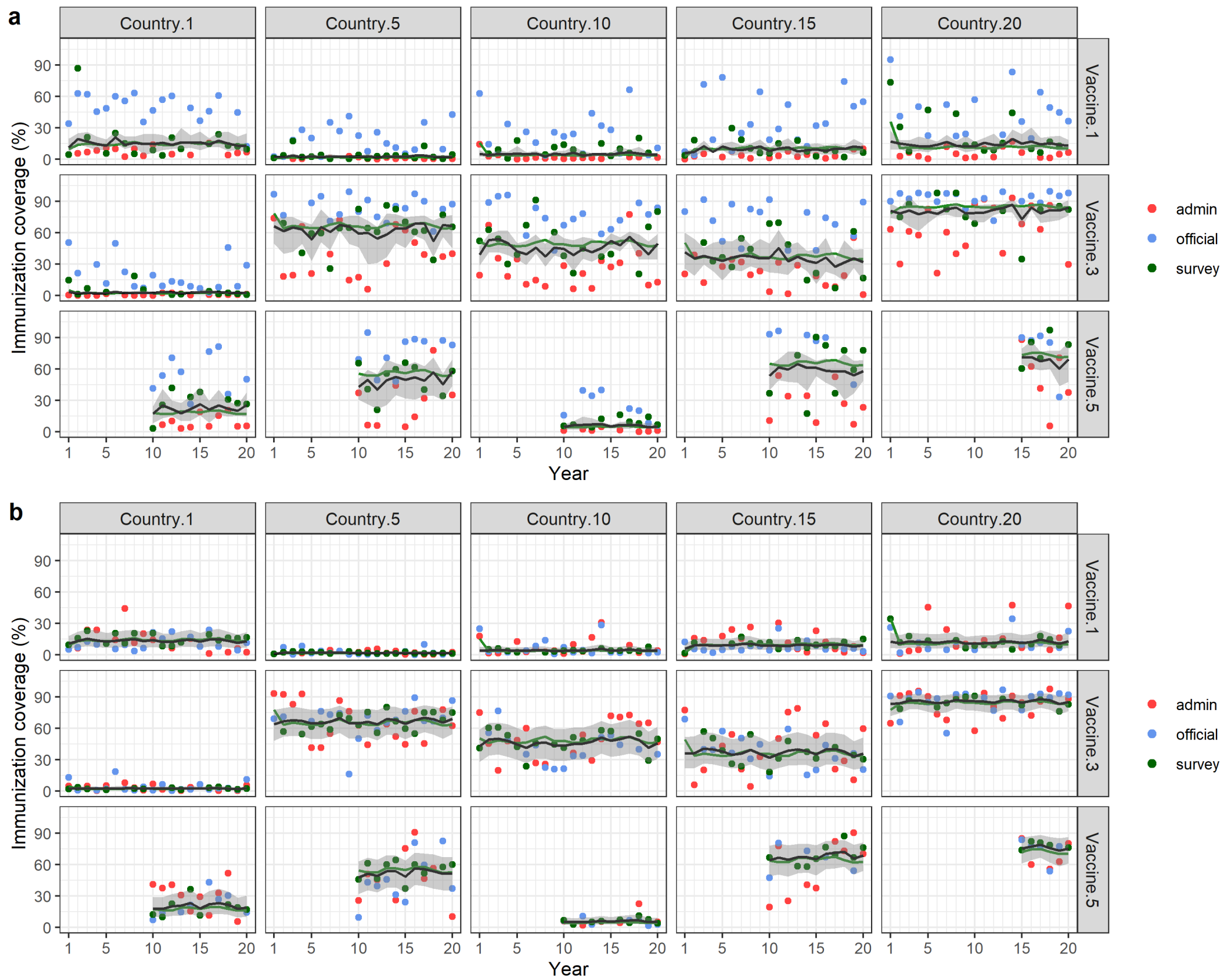

Supplementary Figure 8: Plots of simulated and modelled estimates of national immunization coverage for five countries and three vaccines using (a) the base single likelihood (BSL) model and (b) the irregular data multiple likelihood (IDML) model. The solid black lines and grey-shaded areas are the estimates of p_{ijt} and corresponding 95% credible intervals, and the green lines are the true values. The data were generated under scenario 1 described in Section 5, but with the variance parameter of the temporal random effect γ_t (i.e., σ_γ^2) set equal to 0.01 to induce less variability in p_{ijt}.

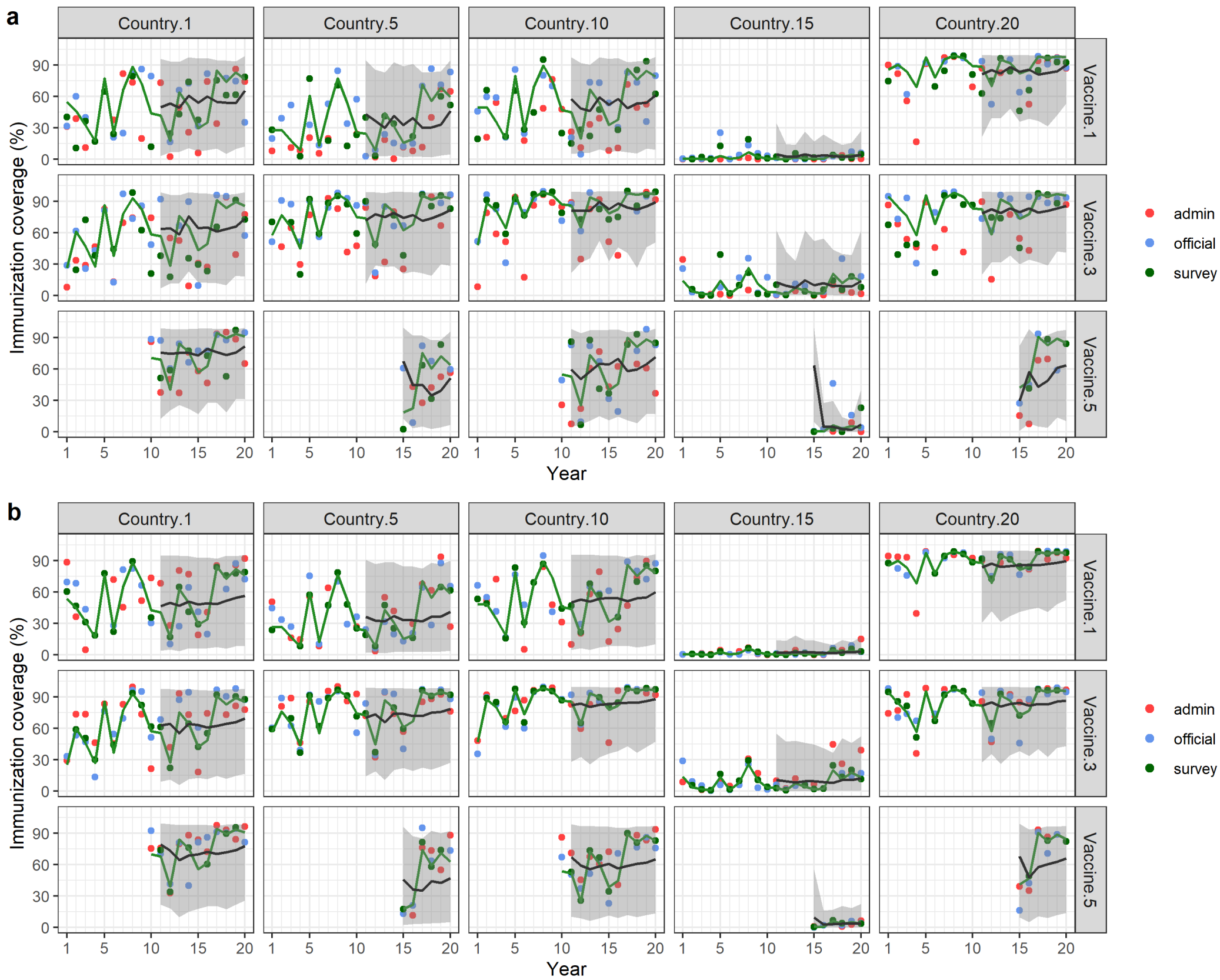

Supplementary Figure 9: Plots of the simulated data and one-step-ahead predicted estimates of national immunization coverage for five countries and three vaccines using (a) the base single likelihood (BSL) model and (b) the irregular data multiple likelihood (IDML) model. The solid black lines and grey-shaded areas are the one-step-ahead predicted estimates of p_{ijt} and corresponding 95% credible intervals, and the green lines are the true values. The data were generated under scenario 1 described in Section 5. We set the starting point for the one-step-ahead predictions at t = 11, meaning that we used the first 10 observations as base years for model-fitting and then obtained predictions for the remaining ten time points: $t = 11, ..., 20$.

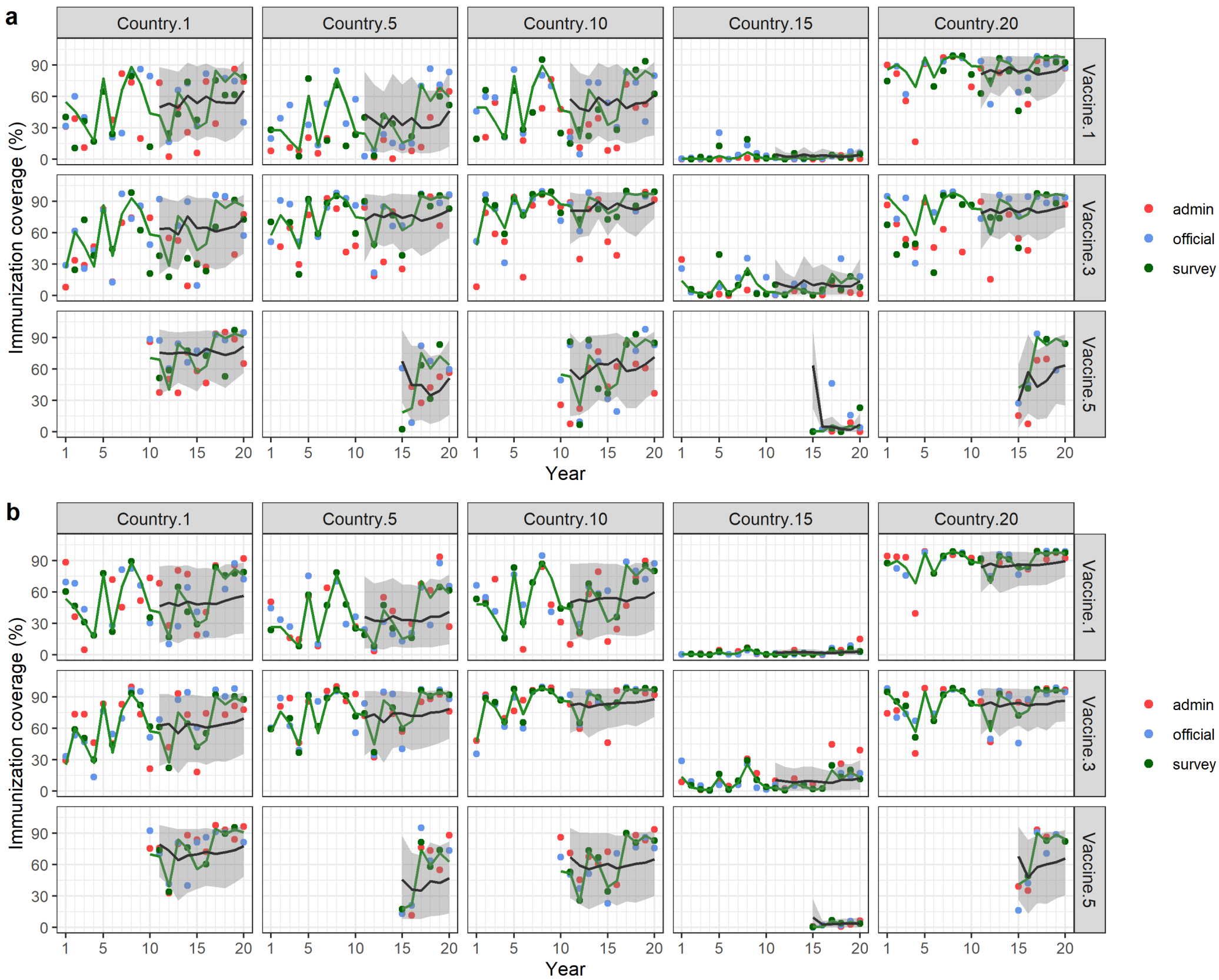

Supplementary Figure 10: Plots of the simulated data and one-step-ahead predicted estimates of national immunization coverage for five countries and three vaccines using (a) the base single likelihood (BSL) model and (b) the irregular data multiple likelihood (IDML) model. The solid black lines and grey-shaded areas are the one-step-ahead predicted estimates of p_{ijt} and corresponding 80% credible intervals, and the green lines are the true values. The data were generated under scenario 1 described in Section 5. We set the starting point for the one-step-ahead predictions at t = 11, meaning that we used the first 10 observations as base years for model-fitting and then obtained predictions for the remaining ten time points: $t = 11, ..., 20$.

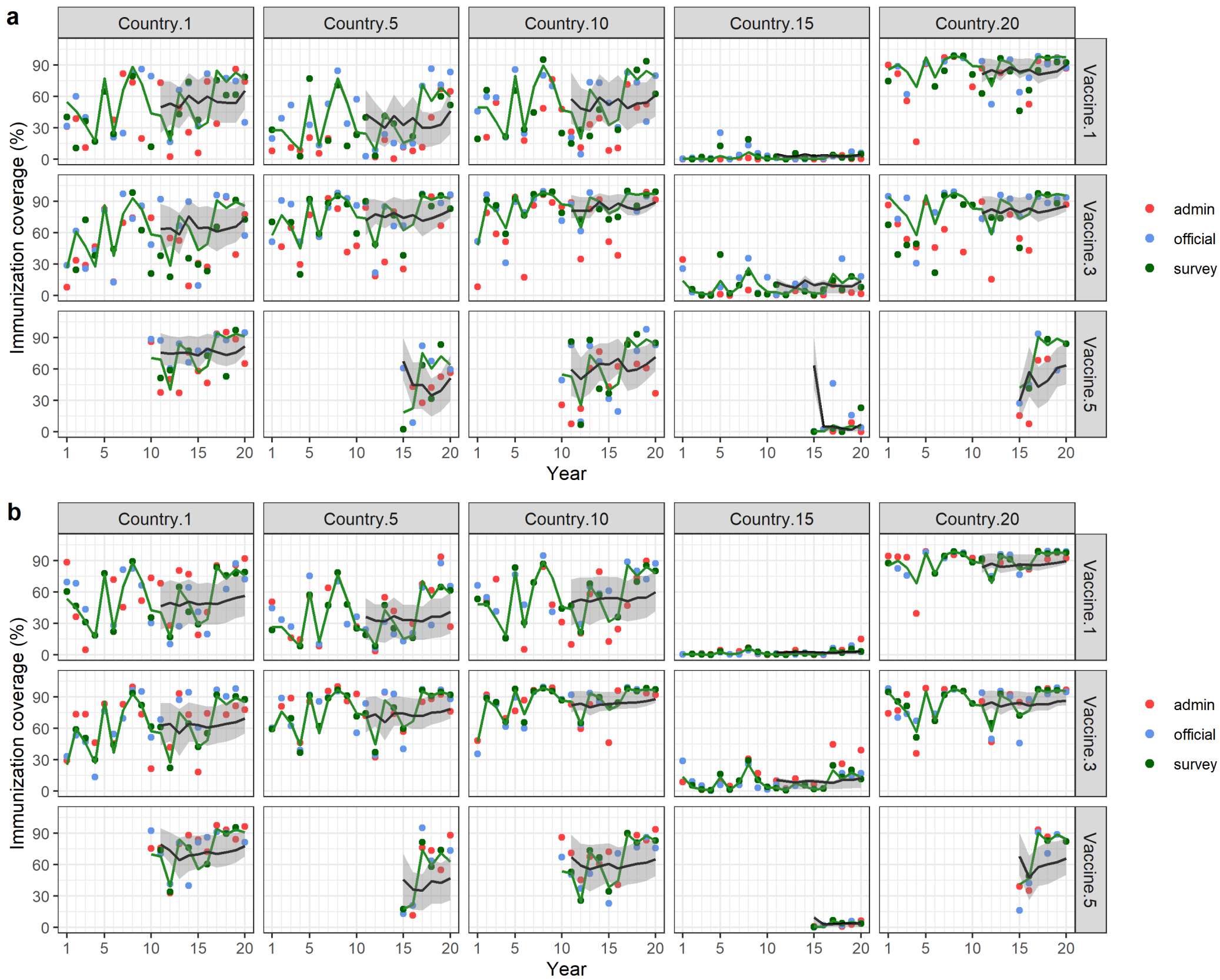

Supplementary Figure 11: Plots of the simulated data and one-step-ahead predicted estimates of national immunization coverage for five countries and three vaccines using (a) the base single likelihood (BSL) model and (b) the irregular data multiple likelihood (IDML) model. The solid black lines and grey-shaded areas are the one-step-ahead predicted estimates of p_{ijt} and corresponding 50% credible intervals, and the green lines are the true values. The data were generated under scenario 1 described in Section 5. We set the starting point for the one-step-ahead predictions at t = 11, meaning that we used the first 10 observations as base years for model-fitting and then obtained predictions for the remaining ten time points: $t = 11, ..., 20$.

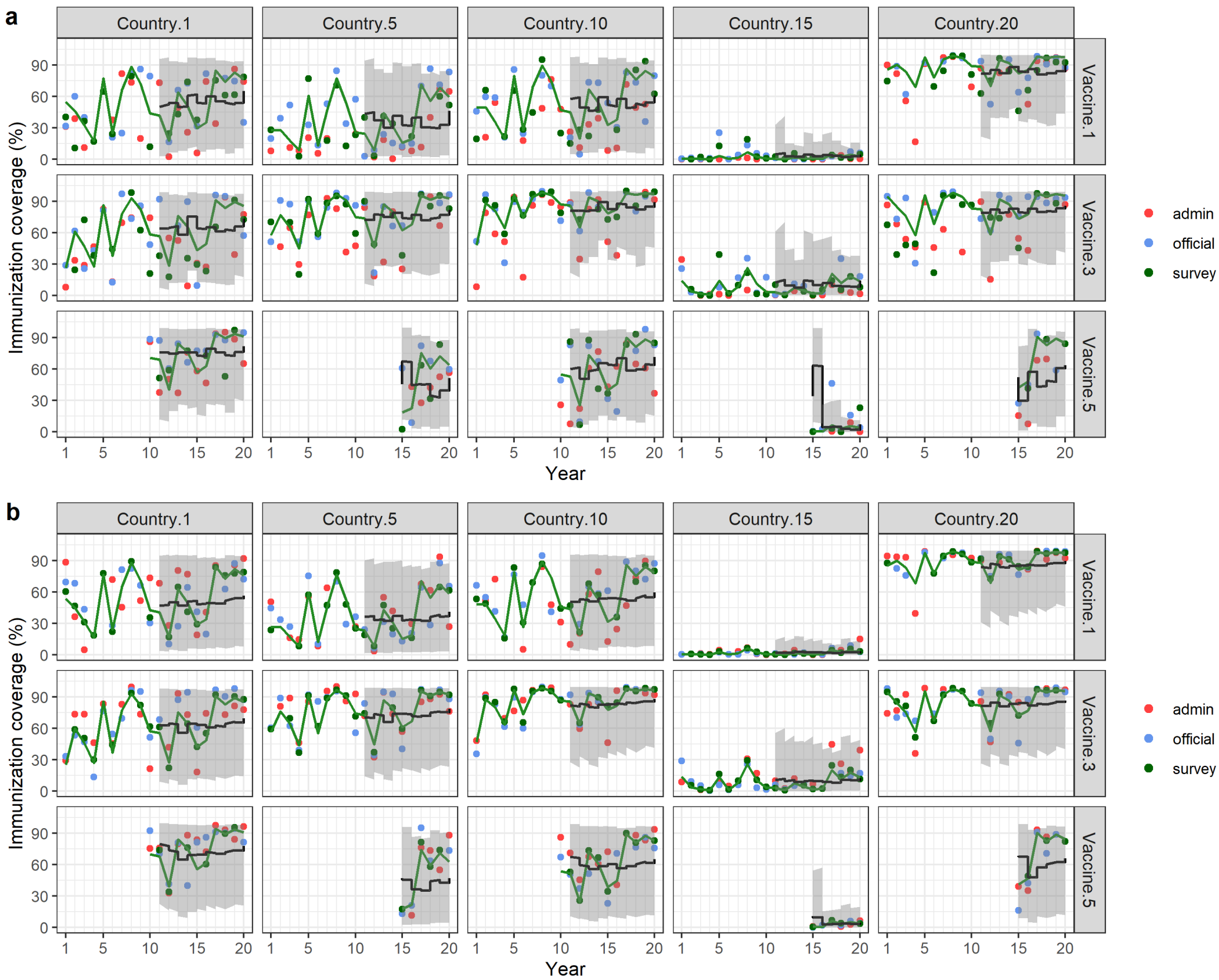

Supplementary Figure 12: Plots of the simulated data and two-step-ahead predicted estimates of national immunization coverage for five countries and three vaccines using (a) the base single likelihood (BSL) model and (b) the irregular data multiple likelihood (IDML) model. The solid black lines and grey-shaded areas are the one-step-ahead predicted estimates of p_{ijt} corresponding 95% credible intervals, and the green lines are the true values. The data were generated under scenario 1 described in Section 5. We set the starting point for the one-step-ahead predictions at t = 11, meaning that we used the first 10 observations as base years for model-fitting and then obtained predictions for the remaining ten time points: $t = 11, ..., 20$.

Supplementary Table 2: Posterior estimates of the parameters of the multiple likelihood model for AMR region

Parameter	Mean	Std. dev.	2.5%	50%	97.5%
$\hat{\lambda}^{(a)}$	0.501	0.286	−0.074	0.509	1.056
$\hat{\lambda}^{(o)}$	0.502	0.286	−0.074	0.510	1.057
$\hat{\lambda}^{(s)}$	−0.629	0.285	−1.203	−0.620	−0.080
$\hat{\sigma}_1$	0.422	0.011	0.401	0.421	0.444
$\hat{\sigma}_2$	0.412	0.011	0.391	0.412	0.434
$\hat{\sigma}_3$	0.400	1.9×10^{-4}	0.399	0.400	0.400
$\hat{\sigma}_\beta$	0.486	0.277	0.029	0.497	1.021
$\hat{\sigma}_\alpha$	3.027	2.069	0.560	2.656	7.733
$\hat{\rho}_\gamma$	0.147	0.507	−0.801	0.163	0.954
$\hat{\sigma}_\gamma$	0.107	0.062	0.006	0.104	0.238
$\hat{\rho}_\phi$	0.950	0.025	0.888	0.957	0.981
$\hat{\sigma}_\phi$	0.268	0.033	0.208	0.267	0.333
$\hat{\rho}_\delta$	0.931	0.065	0.765	0.950	0.996
$\hat{\sigma}_\delta$	0.270	0.045	0.187	0.267	0.356
$\hat{\sigma}_\psi$	0.786	0.069	0.664	0.783	0.927
$\hat{\rho}_\omega$	0.317	0.025	0.267	0.317	0.365
$\hat{\sigma}_\omega$	1.277	0.021	1.237	1.277	1.315

Supplementary Table 3: Posterior estimates of the parameters of the multiple likelihood model for EUR

Parameter	Mean	Std. dev.	2.5%	50%	97.5%
$\hat{\lambda}^{(a)}$	0.262	0.295	−0.320	0.259	0.842
$\hat{\lambda}^{(o)}$	0.282	0.295	−0.302	0.279	0.864
$\hat{\lambda}^{(s)}$	−0.247	0.295	−0.832	−0.250	0.338
$\hat{\sigma}_1$	0.464	0.010	0.444	0.464	0.484
$\hat{\sigma}_2$	0.421	0.010	0.400	0.421	0.441
$\hat{\sigma}_3$	0.399	0.001	0.398	0.400	0.400
$\hat{\sigma}_\beta$	0.241	0.174	0.011	0.216	0.629
$\hat{\sigma}_\alpha$	3.905	2.232	1.172	3.428	9.442
$\hat{\rho}_\gamma$	0.122	0.583	−0.899	0.160	0.981
$\hat{\sigma}_\gamma$	0.043	0.028	0.004	0.038	0.109
$\hat{\rho}_\phi$	0.957	0.011	0.930	0.959	0.975
$\hat{\sigma}_\phi$	0.247	0.018	0.213	0.247	0.282
$\hat{\rho}_\delta$	0.977	0.023	0.914	0.984	0.999
$\hat{\sigma}_\delta$	0.120	0.021	0.082	0.119	0.163
$\hat{\sigma}_\psi$	0.744	0.061	0.629	0.743	0.868
$\hat{\rho}_\omega$	0.653	0.026	0.603	0.652	0.704
$\hat{\sigma}_\omega$	0.674	0.013	0.650	0.674	0.699

Supplementary Table 4: Posterior estimates of the parameters of the multiple likelihood model for EMR

Parameter	Mean	Std. dev.	2.5%	50%	97.5%
$\hat{\lambda}^{(a)}$	0.519	0.305	−0.085	0.523	1.102
$\hat{\lambda}^{(o)}$	0.519	0.305	−0.077	0.522	1.106
$\hat{\lambda}^{(s)}$	−0.658	0.305	−1.261	−0.653	−0.077
$\hat{\sigma}_1$	0.768	0.020	0.730	0.768	0.808
$\hat{\sigma}_2$	0.690	0.019	0.654	0.689	0.728
$\hat{\sigma}_3$	0.399	0.001	0.396	0.399	0.400
$\hat{\sigma}_\beta$	1.329	0.488	0.183	1.362	2.228
$\hat{\sigma}_\alpha$	2.599	1.837	0.652	2.202	7.015
$\hat{\rho}_\gamma$	0.607	0.498	−0.662	0.862	0.999
$\hat{\sigma}_\gamma$	0.115	0.062	0.015	0.112	0.243
$\hat{\rho}_\phi$	0.899	0.048	0.795	0.903	0.972
$\hat{\sigma}_\phi$	0.485	0.038	0.413	0.485	0.560
$\hat{\rho}_\delta$	0.348	0.547	−0.826	0.481	0.986
$\hat{\sigma}_\delta$	0.054	0.039	0.004	0.048	0.138
$\hat{\sigma}_\psi$	0.593	0.077	0.452	0.588	0.756
$\hat{\rho}_\omega$	0.499	0.046	0.407	0.500	0.587
$\hat{\sigma}_\omega$	0.763	0.025	0.715	0.763	0.813

Supplementary Table 5: Posterior estimates of the parameters of the multiple likelihood model for SEAR

Parameter	Mean	Std. dev.	2.5%	50%	97.5%
$\hat{\lambda}^{(a)}$	0.431	0.293	−0.135	0.428	1.005
$\hat{\lambda}^{(o)}$	0.105	0.292	−0.462	0.102	0.673
$\hat{\lambda}^{(s)}$	−0.202	0.293	−0.779	−0.202	0.375
$\hat{\sigma}_1$	1.410	0.042	1.330	1.409	1.494
$\hat{\sigma}_2$	0.949	0.031	0.890	0.948	1.012
$\hat{\sigma}_3$	0.398	0.003	0.391	0.398	0.400
$\hat{\sigma}_\beta$	1.284	0.402	0.733	1.208	2.267
$\hat{\sigma}_\alpha$	2.551	1.648	0.807	2.149	6.695
$\hat{\rho}_\gamma$	0.877	0.249	−0.002	0.960	0.999
$\hat{\sigma}_\gamma$	0.162	0.061	0.046	0.158	0.295
$\hat{\rho}_\phi$	0.469	0.194	0.080	0.474	0.847
$\hat{\sigma}_\phi$	0.459	0.042	0.379	0.458	0.542
$\hat{\rho}_\delta$	0.272	0.340	−0.326	0.240	0.958
$\hat{\sigma}_\delta$	0.190	0.049	0.098	0.188	0.288
$\hat{\sigma}_\psi$	0.252	0.156	0.017	0.248	0.561
$\hat{\rho}_\omega$	0.860	0.052	0.729	0.869	0.935
$\hat{\sigma}_\omega$	0.322	0.039	0.247	0.321	0.405

Supplementary Table 6: Posterior estimates of the parameters of the multiple likelihood model for the WPR

Parameter	Mean	Std. dev.	2.5%	50%	97.5%
$\hat{\lambda}^{(a)}$	0.508	0.295	−0.059	0.505	1.086
$\hat{\lambda}^{(o)}$	0.558	0.295	−0.006	0.555	1.139
$\hat{\lambda}^{(s)}$	−0.689	0.296	−1.260	−0.690	−0.118
$\hat{\sigma}_1$	0.870	0.022	0.827	0.870	0.914
$\hat{\sigma}_2$	0.858	0.021	0.817	0.857	0.899
$\hat{\sigma}_3$	0.399	0.001	0.396	0.399	0.400
$\hat{\sigma}_\beta$	1.277	0.228	0.891	1.256	1.780
$\hat{\sigma}_\alpha$	3.116	1.840	0.798	2.757	7.687
$\hat{\rho}_\gamma$	0.408	0.533	−0.749	0.541	0.999
$\hat{\sigma}_\gamma$	0.072	0.052	0.005	0.063	0.194
$\hat{\rho}_\phi$	0.687	0.075	0.533	0.689	0.830
$\hat{\sigma}_\phi$	0.561	0.038	0.488	0.561	0.635
$\hat{\rho}_\delta$	0.527	0.558	−0.779	0.826	0.997
$\hat{\sigma}_\delta$	0.091	0.051	0.002	0.095	0.188
$\hat{\sigma}_\psi$	0.596	0.087	0.425	0.596	0.772
$\hat{\rho}_\omega$	0.617	0.041	0.536	0.617	0.697
$\hat{\sigma}_\omega$	0.823	0.030	0.764	0.824	0.882

Supplementary Table 7: Summary of the differences between WUENIC and modelled estimates for each WHO region and globally.

Region	Median difference	Interquartile range
EMR	-1.33	4.59
AMR	-4.62	6.59
EUR	-1.89	2.75
AFR	0.21	10.35
WPR	-3.22	7.13
SEAR	-0.36	3.22
Global	-1.90	5.44

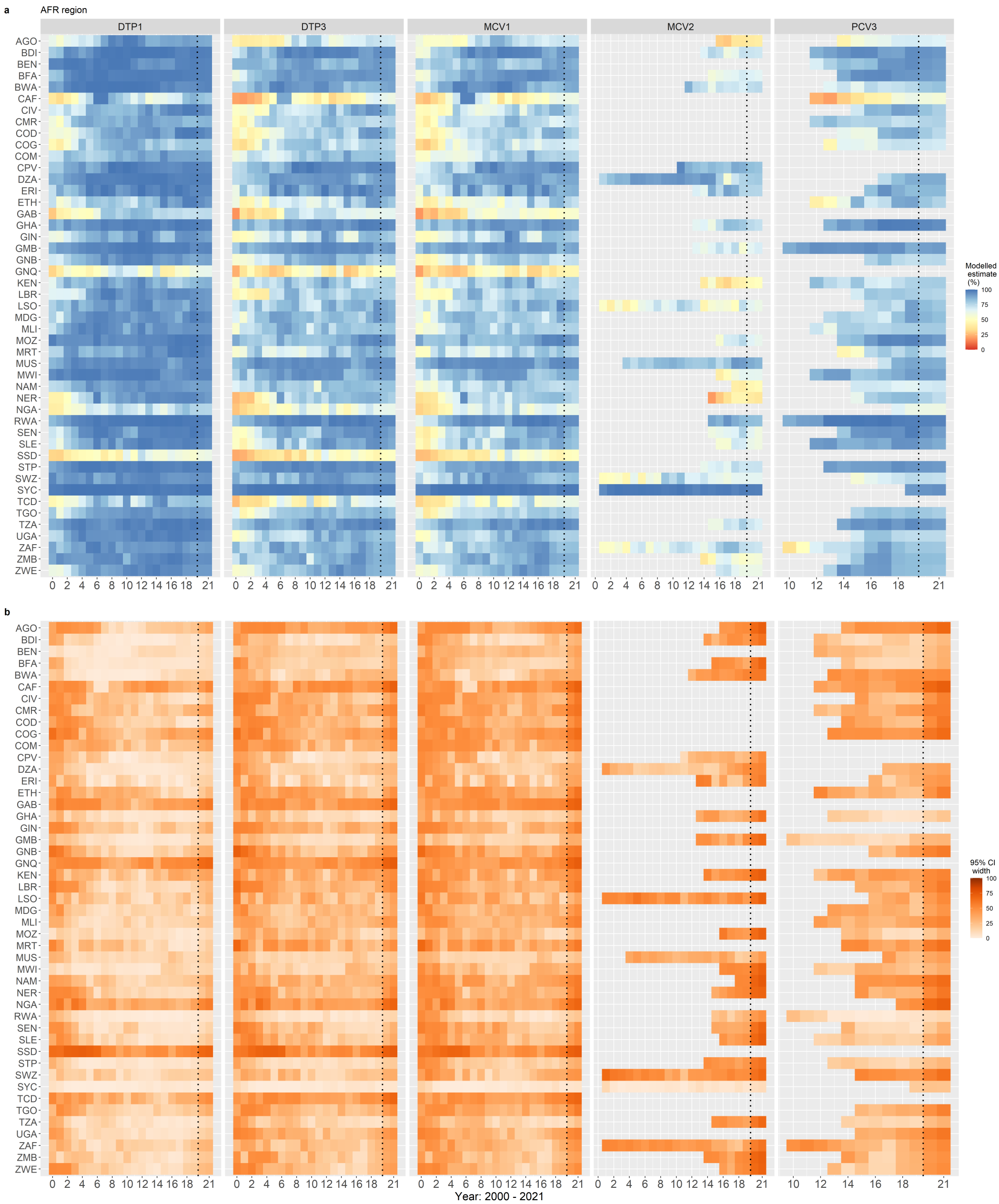

Supplementary Figure 13: Plots of modelled estimates and corresponding uncertainties at the country level for AFR

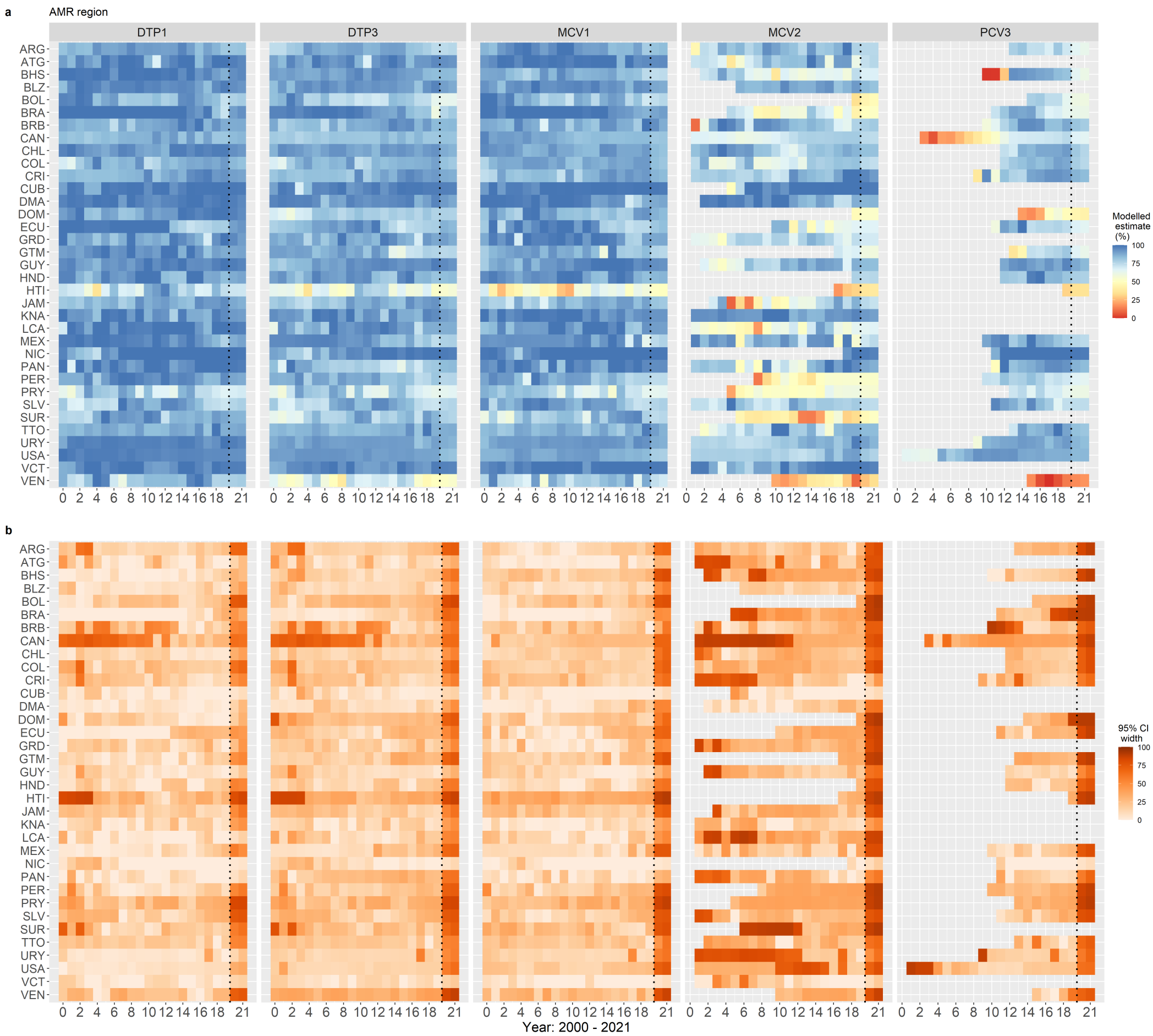

Supplementary Figure 14: Plots of modelled estimates and corresponding uncertainties at the country level for AMR

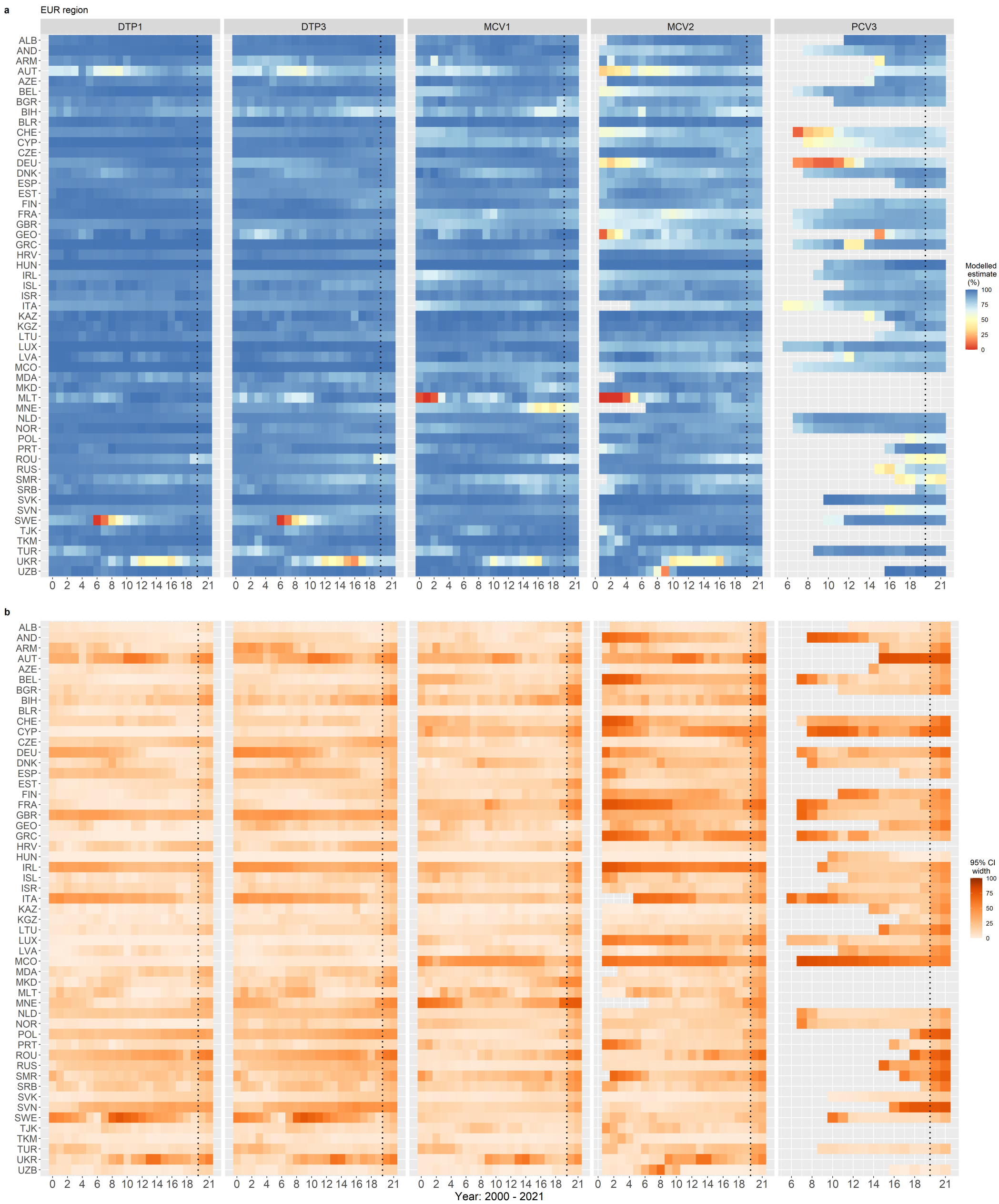

Supplementary Figure 15: Plots of modelled estimates and corresponding uncertainties at the country level for the EUR

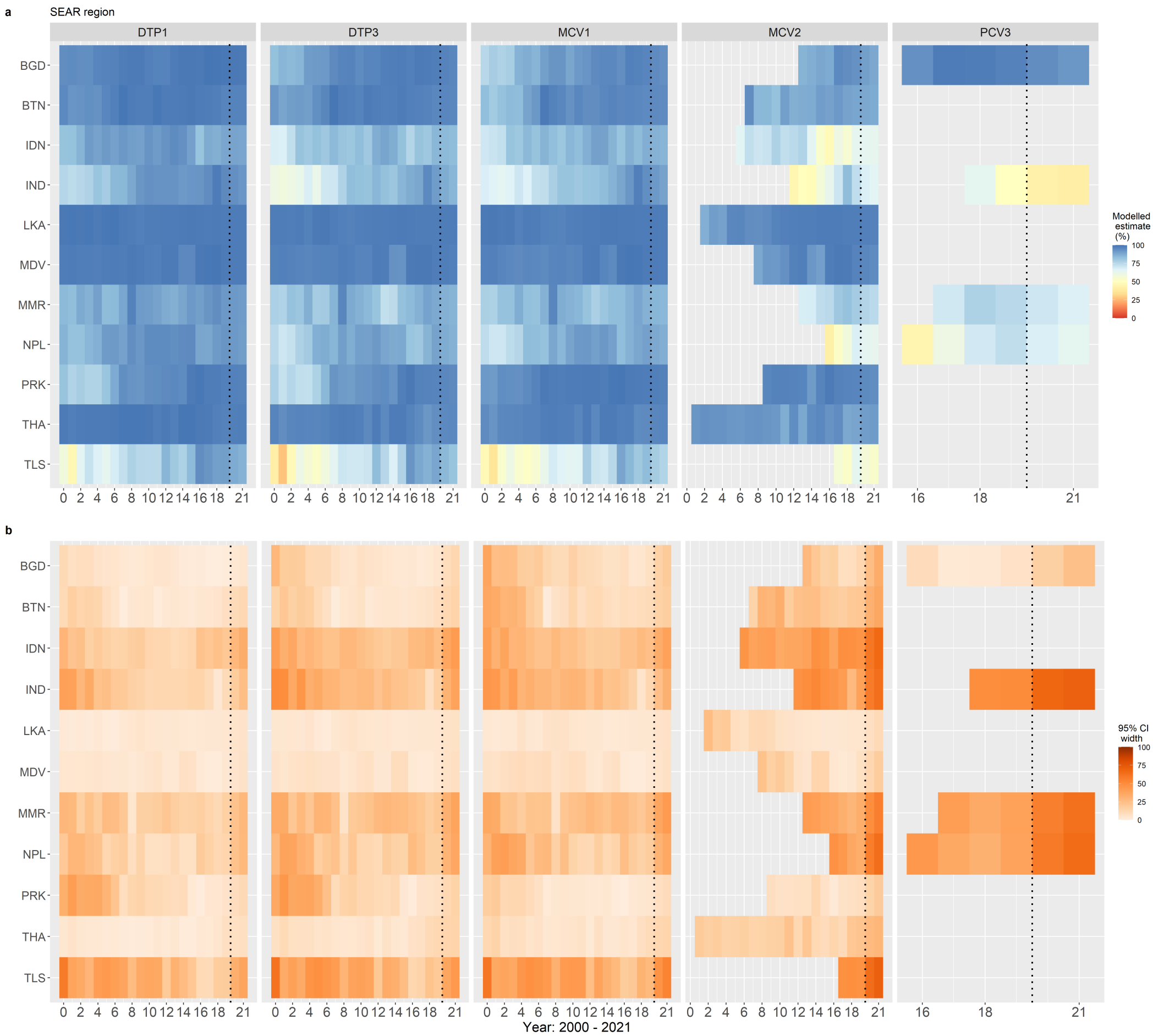

Supplementary Figure 16: Plots of modelled estimates and corresponding uncertainties at the country level for the SEAR

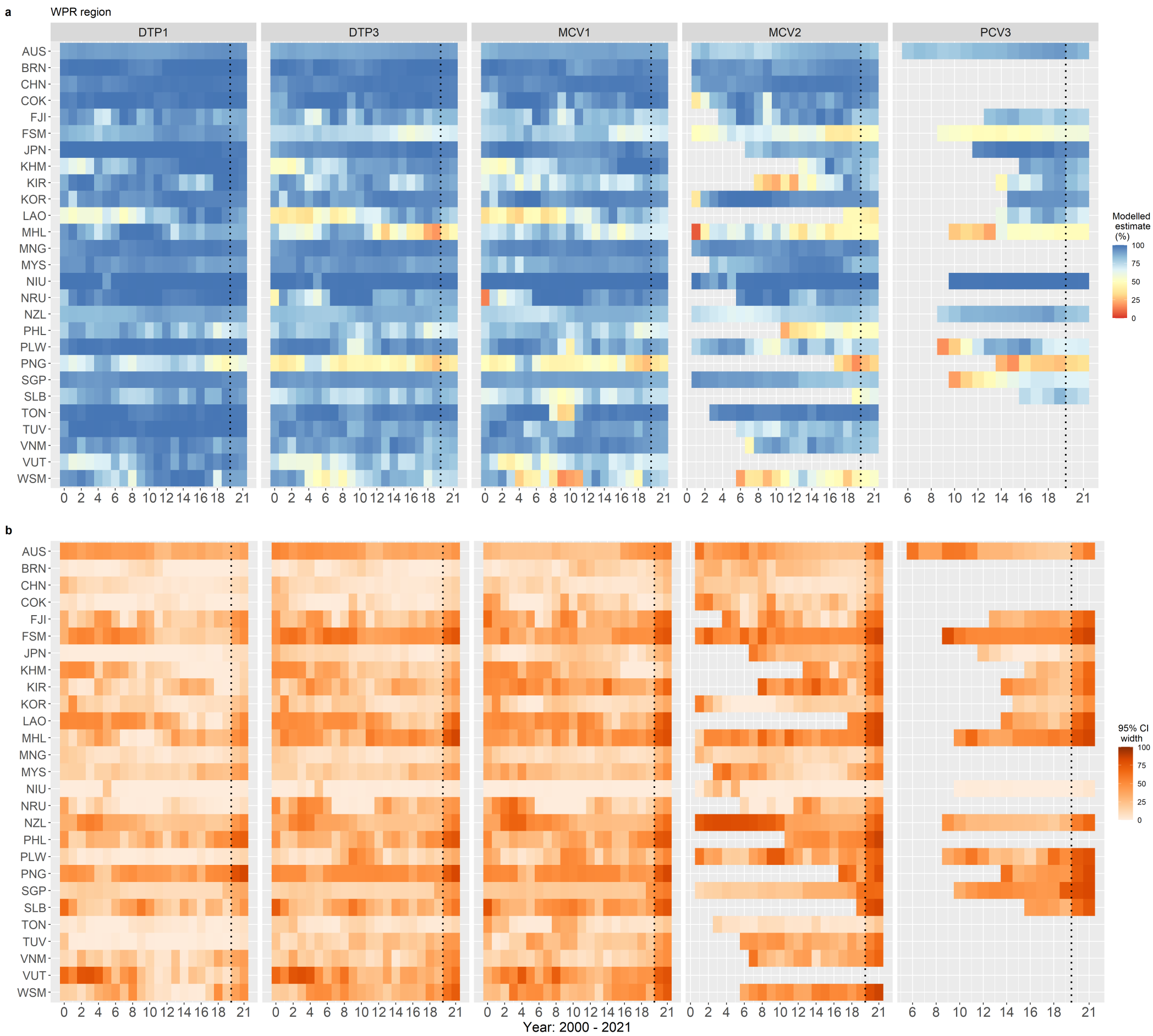

Supplementary Figure 17: Plots of modelled estimates and corresponding uncertainties at the country level for the WPR

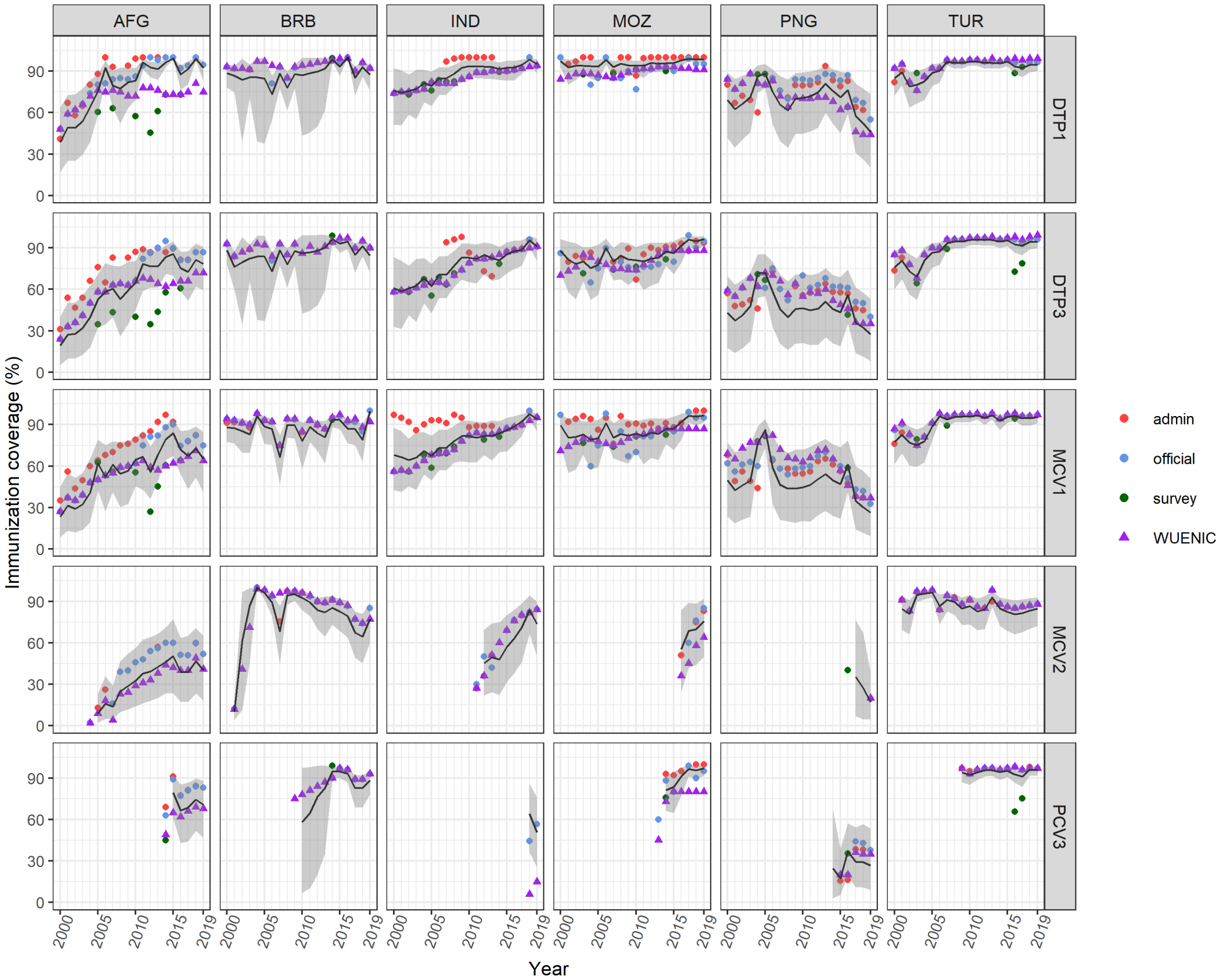

Supplementary Figure 18: Modelled estimates of immunization coverage (black line) and corresponding uncertainty estimates (grey shaded area) for select countries. The plots are overlaid with processed input administrative, official and survey data, as well as corresponding WUENIC estimates. Predictions for 2021 and 2022 are shown on the right-hand side of the dotted vertical lines.

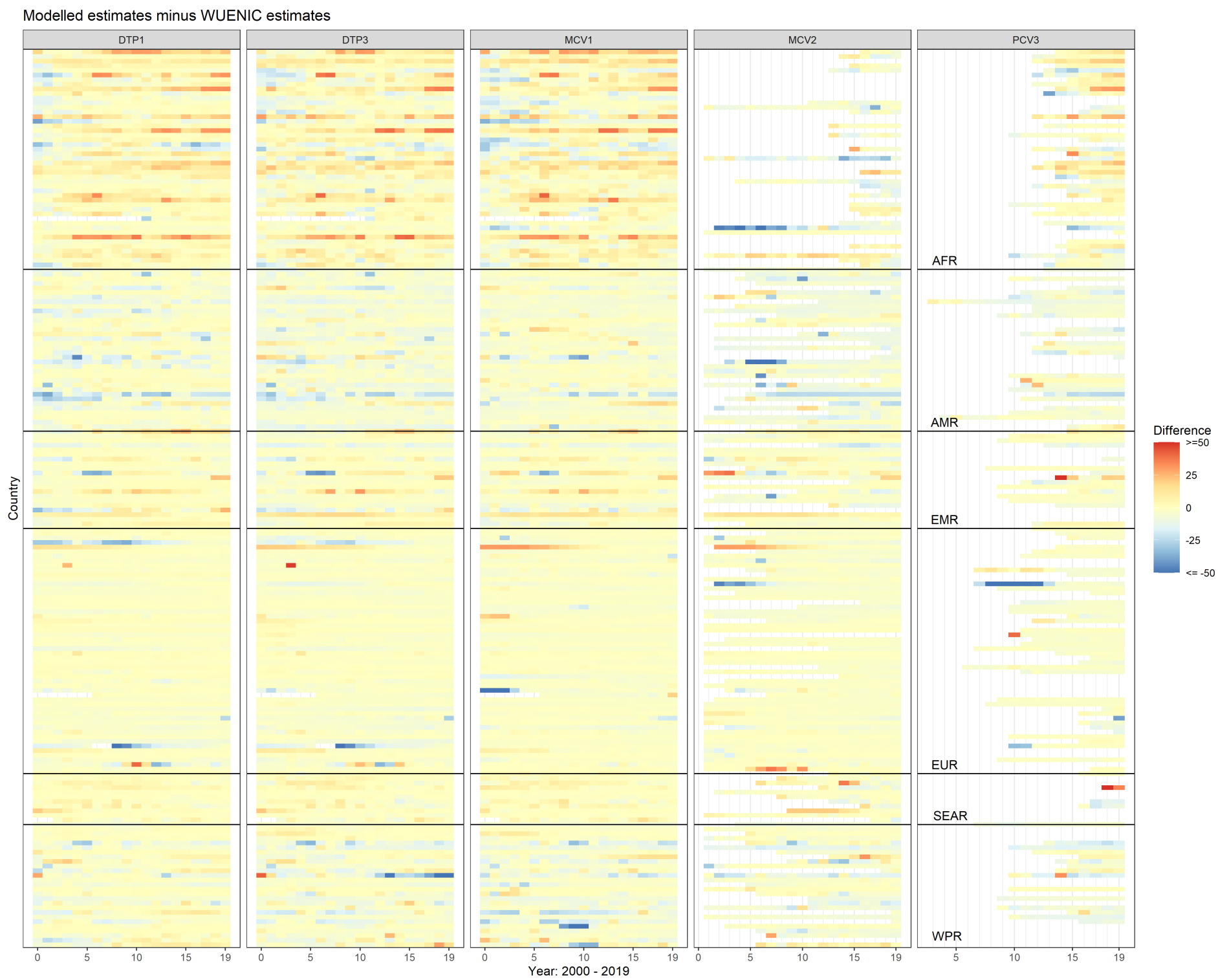

Supplementary Figure 19: Differences between WUENIC estimates and the modelled estimates for all WHO countries.

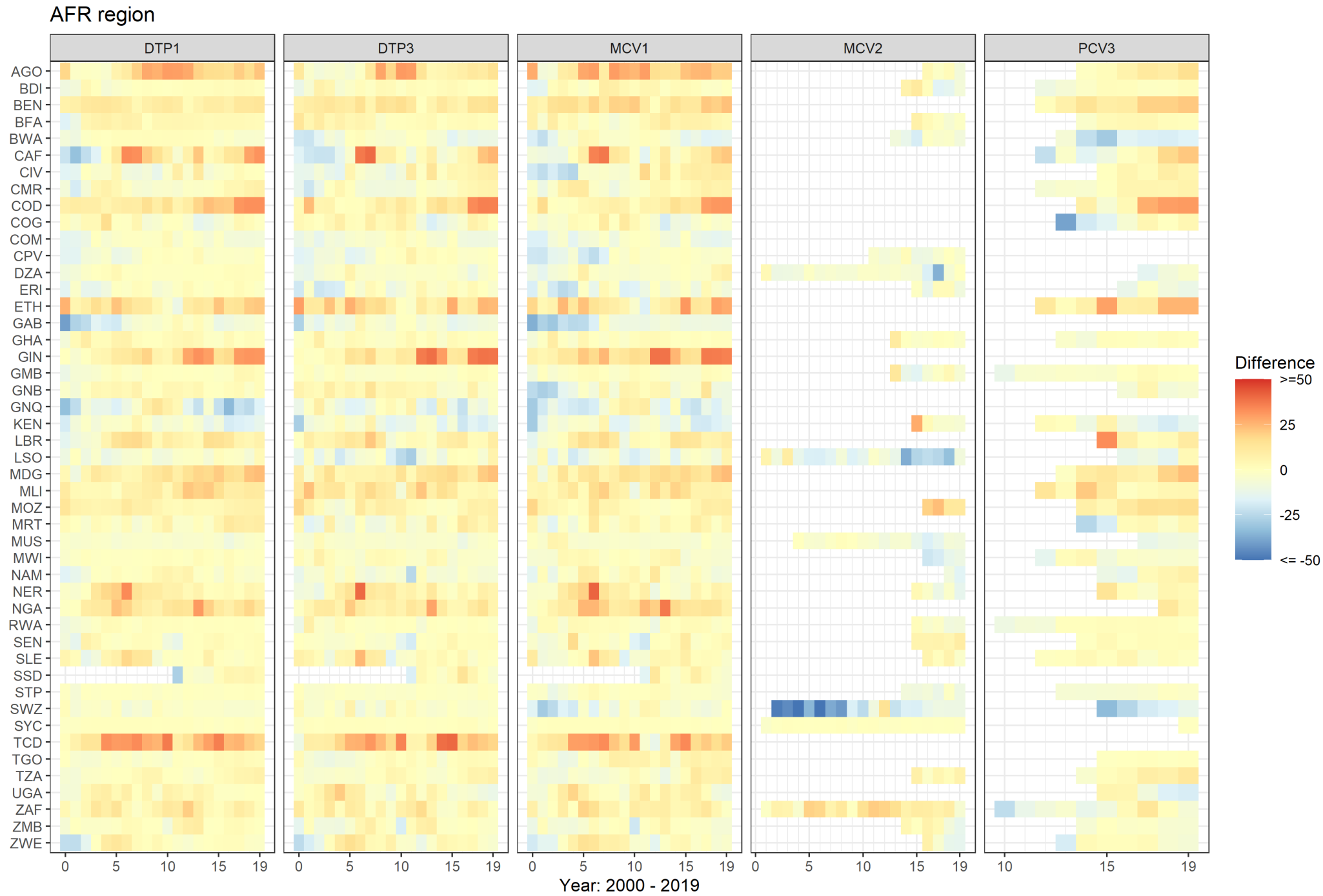

Supplementary Figure 20: Comparing WUENIC estimates with the modelled estimates for AFR

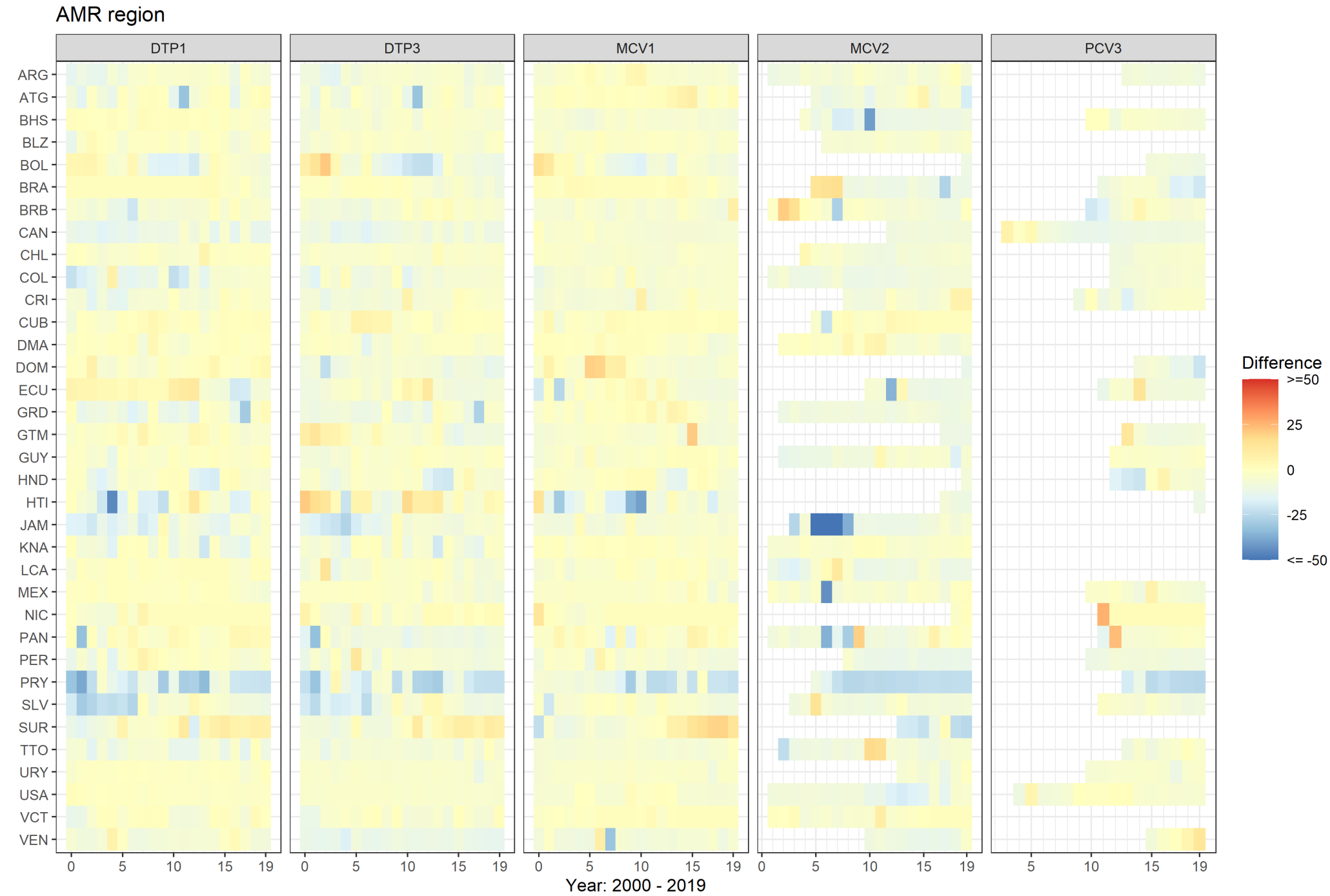

Supplementary Figure 21: Comparing WUENIC estimates with the modelled estimates for AMR

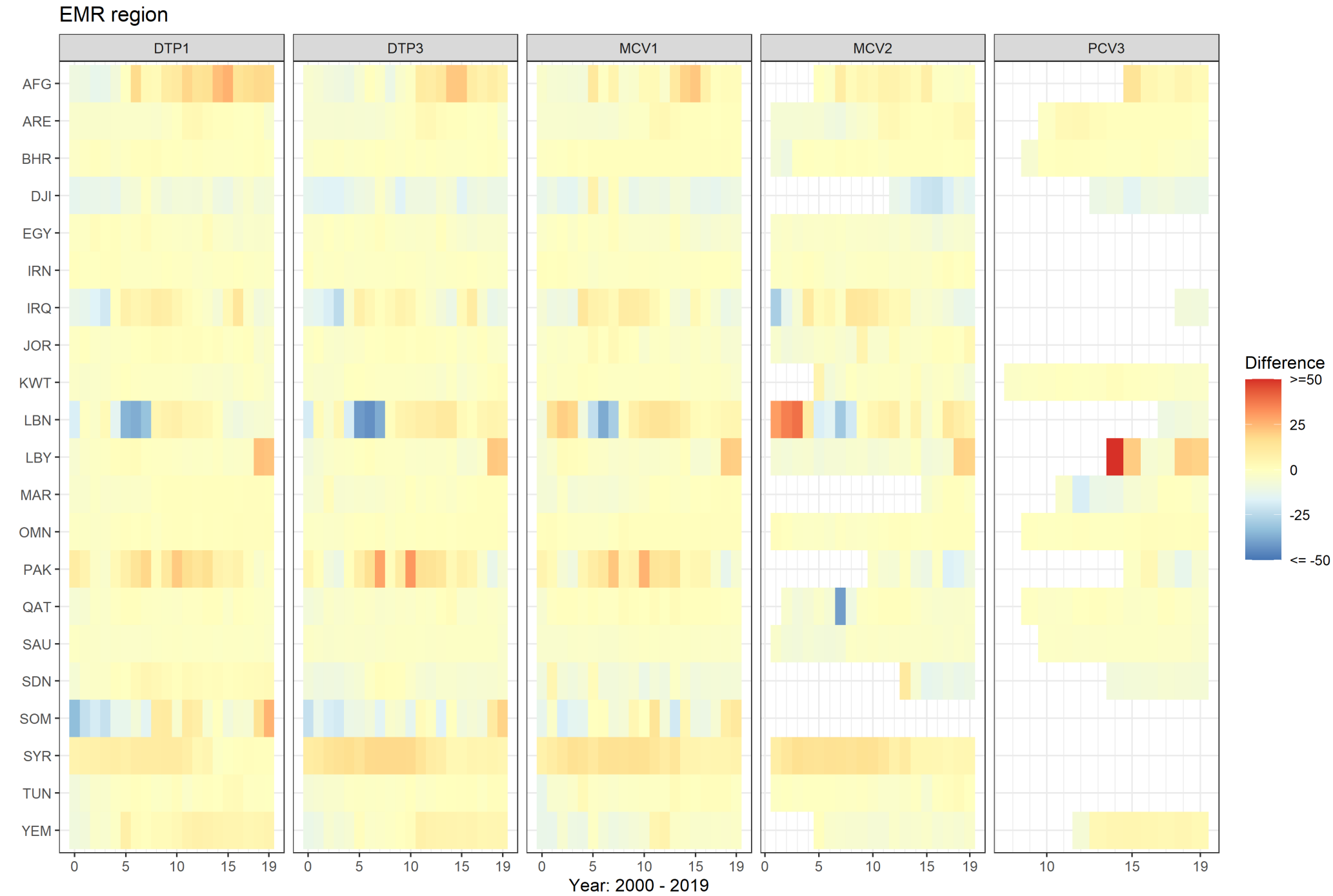

Supplementary Figure 22: Comparing WUENIC estimates with the modelled estimates for EMR

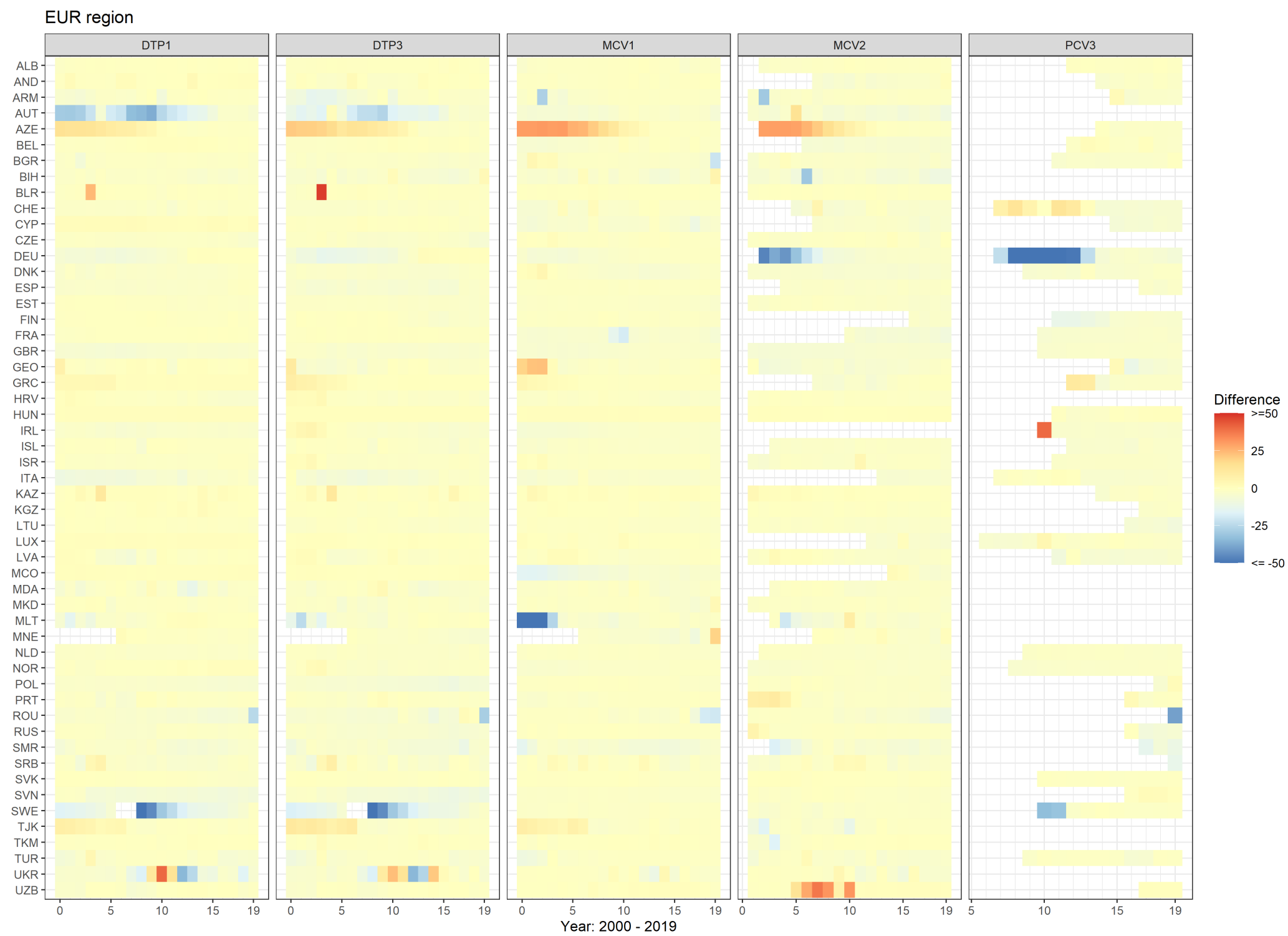

Supplementary Figure 23: Comparing WUENIC estimates with the modelled estimates for EUR

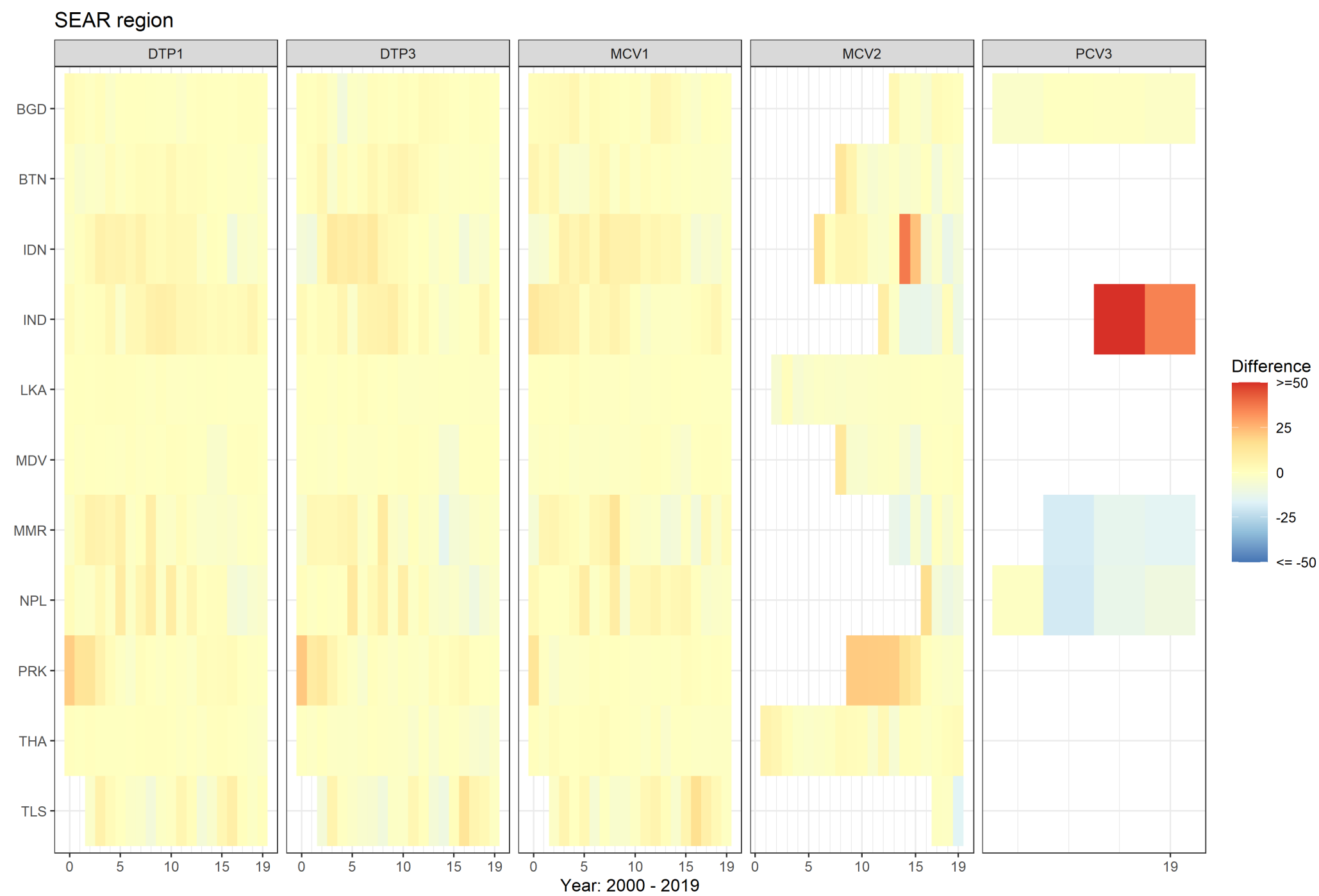

Supplementary Figure 24: Comparing WUENIC estimates with the modelled estimates for SEAR

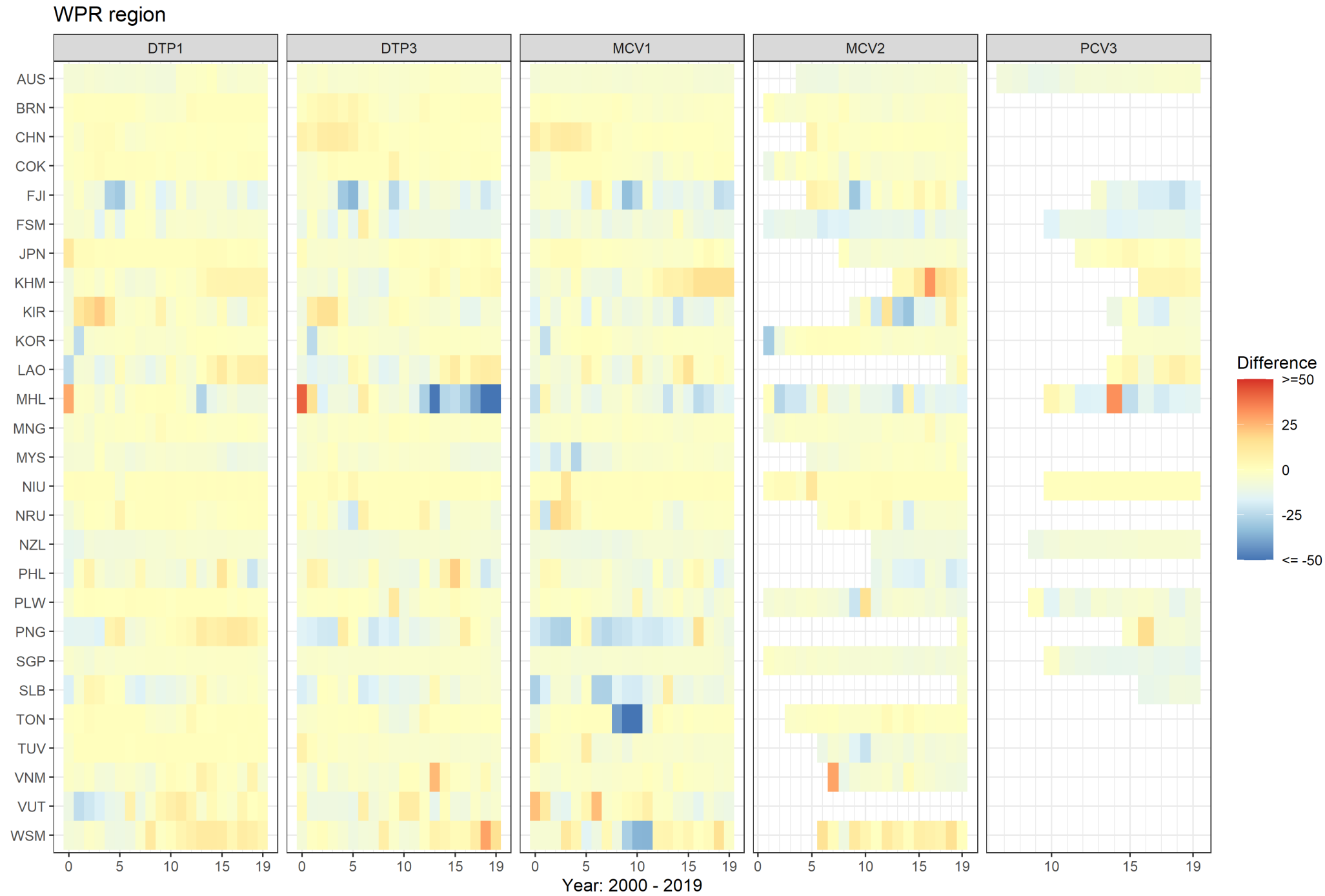

Supplementary Figure 25: Comparing WUENIC estimates with the modelled estimates for WPR

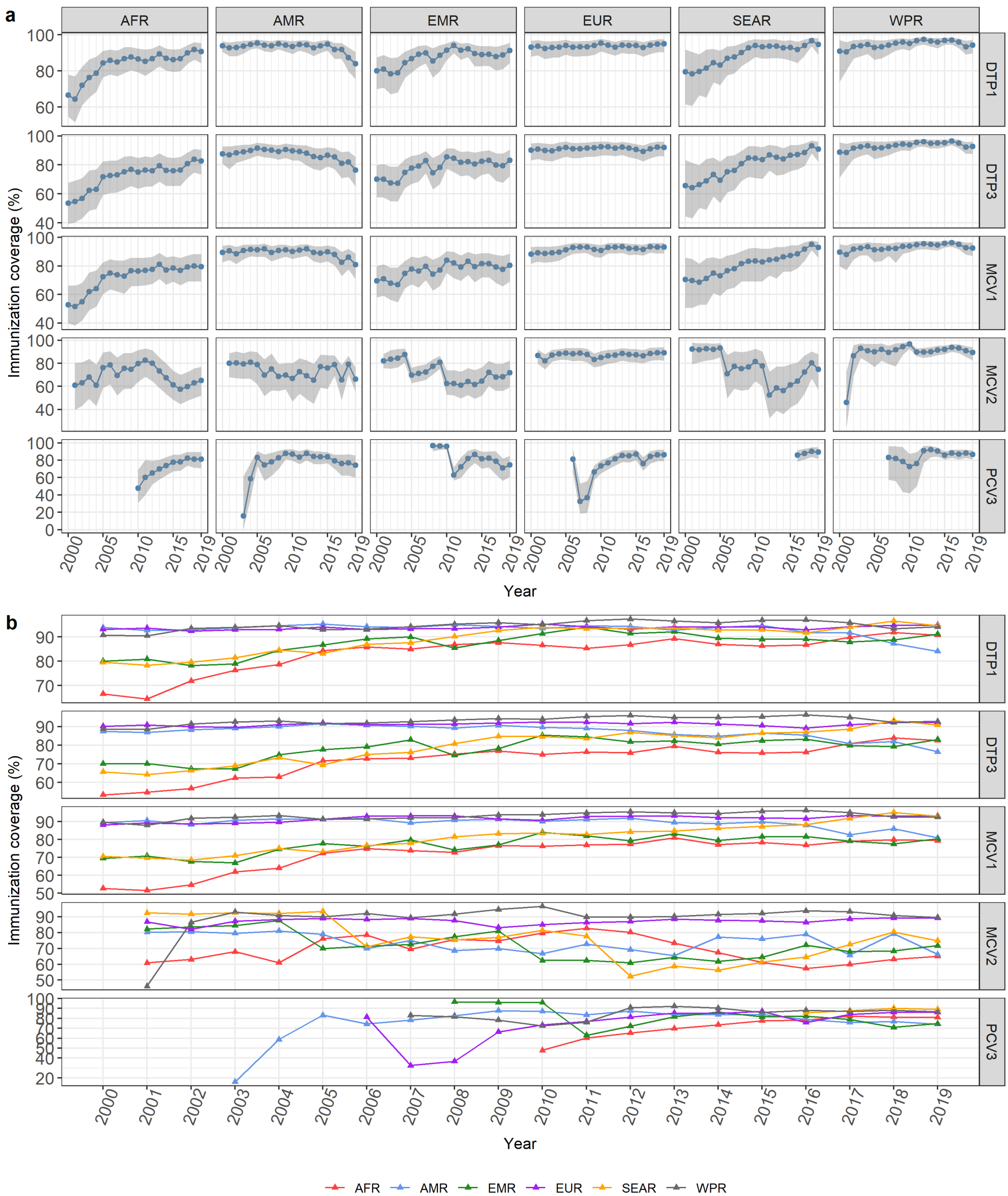

Supplementary Figure 26: (a) Modelled estimates of immunization coverage and corresponding uncertainties for each vaccine and WHO region; (b) Regional trends in modelled estimates of coverage for each vaccine.

4. Processing code

```
# Global immunisation coverage modelling

library(imcover)
library(rstan)

options(mc.cores = parallel::detectCores())

# path to files - note: change here
dir <- '/main/path/to/workspace'

# get WUENIC input files
cov <- download_coverage(use_cache = TRUE)
svy <- download_survey(use_cache = TRUE)

# drop some categories (PAB, HPV, WUENIC) and clean up
cov <- cov[cov$coverage_category %in% c("ADMIN", "OFFICIAL"), ]
cov$coverage_category <- tolower(cov$coverage_category)

# combined dataset
dat <- rbind(cov, svy)

# remove records with missing values
dat <- dat[!is.na(dat$region), ]
dat <- dat[!is.na(dat$coverage), ]

# mismatch in vaccine names between coverage and survey datasets
dat[dat$antigen == 'DTPCV1', 'antigen'] <- 'DTP1'
dat[dat$antigen == 'DTPCV2', 'antigen'] <- 'DTP2'
dat[dat$antigen == 'DTPCV3', 'antigen'] <- 'DTP3'

# select only some vaccines to analyse
dat <- ic_filter(dat, vaccine = c("DTP1", "DTP3", "MCV1", "MCV2", "PCV3"))
  table(dat$coverage_category, dat$antigen)

# subset the time
dat <- ic_filter(dat, time = 2000:2020)

# drop observations with zero values
  table(dat$coverage == 0)  # n=165
dat <- dat[dat$coverage > 0, ]

# adjustment - use ratio for DTP3 and requiring 0 < ic < 100
dat <- ic_ratio(dat, numerator = 'DTP3', denominator = 'DTP1')
```

```
dat <- ic_adjust(dat, coverage_adj = TRUE)

# model fitting - multiple likelihood model
fit <- ic_fit(dat,
              chains = 4,
              iter = 2000,
              warmup = 1000,
              prior_lambda = c(0.5, 0.5, 0.5),
              prior_sigma = c(2, 2, 0.2),
              upper_sigma = c(100, 100, 0.4),
              lower_sigma = c(0, 0, 0))

# save the fitted object
saveRDS(fit, file = file.path(dir, 'output', 'fit.rds'))

# post-processing
regs <- names(fit) # regions

# WAIC calculation
waics <- lapply(regs, function(r){
  print(r)
  ww <- waic(fit[[r]])
  print(ww)
})

# coverage estimates and predictions
# loop over the regions in the fitted object
for(r in regs){
  print(r)
  # extract region
  f <- fit[[r]]

  # filter based on year of introduction
  f <- filter_yovi(f)

  # predict for 2021 and 2022
  f <- predict(f, t=2)

  # summarise coverage estimates
  ic_post <- ic_coverage(f,
                         object = 'posterior',
                         stat = c('mean', 'quantile'),
                         probs = c(0.025, 0.5, 0.975))
  # and predictions
  ic_pred <- ic_coverage(f,
                         object = 'prediction',
                         stat = c('mean', 'quantile'),
                         probs = c(0.025, 0.5, 0.975))

```

```
  ic <- rbind(ic_post, ic_pred)
  ic <- ic[order(ic$country, ic$vaccine, ic$time),]

  # write out table of coverage
  write.csv(ic, file.path(dir, 'output', paste0(r, '_coverage.
   csv')),
            row.names = F)
}

# regional aggregation summaries
# first load the population denominator
denom <- read.csv(file.path(dir, 'input', 'denom.csv'))
# this was created with: denom <- imcover::download_wuenic()

# process each region ...
for(r in regs){
  print(r)

  # population-weighted summary
  icr <- ic_regional(fit[[r]],
           denom = denom,
           filter_yovi = F)

  # create output table
  write.csv(icr,
      file.path(dir, 'output', paste0(r, '_regional_coverage.
   csv')),
      row.names = F)
}
```

References

[1] R Core Team, R: A Language and Environment for Statistical Computing, R Foundation for Statistical Computing, Vienna, Austria (2021).
URL https://www.R-project.org/

[2] Stan Development Team, Stan modeling language: Users guide and reference manual, Columbia University, Columbia, New York (2015).
URL https://mc-stan.org/documentation

[3] World Health Organization and United Nations Children's Fund, WHO/UNICEF joint reporting process (2022).
URL https://www.who.int/teams/immunization-vaccines-and-biologicals/immunization-analysis-and-insights/global-monitoring/who-unicef-joint-reporting-process

[4] F. T. Cutts, H. S. Izurieta, D. A. Rhoda, Measuring coverage in MNCH: Design,

implementation, and interpretation challenges associated with tracking vaccination coverage using household surveys, PLOS Medicine 10 (5) (2013) 1–11. doi:10.1371/journal.pmed.1001404.
URL https://doi.org/10.1371/journal.pmed.1001404

[5] F. T. Cutts, P. Claquin, M. C. Danovaro-Holliday, D. A. Rhoda, Monitoring vaccination coverage: Defining the role of surveys, Vaccine 34 (35) (2016) 4103–4109. doi:https://doi.org/10.1016/j.vaccine.2016.06.053.
URL https://www.sciencedirect.com/science/article/pii/S0264410X16304777
