# Supplementary material for: Bayesian Hierarchical Modeling Approaches for Combining Information From Multiple Data Sources to Produce Annual Estimates of National Immunization Coverage
Source: Biom J. 2026 Oct 6;68(5):e70189. doi: 10.1002/bimj.70189 (PMC13652340; doi:10.1002/bimj.70189)
Supplement: Supplementary file 2 [file BIMJ-68-e70189-s002.zip › code_and_data/outputs/Figure_4.pdf]

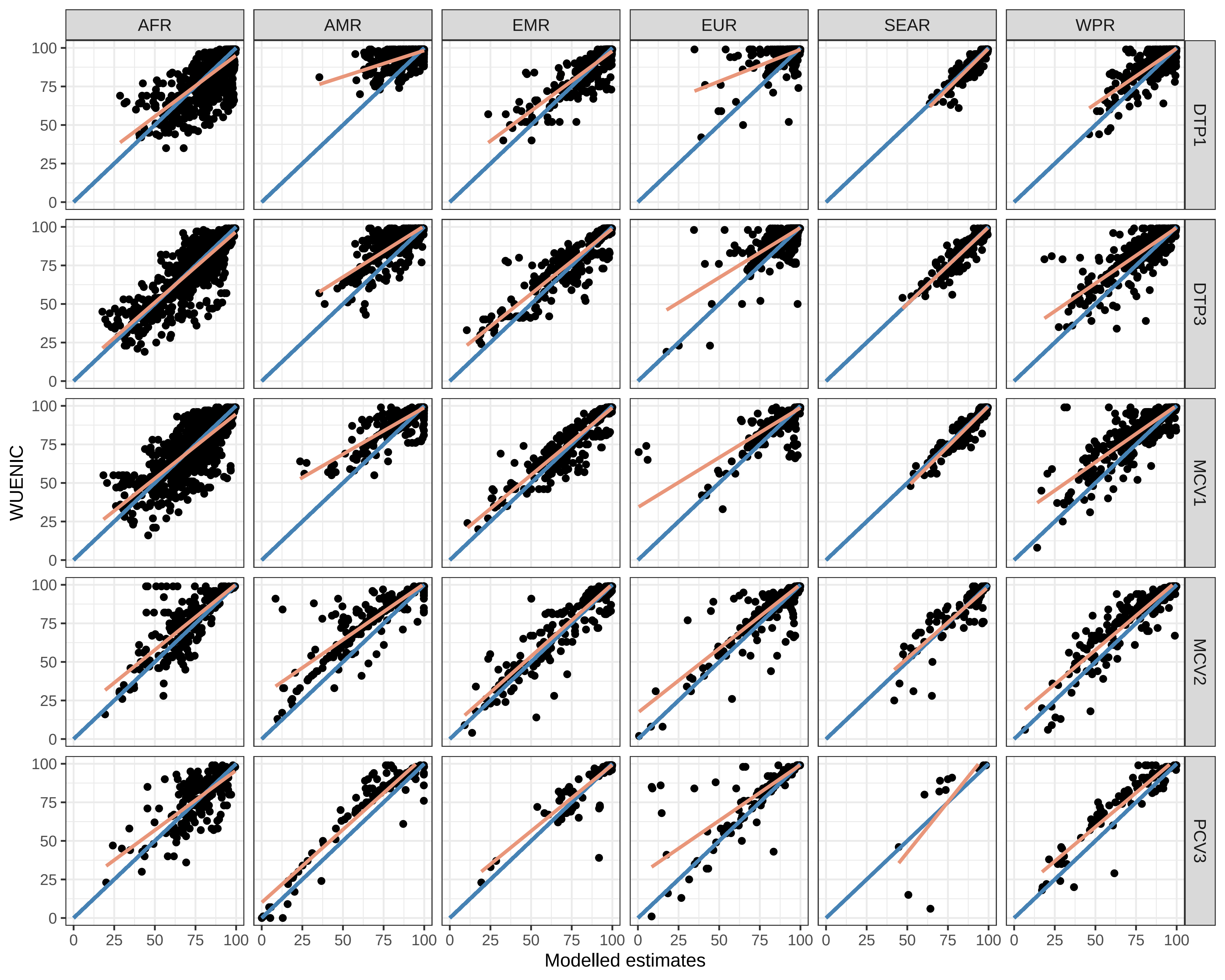
AFR
AMR
EMR
EUR
SEAR
WPR
DTP1
DTP3
MCV1
MCV2
PCV3
WUENIC
Modelled estimates
0
25
50
75
100
