# Supplementary material for: Bayesian Hierarchical Modeling Approaches for Combining Information From Multiple Data Sources to Produce Annual Estimates of National Immunization Coverage
Source: Biom J. 2026 Oct 6;68(5):e70189. doi: 10.1002/bimj.70189 (PMC13652340; doi:10.1002/bimj.70189)
Supplement: Supplementary file 2 [file BIMJ-68-e70189-s002.zip › code_and_data/outputs/Figure_S12.pdf]

a

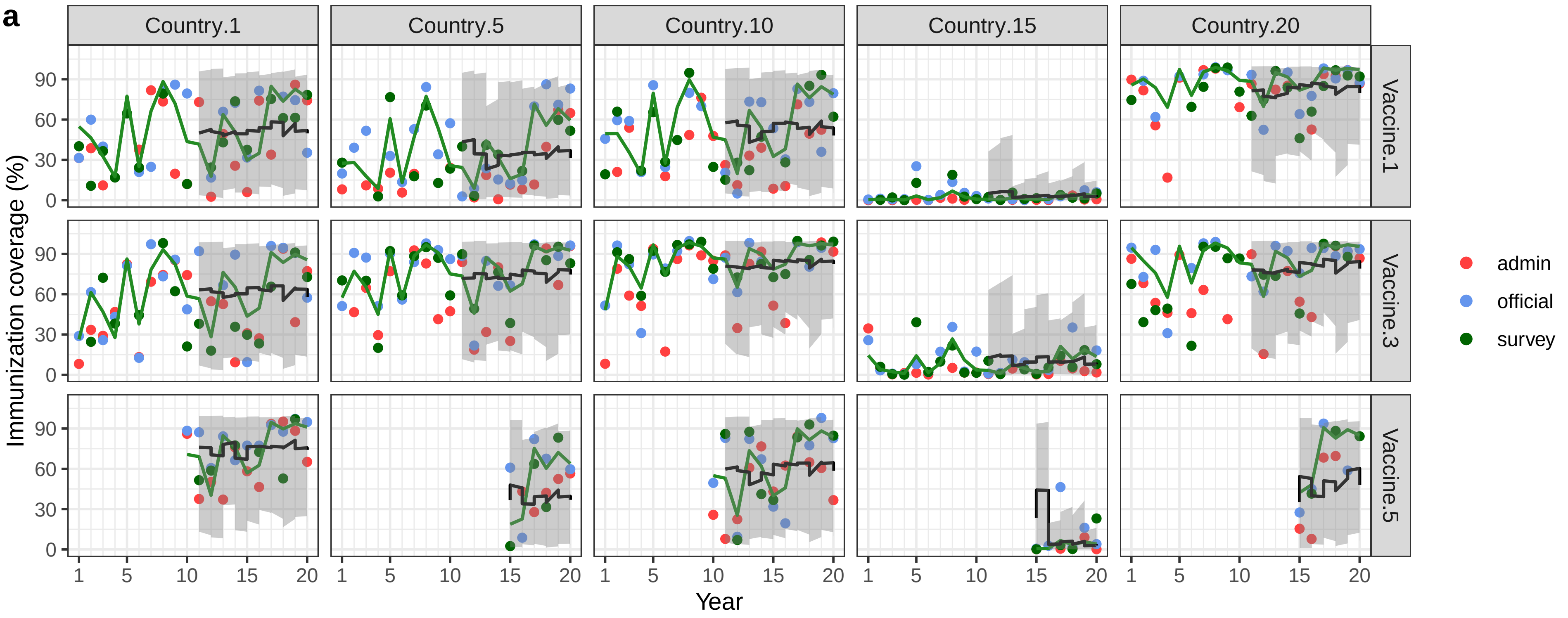

Country.1
Country.5
Country.10
Country.15
Country.20
Vaccine.1
Vaccine.3
Vaccine.5
Immunization coverage (%)
Year
admin
official
survey

b

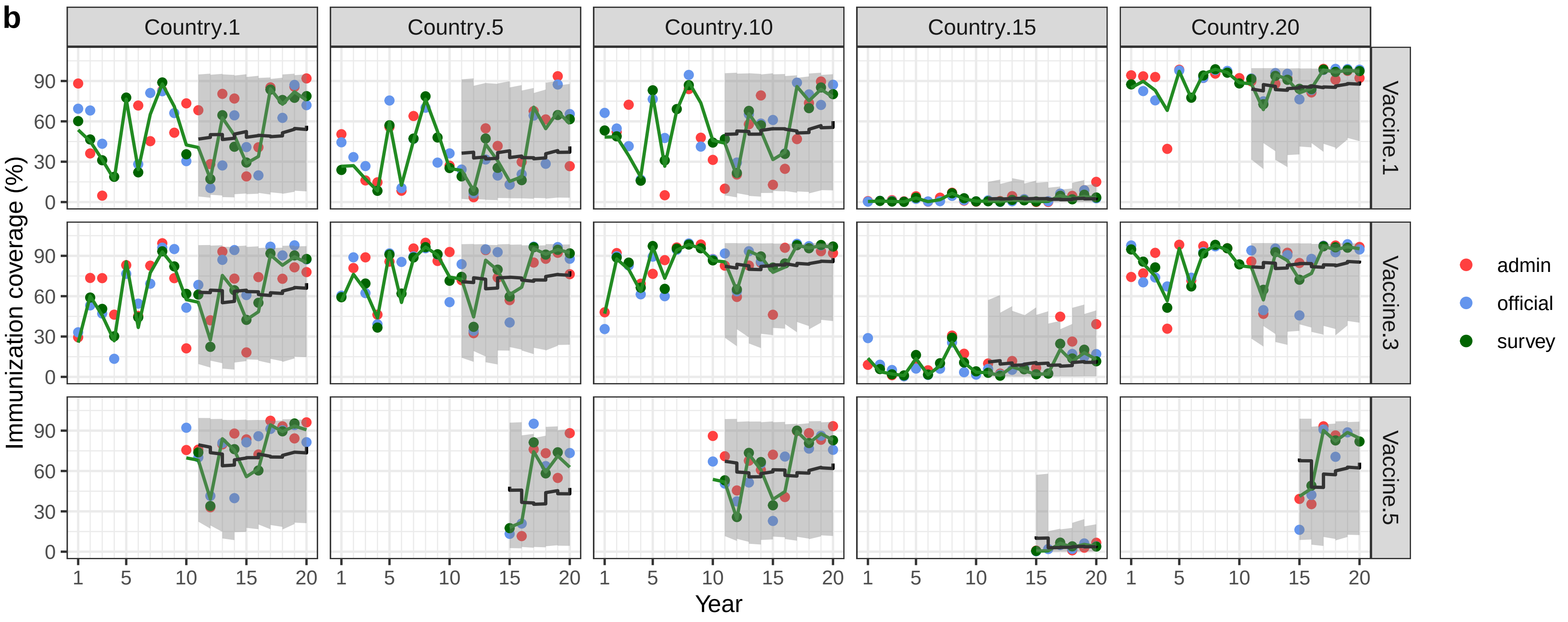

Country.1
Country.5
Country.10
Country.15
Country.20
Vaccine.1
Vaccine.3
Vaccine.5
Immunization coverage (%)
Year
admin
official
survey
