# Supplementary material for: Bayesian Hierarchical Modeling Approaches for Combining Information From Multiple Data Sources to Produce Annual Estimates of National Immunization Coverage
Source: Biom J. 2026 Oct 6;68(5):e70189. doi: 10.1002/bimj.70189 (PMC13652340; doi:10.1002/bimj.70189)
Supplement: Supplementary file 2 [file BIMJ-68-e70189-s002.zip › code_and_data/outputs/Figure_S13.pdf]

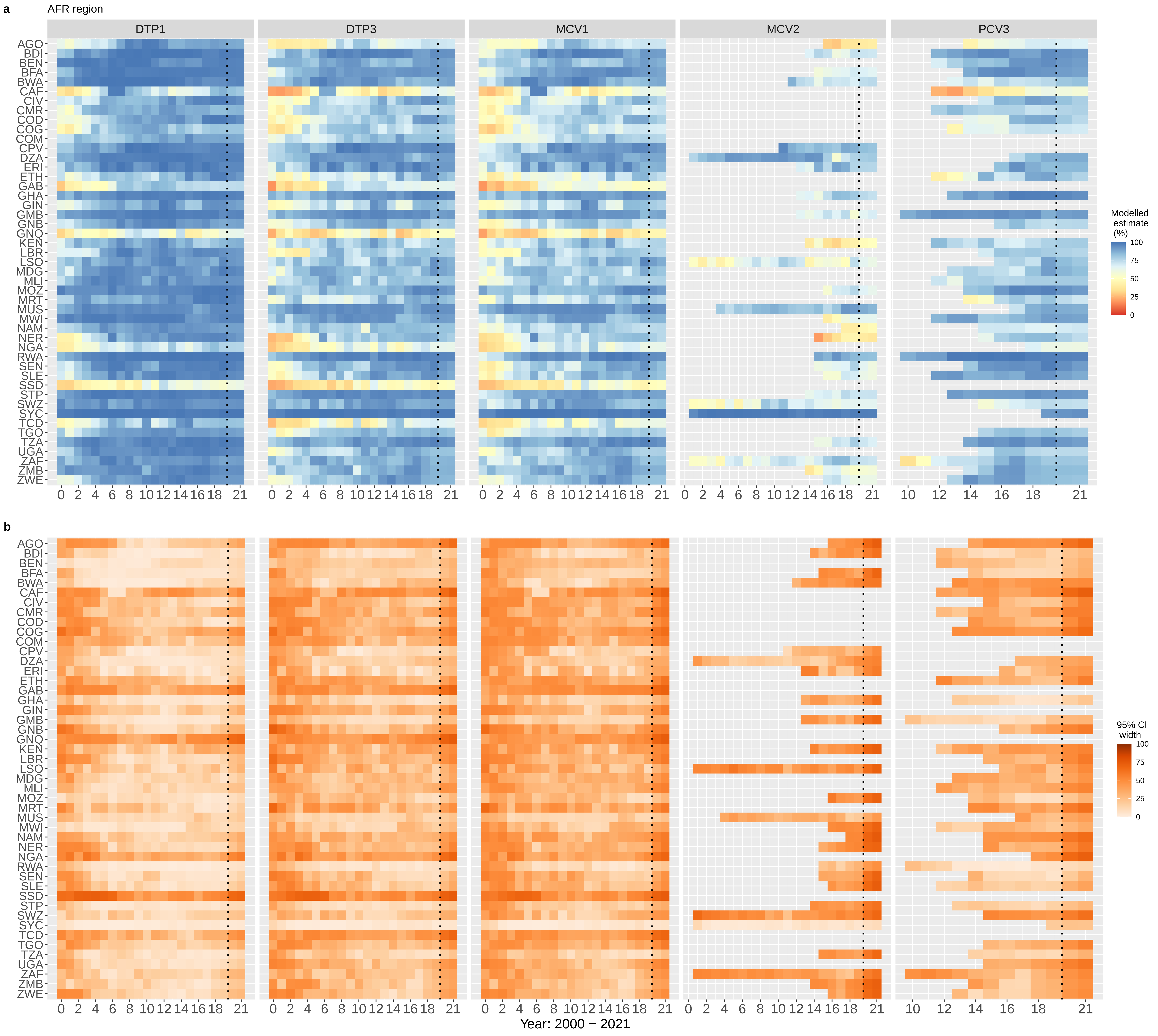
a
AFR region
DTP1
DTP3
MCV1
MCV2
PCV3
AGO
BDI
BEN
BFA
BWA
CAF
CIV
CMR
COD
COG
COM
CPV
DZA
ERI
ETH
GAB
GHA
GIN
GMB
GNB
GNQ
KEN
LBR
LSO
MDG
MLI
MOZ
MRT
MUS
MWI
NAM
NER
NGA
RWA
SEN
SLE
SSD
STP
SWZ
SYC
TCD
TGO
TZA
UGA
ZAF
ZMB
ZWE
0 2 4 6 8 10 12 14 16 18 21
10 12 14 16 18 21
Modelled estimate (%)
100
75
50
25
0
b
95% CI width
100
75
50
25
0
Year: 2000 − 2021
