# Supplementary material for: Bayesian Hierarchical Modeling Approaches for Combining Information From Multiple Data Sources to Produce Annual Estimates of National Immunization Coverage
Source: Biom J. 2026 Oct 6;68(5):e70189. doi: 10.1002/bimj.70189 (PMC13652340; doi:10.1002/bimj.70189)
Supplement: Supplementary file 2 [file BIMJ-68-e70189-s002.zip › code_and_data/outputs/Figure_S14.pdf]

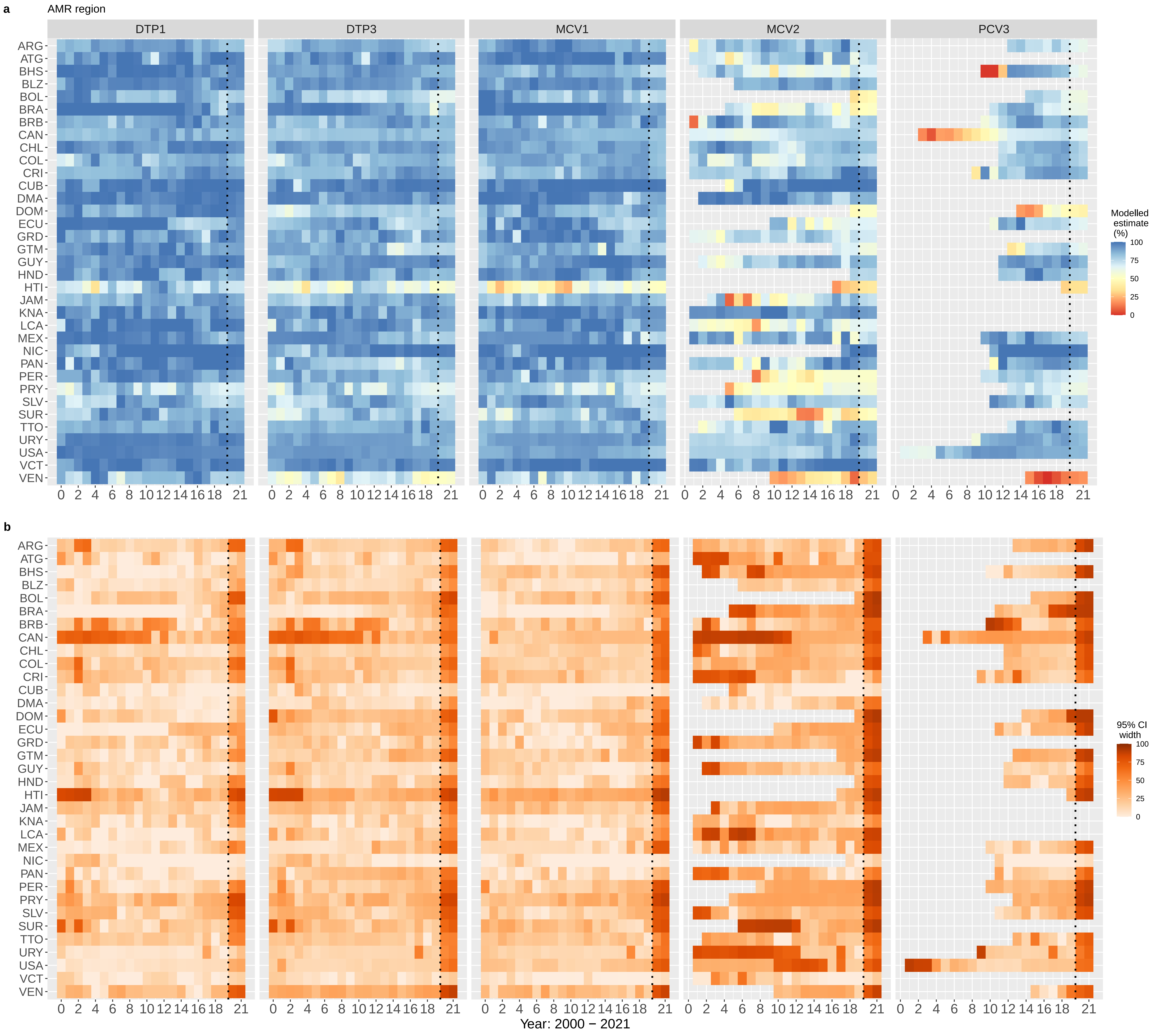

a
AMR region
DTP1
DTP3
MCV1
MCV2
PCV3
ARG
ATG
BHS
BLZ
BOL
BRA
BRB
CAN
CHL
COL
CRI
CUB
DMA
DOM
ECU
GRD
GTM
GUY
HND
HTI
JAM
KNA
LCA
MEX
NIC
PAN
PER
PRY
SLV
SUR
TTO
URY
USA
VCT
VEN
0 2 4 6 8 10 12 14 16 18 21
Modelled estimate (%)
100
75
50
25
0
b
95% CI width
100
75
50
25
0
Year: 2000 − 2021
