# Supplementary material for: Bayesian Hierarchical Modeling Approaches for Combining Information From Multiple Data Sources to Produce Annual Estimates of National Immunization Coverage
Source: Biom J. 2026 Oct 6;68(5):e70189. doi: 10.1002/bimj.70189 (PMC13652340; doi:10.1002/bimj.70189)
Supplement: Supplementary file 2 [file BIMJ-68-e70189-s002.zip › code_and_data/outputs/Figure_S17.pdf]

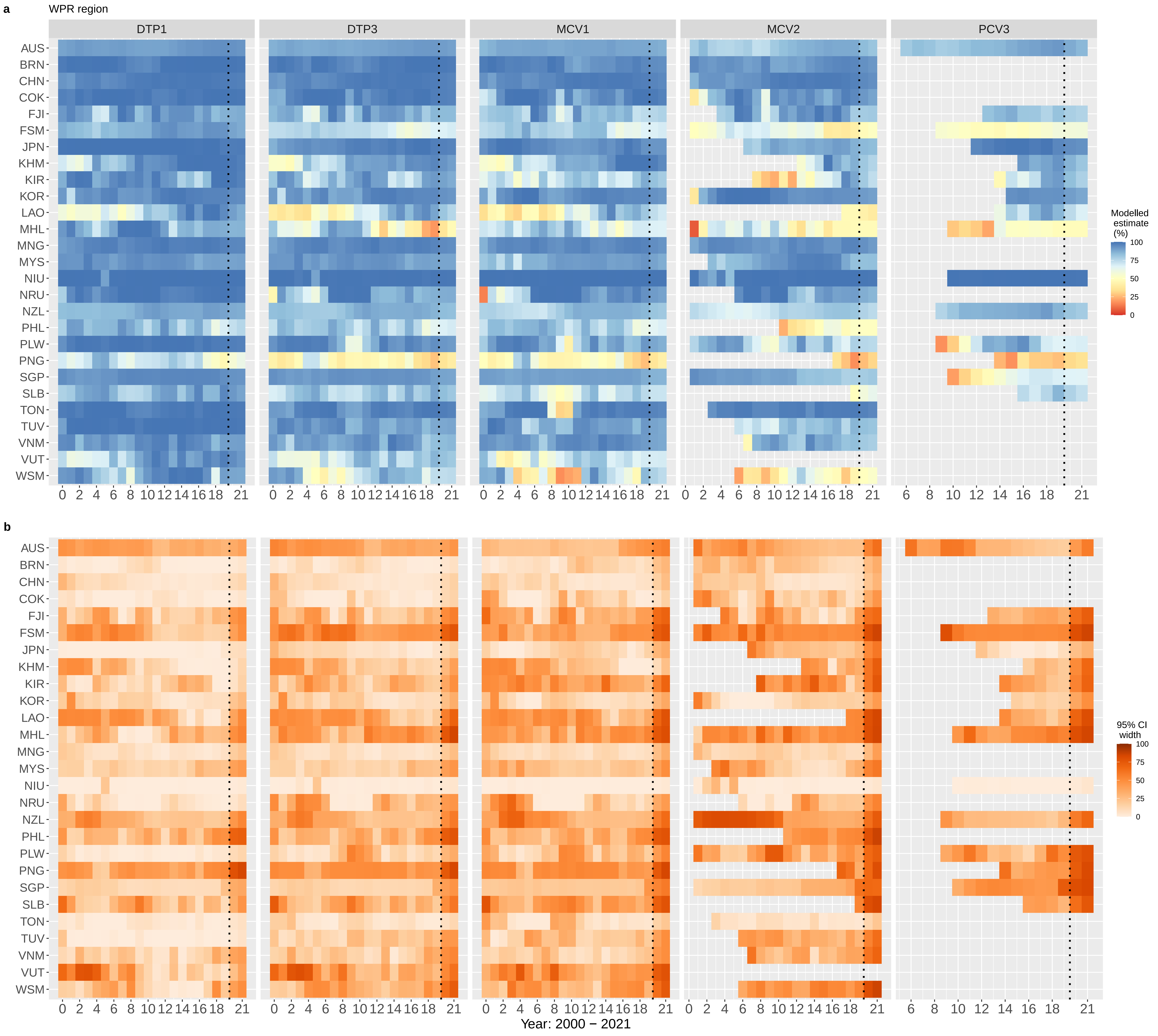

a
WPR region
DTP1
DTP3
MCV1
MCV2
PCV3
AUS
BRN
CHN
COK
FJI
FSM
JPN
KHM
KIR
KOR
LAO
MHL
MNG
MYS
NIU
NRU
NZL
PHL
PLW
PNG
SGP
SLB
TON
TUV
VNM
VUT
WSM
Modelled estimate (%)
100
75
50
25
0
b
95% CI width
100
75
50
25
0
0 2 4 6 8 10 12 14 16 18 21
6 8 10 12 14 16 18 21
Year: 2000 − 2021
