# Supplementary material for: Bayesian Hierarchical Modeling Approaches for Combining Information From Multiple Data Sources to Produce Annual Estimates of National Immunization Coverage
Source: Biom J. 2026 Oct 6;68(5):e70189. doi: 10.1002/bimj.70189 (PMC13652340; doi:10.1002/bimj.70189)
Supplement: Supplementary file 2 [file BIMJ-68-e70189-s002.zip › code_and_data/outputs/Figure_S18.pdf]

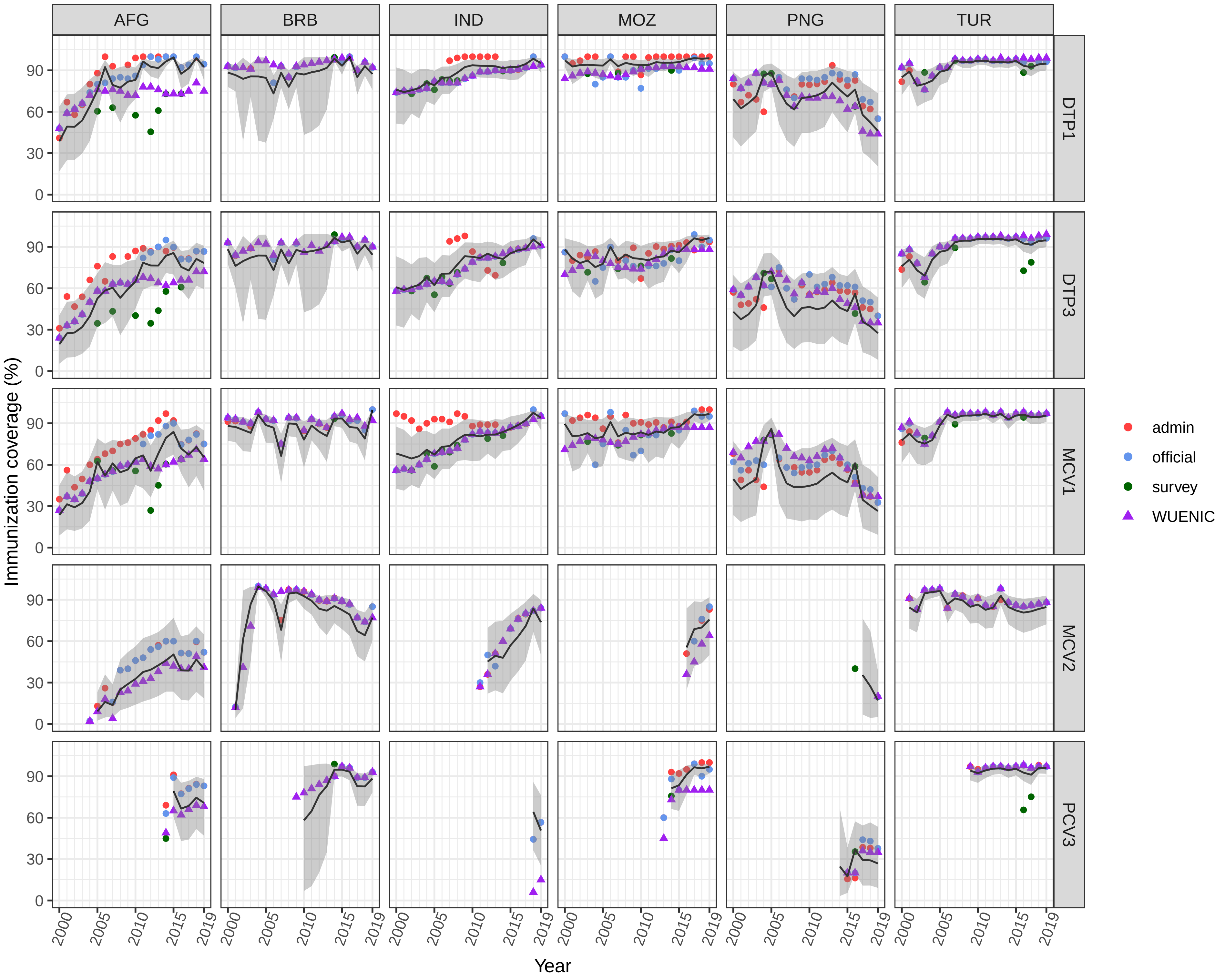
AFG
BRB
IND
MOZ
PNG
TUR
DTP1
DTP3
MCV1
MCV2
PCV3
Immunization coverage (%)
Year
0
30
60
90
2000
2005
2010
2015
2019
admin
official
survey
WUENIC
