# Supplementary material for: Bayesian Hierarchical Modeling Approaches for Combining Information From Multiple Data Sources to Produce Annual Estimates of National Immunization Coverage
Source: Biom J. 2026 Oct 6;68(5):e70189. doi: 10.1002/bimj.70189 (PMC13652340; doi:10.1002/bimj.70189)
Supplement: Supplementary file 2 [file BIMJ-68-e70189-s002.zip › code_and_data/outputs/Figure_S23.pdf]

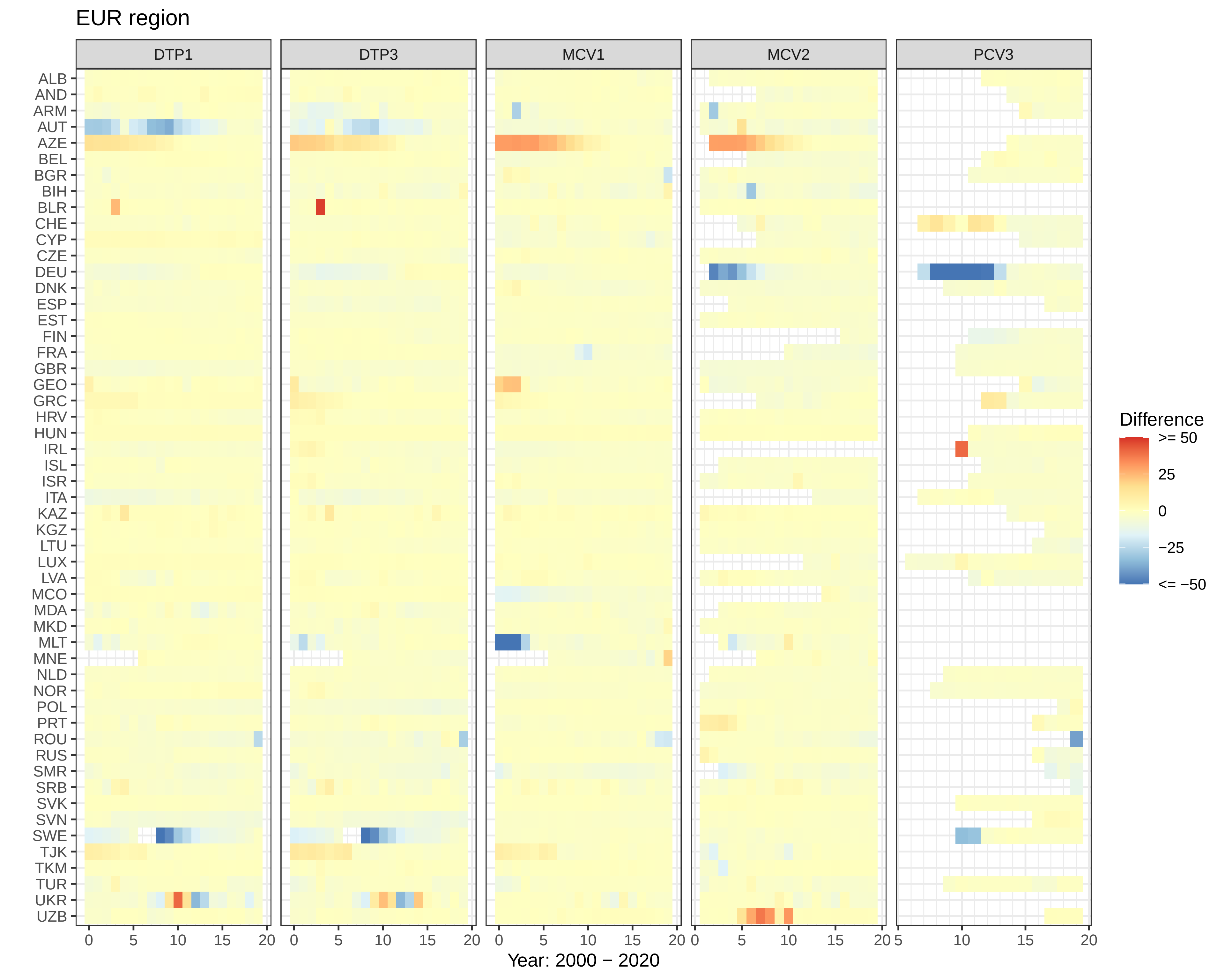
EUR region
DTP1
DTP3
MCV1
MCV2
PCV3
ALB
AND
ARM
AUT
AZE
BEL
BGR
BIH
BLR
CHE
CYP
CZE
DEU
DNK
ESP
EST
FIN
FRA
GBR
GEO
GRC
HRV
HUN
IRL
ISL
ISR
ITA
KAZ
KGZ
LTU
LUX
LVA
MCO
MDA
MKD
MLT
MNE
NLD
NOR
POL
PRT
ROU
RUS
SMR
SRB
SVK
SVN
SWE
TJK
TKM
TUR
UKR
UZB
0
5
10
15
20
Year: 2000 − 2020
Difference
>= 50
25
0
−25
<= −50
