# Supplementary material for: Bayesian Hierarchical Modeling Approaches for Combining Information From Multiple Data Sources to Produce Annual Estimates of National Immunization Coverage
Source: Biom J. 2026 Oct 6;68(5):e70189. doi: 10.1002/bimj.70189 (PMC13652340; doi:10.1002/bimj.70189)
Supplement: Supplementary file 2 [file BIMJ-68-e70189-s002.zip › code_and_data/outputs/Figure_S24.pdf]

SEAR region

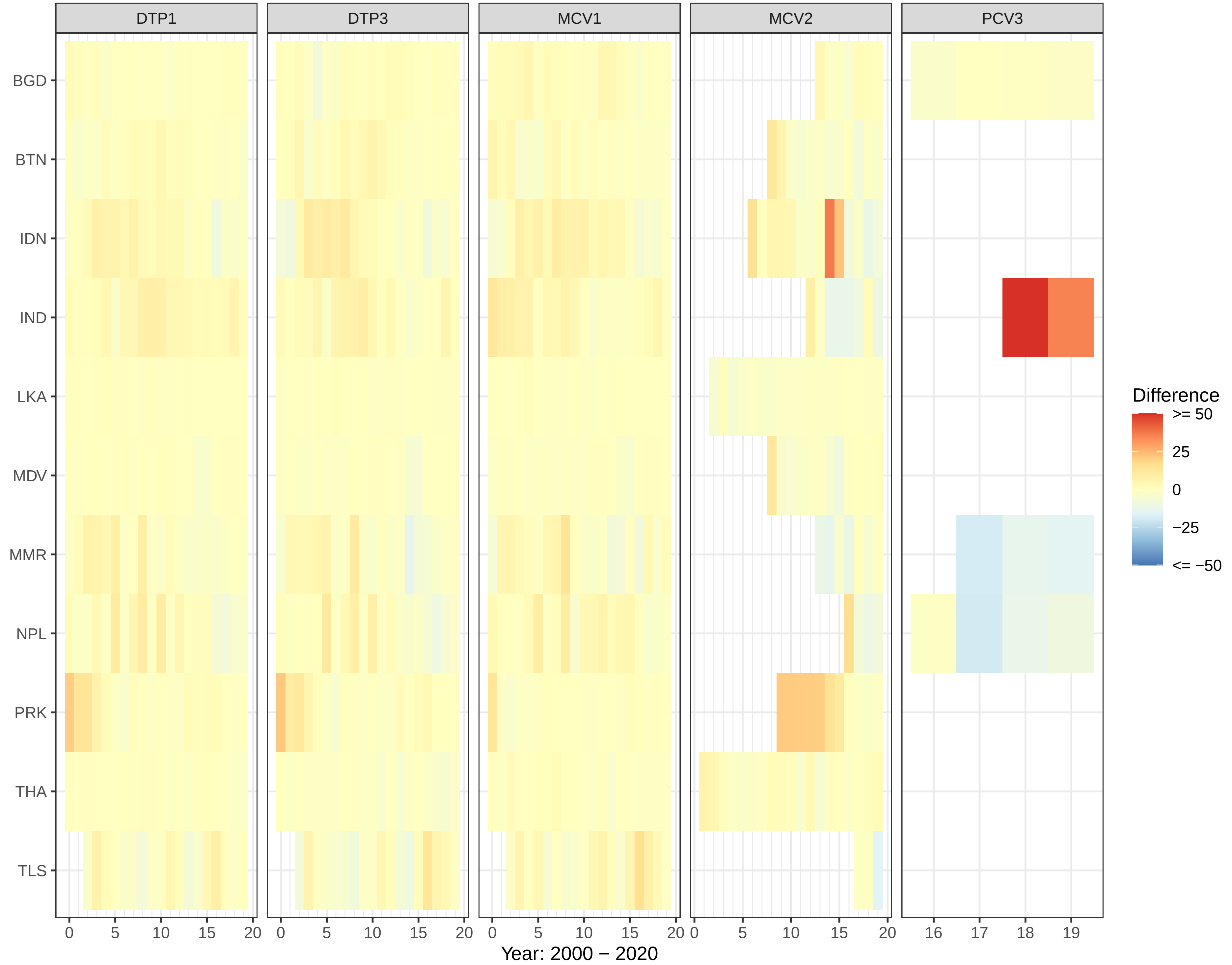
DTP1
DTP3
MCV1
MCV2
PCV3
BGD
BTN
IDN
IND
LKA
MDV
MMR
NPL
PRK
THA
TLS
0
5
10
15
20
16
17
18
19
Year: 2000 − 2020
Difference
>= 50
25
0
−25
<= −50
