# Supplementary material for: Bayesian Hierarchical Modeling Approaches for Combining Information From Multiple Data Sources to Produce Annual Estimates of National Immunization Coverage
Source: Biom J. 2026 Oct 6;68(5):e70189. doi: 10.1002/bimj.70189 (PMC13652340; doi:10.1002/bimj.70189)
Supplement: Supplementary file 2 [file BIMJ-68-e70189-s002.zip › code_and_data/outputs/Figure_S6.pdf]

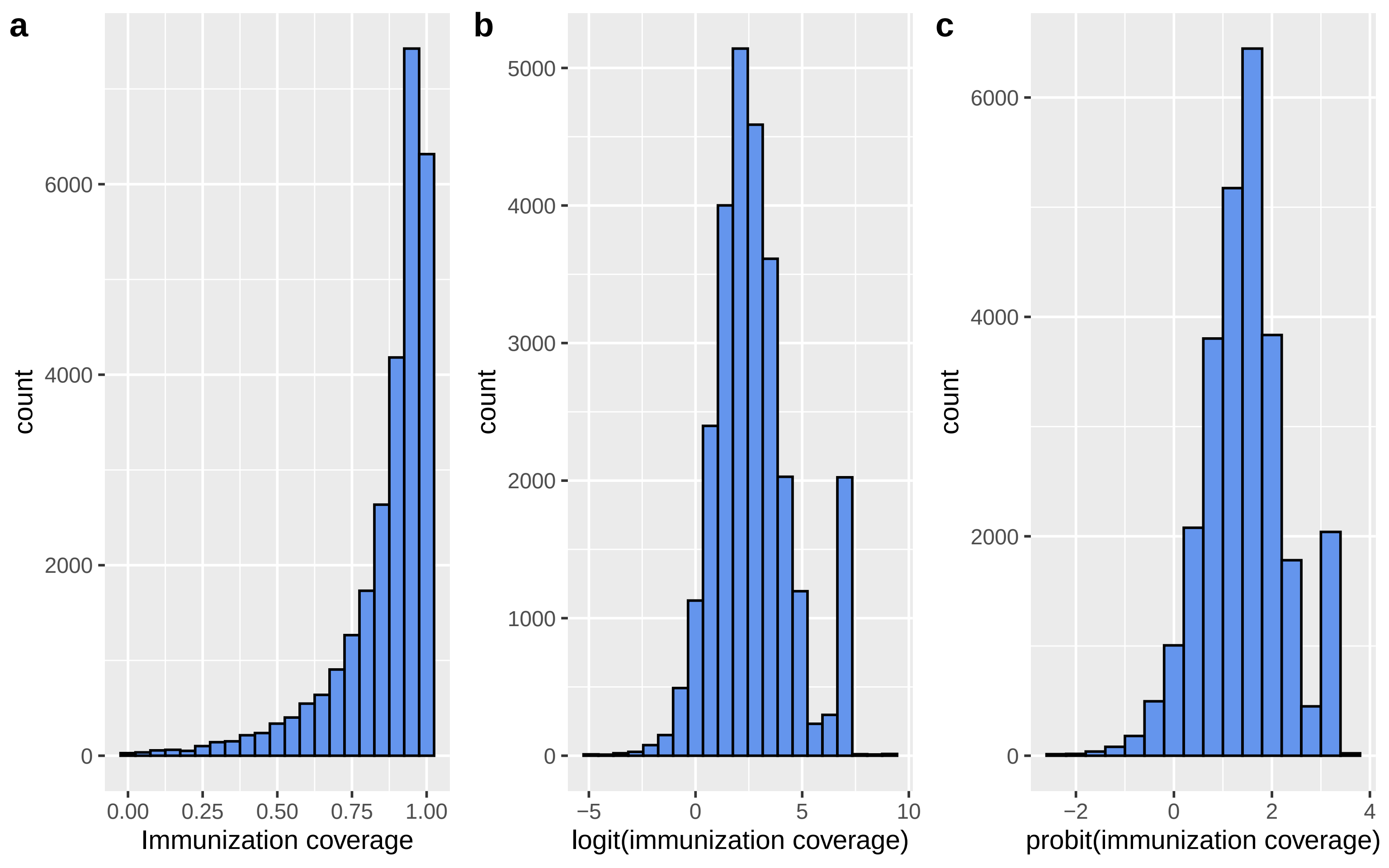

a
b
c
count
6000
4000
2000
0
0.00
0.25
0.50
0.75
1.00
Immunization coverage
5000
4000
3000
2000
1000
0
−5
0
5
10
logit(immunization coverage)
6000
4000
2000
0
−2
0
2
4
probit(immunization coverage)
