# Supplementary material for: Bayesian Hierarchical Modeling Approaches for Combining Information From Multiple Data Sources to Produce Annual Estimates of National Immunization Coverage
Source: Biom J. 2026 Oct 6;68(5):e70189. doi: 10.1002/bimj.70189 (PMC13652340; doi:10.1002/bimj.70189)
Supplement: Supplementary file 2 [file BIMJ-68-e70189-s002.zip › code_and_data/imcover/imcover_0_2_5.zip!/imcover-main/docs/articles/intro.html]

Model-based approaches to immunization coverage estimations in `R`: An introduction to the `imcover` package • imcover
 


  


 


Toggle navigation


imcover
0.2.4

- Reference
- Articles
  - Model-based approaches to immunization coverage estimations in `R`: An introduction to the `imcover` package
- Changelog

# Model-based approaches to immunization coverage estimations in `R`: An introduction to the `imcover` package

#### Chris Jochem (w.c.jochem@soton.ac.uk)

#### May 16, 2022

`intro.Rmd`

National estimates of immunization coverage are crucial for monitoring and evaluating coverage levels and trends, as well as immunization goals and targets, at the national and international levels. There are multiple potential sources of information related to immunization coverage, and the challenge is to develop a statistically-principled method to make use of different data sources while accounting for their uncertainties.

The goal of the `imcover` package is to provide access to data download, processing and modelling tools and to support a Bayesian statistical modelling approach to generate national estimates of immunization coverage from multiple time series of data on coverage. `imcover` is built as part of the open-source statistical computing and modelling language, `R` (https://cran.r-project.org/). This package is designed to support broadly replicable and reproducible analyses of immunization coverage.

This vignette will demonstrate some of the core functionality in the package, including:

- Downloading input data sources on coverage
- Cleaning/processing/harmonizing data sources
- Fitting a Bayesian statistical model and model outputs
- Generating national coverage estimates and visualizing results

### Installation and set-up

`imcover` is not (yet) available for download from CRAN. It is currently in a private GitHub repository during development. Access can be granted to users for “beta testing.” Once downloaded, a user accesses the code library through `R`.

```
# load the package within the R environment
library(imcover)
```

All functions are documented and the help and examples can be accessed within `R` using the standard command `help` or `?`.

`imcover` was developed by WorldPop at the University of Southampton (https://www.worldpop.org/).

## Accessing and downloading data

If a user does not have a dataset on their local computer, the first step in analysis is to acquire standardised data. Within `imcover` there is functionality to download several datasets from the WHO Immunization data portal (https://immunizationdata.who.int/listing.html). These datasets include official coverage reports, administrative records, as well as household survey datasets.

```
# download administrative and official records
cov <- download_coverage()

# download survey records
svy <- download_survey()
```

As part of the download process, by default, several pre-processing steps are done to, e.g. reconcile differences in column names. In the case of survey datasets, a recall-bias adjustment is applied. The default processing behaviour can be changed by modifying the argument supplied to the download functions. Additionally, all the processing steps can be carried out using functions available in `imcover` if advanced users want more control over pre-processing.

The data files available from the WHO website require some additional cleaning before analysis. Notice that the data objects created by `imcover` can work with all standard `R` commands for selecting and modifying records.

```
# Further data cleaning of immunization records
# drop some record categories (PAB, HPV, WUENIC)
cov <- cov[cov$coverage_category %in% c("ADMIN", "OFFICIAL"), ]
cov$coverage_category <- tolower(cov$coverage_category)  # clean-up

# create a combined dataset
dat <- rbind(cov, svy)

# remove records with missing coverage values
dat <- dat[!is.na(dat$coverage), ]

# mismatch in vaccine names between coverage and survey datasets
dat[dat$antigen == 'DTPCV1', 'antigen'] <- 'DTP1'
dat[dat$antigen == 'DTPCV2', 'antigen'] <- 'DTP2'
dat[dat$antigen == 'DTPCV3', 'antigen'] <- 'DTP3'
```

## `ic` data objects

A unique feature of `imcover` is that it creates and works with a custom data extension, which we refer to as an immunization coverage data frame or an `ic.df`. This type of object has all the functionality of a standard `R` data frame while having additional functionality to store attributes to make it easier to work with the immunization coverage data and to keep track of what processing has been done to the data.

```
# inspect a random sample of records
print(dat[sample(1:nrow(dat), size = 6), ])
#>        region code year antigen coverage coverage_category  doses target_number
#> 16396    EURO  AZE 2020    DTP3     79.0             admin  99582        126055
#> 166906   <NA>  MAC 1999 TT2PLUS     77.7          official     NA            NA
#> 228713   <NA>  PSE 2019    MCV2    100.0          official     NA            NA
#> 174780   AMRO  MEX 2017    POL3     85.0          official     NA            NA
#> 195704   AFRO  NAM 2017   HEPB3     88.0             admin  73431         83869
#> 222438   EURO  POL 2018   TTCV4     89.7             admin 341849        381193
#>                                                            name
#> 16396                                                Azerbaijan
#> 166906                                         China, Macao SAR
#> 228713 occupied Palestinian territory, including east Jerusalem
#> 174780                                                   Mexico
#> 195704                                                  Namibia
#> 222438                                                   Poland
#>                                                antigen_description
#> 16396                             DTP-containing vaccine, 3rd dose
#> 166906 Tetanus toxoid-containing vaccine, 2nd and subsequent doses
#> 228713                        Measles-containing vaccine, 2nd dose
#> 174780                                             Polio, 3rd dose
#> 195704                                                       HepB3
#> 222438          Tetanus-containing vaccine, 4th dose (1st booster)
#>        coverage_category_description surveyNameEnglish evidence validity
#> 16396        Administrative coverage              <NA>     <NA>     <NA>
#> 166906             Official coverage              <NA>     <NA>     <NA>
#> 228713             Official coverage              <NA>     <NA>     <NA>
#> 174780             Official coverage              <NA>     <NA>     <NA>
#> 195704       Administrative coverage              <NA>     <NA>     <NA>
#> 222438       Administrative coverage              <NA>     <NA>     <NA>
#>        Sample_Size surveyNameProduction collectBegin collectEnd cardsSeen
#> 16396           NA                 <NA>         <NA>       <NA>        NA
#> 166906          NA                 <NA>         <NA>       <NA>        NA
#> 228713          NA                 <NA>         <NA>       <NA>        NA
#> 174780          NA                 <NA>         <NA>       <NA>        NA
#> 195704          NA                 <NA>         <NA>       <NA>        NA
#> 222438          NA                 <NA>         <NA>       <NA>        NA
#>        ageVaccination ageInterview  sex adj_factor coverage_adj
#> 16396            <NA>         <NA> <NA>         NA           NA
#> 166906           <NA>         <NA> <NA>         NA           NA
#> 228713           <NA>         <NA> <NA>         NA           NA
#> 174780           <NA>         <NA> <NA>         NA           NA
#> 195704           <NA>         <NA> <NA>         NA           NA
#> 222438           <NA>         <NA> <NA>         NA           NA
```

```
# note the type object
class(dat)
#> [1] "ic.df"      "data.frame"
```

For example, the `ic.df` objects have helper functions to tabulate records,

```
# show all records
list_vaccines(dat)  # note the download files contain many vaccine
#>   [1] "BCG"                              "BCG-POST1DAY"                    
#>   [3] "BCG-WIN1DAY"                      "DIPHCV4"                         
#>   [5] "DIPHCV5"                          "DIPHCV6"                         
#>   [7] "DTP1"                             "DTP2"                            
#>   [9] "DTP3"                             "DTP4"                            
#>  [11] "FIPV1"                            "FIPV2"                           
#>  [13] "FLU_ALL"                          "FLU_CHILD1"                      
#>  [15] "FLU_CHILD2"                       "FLU_CHRONIC_ADULT"               
#>  [17] "FLU_CHRONIC_ALL"                  "FLU_CHRONIC_PED"                 
#>  [19] "FLU_ELDERLY"                      "FLU_HAJ"                         
#>  [21] "FLU_HCW"                          "FLU_OTHER"                       
#>  [23] "FLU_PW"                           "FLU_RESIDENT"                    
#>  [25] "FULL"                             "FULL (+HB)"                      
#>  [27] "FULL (+IPV +PENTA-POL-BOOSTER)"   "FULL (+IPV+YFV)"                 
#>  [29] "FULL (+PCV3)"                     "FULL (+ROTAC)"                   
#>  [31] "FULL (+YFV)"                      "FULL (ALL EXCL PCV AND BOOSTERS)"
#>  [33] "FULL (ALL EXCL PCV)"              "FULL <1 YEAR"                    
#>  [35] "FULL(+PCV)"                       "FULL(+PCV3+YFV)"                 
#>  [37] "FULL(+ROTAC)"                     "FULL(+YF,+HEPB)"                 
#>  [39] "FULL(+YF,+PCV,+ROTAC)"            "FULL(+YFV)"                      
#>  [41] "HEPB_BD"                          "HEPB_BDALL"                      
#>  [43] "HEPB0"                            "HEPB1"                           
#>  [45] "HEPB2"                            "HEPB3"                           
#>  [47] "HEPB4"                            "HEPBB"                           
#>  [49] "HEPBBX"                           "HIB1"                            
#>  [51] "HIB2"                             "HIB3"                            
#>  [53] "HIB4"                             "HPV_FEM"                         
#>  [55] "HPV_MALE"                         "IPV"                             
#>  [57] "IPV1"                             "IPV1_FRAC"                       
#>  [59] "IPV1X"                            "IPV2"                            
#>  [61] "IPV2_FRAC"                        "IPV3"                            
#>  [63] "IPV4"                             "JAPENC"                          
#>  [65] "JAPENC_1"                         "JAPENC_C"                        
#>  [67] "MCV1"                             "MCV2"                            
#>  [69] "MCV2X"                            "MCV3"                            
#>  [71] "MEN_A_CONJ"                       "NONE"                            
#>  [73] "OPV"                              "PAB"                             
#>  [75] "PCV1"                             "PCV2"                            
#>  [77] "PCV3"                             "PCV4"                            
#>  [79] "PERCV_PW"                         "PERCV4"                          
#>  [81] "POL0"                             "POL1"                            
#>  [83] "POL1X"                            "POL2"                            
#>  [85] "POL3"                             "POL3+IPV1"                       
#>  [87] "POL4"                             "POL4+IPV1"                       
#>  [89] "RCV1"                             "RCV1X"                           
#>  [91] "RCV2"                             "RCV2X"                           
#>  [93] "RCV3"                             "ROTA1"                           
#>  [95] "ROTA2"                            "ROTAC"                           
#>  [97] "ROTACX"                           "TT1"                             
#>  [99] "TT1+"                             "TT2"                             
#> [101] "TT2+"                             "TT2PLUS"                         
#> [103] "TT3"                              "TT4"                             
#> [105] "TT5"                              "TTCV4"                           
#> [107] "TTCV5"                            "TTCV6"                           
#> [109] "TTT5"                             "TTTT10"                          
#> [111] "TTTTT+"                           "TYPHOID"                         
#> [113] "TYPHOID_CONJ"                     "VAD1"                            
#> [115] "YFV"
```

Or to easily filter the records. Here we will subset the data for five core vaccines and a subset of time points.

```
# subset the records by vaccine and year for all countries
dat <- ic_filter(dat, 
                 vaccine = c("DTP1", "DTP3", "MCV1", "MCV2", "PCV3"),
                 time = 2000:2018)

# cross-tabulations of source and vaccine
table(dat$coverage_category, dat$antigen)
#>           
#>            DTP1 DTP3 MCV1 MCV2 PCV3
#>   admin    3020 3376 3320 2056  936
#>   official 3061 3508 3489 1997  967
#>   survey    594  643  637   56  163
```

Other specialised methods are available to filter out records for a country after the year of national introduction.

```
# subset records based on year of vaccine introduction
dat <- filter_yovi(dat)
  table(dat$year, dat$antigen)  # notice year of introduction for PCV3
#>       
#>        DTP1 DTP3 MCV1 MCV2 PCV3
#>   2000  310  377  353   14    0
#>   2001  291  353  345  112    0
#>   2002  281  350  340  112    0
#>   2003  302  378  368  127    0
#>   2004  343  396  398  148    2
#>   2005  369  420  416  166    2
#>   2006  341  385  384  166    3
#>   2007  372  419  421  190    8
#>   2008  351  391  385  187   12
#>   2009  359  401  398  200   28
#>   2010  357  402  398  207   68
#>   2011  374  411  408  215   89
#>   2012  373  416  414  231  136
#>   2013  363  394  394  243  165
#>   2014  365  398  393  254  194
#>   2015  367  398  397  286  234
#>   2016  378  411  408  301  261
#>   2017  396  421  420  322  287
#>   2018  383  406  406  336  283
```

Some further pre-processing of immunization data has been found to be necessary in preparation for the statistical modelling. We introduce functions to make it easy to carry out these steps.

Firstly, some records observe inconsistencies in the levels of coverage between multi-dose vaccines. To maintain consistency, where coverage of later doses cannot exceed earlier doses, we model the ratio between first and third dose. In this example, we only adjust DTP1 and DTP3, but other multi-dose vaccines could be processed in a similar manner.

```
# adjustment - use ratio for DTP3
dat <- ic_ratio(dat, numerator = 'DTP3', denominator = 'DTP1')
```

The `ic.df` object will now store a note that this processing step has been carried out so that the ratio is back-transformed and that coverage estimates and predictions are adjusted appropriately.

Secondly, we need to force coverage estimates to lie between 0% and 100%.

```
# maintain coverage between 0-100%
dat <- ic_adjust(dat, coverage_adj = TRUE)
```

## Model fitting

The core of `imcover` is the functionality to fit a Bayesian statistical model of multiple time series. The sources of coverage data (in this example administrative, official and surveys) are taken as multiple, partial estimates of the true, unobserved immunization coverage in a country. A Bayesian estimation approach allows us to incorporate these multiple datasets, place prior beliefs on which sources are more reliable, share information between countries, and to quantify uncertainty in our estimate of the latent immunization coverage.

`imcover` provides an interface to `Stan` (https://mc-stan.org/) for statistical computation. This means that, in addition to `imcover`, many of the tools for assessing model performance and visualizing results from `Stan` will work for `imcover` results.

In this demonstration will start by fitting a model to a single region of data using `ic_fit` by passing in the `ic.df` object and several arguments that control the posterior sampling.

```
options(mc.cores = parallel::detectCores())  # enable parallel processing

# Fit model to a single region of data
fit1 <- ic_fit(dat[dat$region == 'AFRO', ], 
               chains = 4, 
               iter = 2000, 
               warmup = 1000)
```

### Model outputs

After fitting the model, we can work with the parameter estimates and estimates of coverage. For example, we can easily make graphs of coverage estimates. Notice how `imcover` has a generic function defined to specially handle objects returned by `ic_fit`.

```
# Plot of coverage
plot(ic_filter(fit1, vaccine = 'DTP1',
               country = c('BWA', 'SLE', 'GHA', 'NGA')),
     ncol = 2)
```

```
plot(ic_filter(fit1, vaccine = 'DTP3',
               country = c('BWA', 'SLE', 'GHA', 'NGA')),
     ncol = 2)
```

Example results of modelled immunization coverage estimates for selected countries and vaccines.

The main output of the model is a table of coverage estimates for each country, vaccine, and time point along with uncertainty around each estimate. We can extract these data as a table, save them to a data frame, or write them out to a file for use in a report.

```
# Extracting coverage estimates
ic <- ic_coverage(fit1, 
                  stat = "quantile", # customise the summary function
                  probs = c(0.025, 0.5, 0.975))

head(ic, 10)
#>        country time vaccine     2.5%      50%    97.5%
#> mu[1]      AGO 2000    DTP1 43.16206 65.09245 79.41213
#> mu[2]      AGO 2001    DTP1 34.13583 59.68096 82.58923
#> mu[3]      AGO 2002    DTP1 40.38493 66.99820 87.66832
#> mu[4]      AGO 2003    DTP1 42.76260 68.12311 87.75516
#> mu[5]      AGO 2004    DTP1 46.78410 72.83099 89.71176
#> mu[6]      AGO 2005    DTP1 45.44075 72.14182 87.56244
#> mu[7]      AGO 2006    DTP1 57.28637 78.51181 90.14268
#> mu[8]      AGO 2007    DTP1 75.84889 88.43659 93.76432
#> mu[9]      AGO 2008    DTP1 91.22958 96.79864 98.80278
#> mu[10]     AGO 2009    DTP1 83.42451 94.24143 97.89507
```

### Predictions

Once we have the fitted model, we can also use it to predict coverage to time points in the near future.

```
# Predict for future time points (3 years post 2018)
fit1 <- predict(fit1, t = 3)  # update icfit to include 'prediction' info

# Update the graph 
# Automatically include fitted estimates, observed data, and predictions
plot(ic_filter(fit1, vaccine = 'DTP1',
               country = c('BWA', 'SLE', 'GHA', 'NGA')),
     ncol = 2)
```

```
plot(ic_filter(fit1, vaccine = 'DTP3',
               country = c('BWA', 'SLE', 'GHA', 'NGA')),
     ncol = 2)
```

Example reults of estimated immunization coverage with future predictions to the right of the dashed lines. Overlaid with observed data points.

## Summary

This vignette has introduced the core functionality of the `imcover` package and its use in modelling national time series of immunization coverage. Providing a coordinated toolset to download, process, and model immunization data should allow for consistent analyses whose methods are transparent and replicable.

There are a few additional options in the package. For instance, multiple regions of data can be supplied to `ic_fit` and then region-specific models can be executed. In the posterior analyses, population-weighted regional summaries of coverage can also be calculated. Similar to `ic_coverage`, users can customize the summary functions. Finally, the `Stan` models allow for significant flexibility in defining statistical models. The package specifies reasonable default values for the models to replicate our analyses, but users can explore the sensitivity of these settings by easily adjusting the parameters within `R` and re-running the models without having to interact with the `Stan` code.

Feedback on the package is welcome. Please email Chris Jochem (w.c.jochem@soton.ac.uk)

---

```
#> R version 4.1.3 (2022-03-10)
#> Platform: x86_64-pc-linux-gnu (64-bit)
#> Running under: Ubuntu 20.04.4 LTS
#> 
#> Matrix products: default
#> BLAS:   /usr/lib/x86_64-linux-gnu/blas/libblas.so.3.9.0
#> LAPACK: /usr/lib/x86_64-linux-gnu/lapack/liblapack.so.3.9.0
#> 
#> locale:
#>  [1] LC_CTYPE=en_GB.UTF-8       LC_NUMERIC=C              
#>  [3] LC_TIME=en_GB.UTF-8        LC_COLLATE=en_GB.UTF-8    
#>  [5] LC_MONETARY=en_GB.UTF-8    LC_MESSAGES=en_GB.UTF-8   
#>  [7] LC_PAPER=en_GB.UTF-8       LC_NAME=C                 
#>  [9] LC_ADDRESS=C               LC_TELEPHONE=C            
#> [11] LC_MEASUREMENT=en_GB.UTF-8 LC_IDENTIFICATION=C       
#> 
#> attached base packages:
#> [1] stats     graphics  grDevices utils     datasets  methods   base     
#> 
#> other attached packages:
#> [1] imcover_0.2.4
#> 
#> loaded via a namespace (and not attached):
#>  [1] Rcpp_1.0.7           prettyunits_1.1.1    ps_1.6.0            
#>  [4] rprojroot_2.0.2      digest_0.6.29        utf8_1.2.2          
#>  [7] V8_3.4.2             cellranger_1.1.0     R6_2.5.1            
#> [10] stats4_4.1.3         evaluate_0.15        highr_0.9           
#> [13] ggplot2_3.3.5        pillar_1.7.0         rlang_1.0.2         
#> [16] readxl_1.3.1         curl_4.3.2           rstudioapi_0.13     
#> [19] callr_3.7.0          jquerylib_0.1.3      rmarkdown_2.13      
#> [22] pkgdown_1.6.1        labeling_0.4.2       textshaping_0.3.5   
#> [25] desc_1.4.0           stringr_1.4.0        loo_2.5.1           
#> [28] munsell_0.5.0        compiler_4.1.3       xfun_0.30           
#> [31] rstan_2.21.2         pkgconfig_2.0.3      systemfonts_1.0.4   
#> [34] pkgbuild_1.3.1       rstantools_2.1.1     htmltools_0.5.2     
#> [37] tidyselect_1.1.0     tibble_3.1.6         gridExtra_2.3       
#> [40] codetools_0.2-18     matrixStats_0.61.0   fansi_1.0.3         
#> [43] crayon_1.5.1         dplyr_1.0.5          withr_2.5.0         
#> [46] grid_4.1.3           jsonlite_1.8.0       gtable_0.3.0        
#> [49] lifecycle_1.0.1      magrittr_2.0.3       StanHeaders_2.21.0-7
#> [52] scales_1.1.1         RcppParallel_5.1.5   cli_3.2.0           
#> [55] stringi_1.7.6        cachem_1.0.6         farver_2.1.0        
#> [58] fs_1.5.2             bslib_0.2.4          ellipsis_0.3.2      
#> [61] ragg_1.1.3           generics_0.1.0       vctrs_0.4.0         
#> [64] tools_4.1.3          glue_1.6.2           purrr_0.3.4         
#> [67] abind_1.4-5          processx_3.5.3       parallel_4.1.3      
#> [70] fastmap_1.1.0        yaml_2.3.5           inline_0.3.19       
#> [73] colorspace_2.0-3     memoise_2.0.1        knitr_1.38          
#> [76] sass_0.3.1
```

## Contents

Developed by Warren C. Jochem, Edson Utazi, Sahu Sujit.

Site built with pkgdown 1.6.1.
