# Supplementary material for: Bayesian Hierarchical Modeling Approaches for Combining Information From Multiple Data Sources to Produce Annual Estimates of National Immunization Coverage
Source: Biom J. 2026 Oct 6;68(5):e70189. doi: 10.1002/bimj.70189 (PMC13652340; doi:10.1002/bimj.70189)
Supplement: Supplementary file 2 [file BIMJ-68-e70189-s002.zip › code_and_data/imcover/imcover_0_2_5.zip!/imcover-main/docs/index.html]

Spatio-temporal Modelling of Immunisation Coverage • imcover
 


  


 


Toggle navigation


imcover
0.2.4

- Reference
- Articles
  - Model-based approaches to immunization coverage estimations in `R`: An introduction to the `imcover` package
- Changelog

# imcover: Immunisation coverage modelling in R


The goal of the `imcover` package is to provide access to data download, processing and modelling tools to support a Bayesian statistical modelling approach to generate national estimates of immunization coverage from multiple time series of data on coverage. `imcover` is built as part of the open-source statistical computing and modelling language, `R` (https://cran.r-project.org/). This package is designed to support broadly replicable and reproducible analyses of immunization coverage.

## Statistical modelling software

The core of `imcover` is the functionality to fit a Bayesian statistical model of multiple time series. The sources of coverage data (in this example administrative, official and surveys) are taken as multiple, partial estimates of the true, unobserved immunization coverage in a country. A Bayesian estimation approach allows us to incorporate these multiple datasets, place prior beliefs on which sources are more reliable, share information between countries, and to quantify uncertainty in our estimate of the latent immunization coverage.

`imcover` provides an interface to `Stan` (https://mc-stan.org/) for statistical computation. This means that, in addition to `imcover`, many of the tools for assessing model performance and visualizing results from `Stan` will work for `imcover` results. However, users must have `Stan` installed and linked with `R` in order to use `imcover`

To install `Stan`, please follow the instructions for your operating system described here: https://github.com/stan-dev/rstan/wiki/RStan-Getting-Started

## Installing `imcover`

The `imcover` package is not yet on CRAN and can be installed from Github using the following command:

```
devtools::install_github('wpgp/imcover@main')
```

The build process takes some time because it is compiling the C++ code for the `Stan` models. It may also ask you to install some additional dependencies.

## Contributions

Feedback and contributions are welcome. Please raise or respond to an issue, or create a new branch to develop a feature/modification and submit a pull request.

## Acknowledements

This work was funded by WHO and carried out by members of the WorldPop project at the University of Southampton, United Kingdom. The authors gratefully acknowledge the WHO-UNICEF immunization coverage working group for their valuable inputs and feedback during model and software development.

## License

- Full license
- GPL (>= 3)

## Developers

- Warren C. Jochem   
   Author, maintainer
- Edson Utazi   
   Author
- Sahu Sujit   
   Author

Developed by Warren C. Jochem, Edson Utazi, Sahu Sujit.

Site built with pkgdown 1.6.1.
