# Supplementary material for: Bayesian Hierarchical Modeling Approaches for Combining Information From Multiple Data Sources to Produce Annual Estimates of National Immunization Coverage
Source: Biom J. 2026 Oct 6;68(5):e70189. doi: 10.1002/bimj.70189 (PMC13652340; doi:10.1002/bimj.70189)
Supplement: Supplementary file 2 [file BIMJ-68-e70189-s002.zip › code_and_data/imcover/imcover_0_2_5.zip!/imcover-main/docs/news/index.html]

Changelog • imcover


Toggle navigation


imcover
0.2.4

- Reference
- Articles
  - Model-based approaches to immunization coverage estimations in `R`: An introduction to the `imcover` package
- Changelog

# Changelog

# imcover 0.2.4

- Updated documentation
- Update to include the single-likelihood model.
- Corrected `predict` to back-transform ratio adjustments.

# imcover 0.2.3 (not released)

- Assorted bug fixes.

# imcover 0.2.2

- Bug fix in the multi-likelihood model.

# imcover 0.2.1

- Minor update to plotting allows overlay of WUENIC data.
- Changed the default x- and y-axis labels in plots.

# imcover 0.2.0

- Revised `Stan` model to allow variable number of sources.
- Added parameters to `ic_fit` for user-defined priors and parameter bounds.
- Added single-country versions of the model.

# imcover 0.1.0

- Version created for internal ‘beta’ testing
- Added a `NEWS.md` file to track changes to the package.

## Contents

Developed by Warren C. Jochem, Edson Utazi, Sahu Sujit.

Site built with pkgdown 1.6.1.
