# Supplementary material for: Bayesian Hierarchical Modeling Approaches for Combining Information From Multiple Data Sources to Produce Annual Estimates of National Immunization Coverage
Source: Biom J. 2026 Oct 6;68(5):e70189. doi: 10.1002/bimj.70189 (PMC13652340; doi:10.1002/bimj.70189)
Supplement: Supplementary file 2 [file BIMJ-68-e70189-s002.zip › code_and_data/imcover/imcover_0_2_5.zip!/imcover-main/docs/reference/as.ic_data.html]

Convert foreign object to an ic data object — as.ic\_data • imcover


Toggle navigation


imcover
0.2.4

- Reference
- Articles
  - Model-based approaches to immunization coverage estimations in `R`: An introduction to the `imcover` package
- Changelog

# Convert foreign object to an ic data object

`as.ic_data.Rd`

Functions to check if an object is an ic data object, or coerce
it to one if possible.

```
as.ic_data(...)

is.ic_data(object)
```

## Arguments

|  |  |
| --- | --- |
| ... | Elements to be combined into an `ic.df` object. |
| object | Any `R` object. |

## Value

An object of type `ic.df`.

## Details

Core `ic.df` data elements are expected. Specifically, columns
of data for 'region', 'country', 'time', 'vaccine', 'coverage', and
'source' (optionally 'dose' and 'population') are required in this order.
The names of these elements from `...` are passed to `ic_data`
and used along with the default settings.

## See also

`ic_data`

## Examples

```
if (FALSE) {
# convert data.frame to an imcover data frame
ic_df <- as.ic_data(df)
}
```

## Contents

Developed by Warren C. Jochem, Edson Utazi, Sahu Sujit.

Site built with pkgdown 1.6.1.
