# Supplementary material for: Bayesian Hierarchical Modeling Approaches for Combining Information From Multiple Data Sources to Produce Annual Estimates of National Immunization Coverage
Source: Biom J. 2026 Oct 6;68(5):e70189. doi: 10.1002/bimj.70189 (PMC13652340; doi:10.1002/bimj.70189)
Supplement: Supplementary file 2 [file BIMJ-68-e70189-s002.zip › code_and_data/imcover/imcover_0_2_5.zip!/imcover-main/docs/reference/download.html]

Download WUENIC data files — download • imcover


Toggle navigation


imcover
0.2.4

- Reference
- Articles
  - Model-based approaches to immunization coverage estimations in `R`: An introduction to the `imcover` package
- Changelog

# Download WUENIC data files

`download.Rd`

Retrieve the input data files and WUENIC coverage estimates with further
processing of `ic.df` objects.

```
download_wuenic(
  destfile,
  url,
  use_cache = TRUE,
  quiet = FALSE,
  attempts = 3,
  mode = "wb",
  return_ic = TRUE,
  add_region = "who",
  ...
)

download_coverage(
  destfile,
  url,
  use_cache = TRUE,
  quiet = FALSE,
  attempts = 3,
  mode = "wb",
  return_ic = TRUE,
  add_region = "who",
  ...
)

download_survey(
  destfile,
  url,
  use_cache = TRUE,
  quiet = FALSE,
  attempts = 3,
  mode = "wb",
  return_ic = TRUE,
  reduce = TRUE,
  minSample = 300,
  adjust = TRUE,
  adjVacc = c("DTP", "PCV"),
  add_region = "who",
  ...
)

download_denom(
  destfile,
  url,
  use_cache = TRUE,
  quiet = FALSE,
  attempts = 3,
  mode = "wb",
  ...
)
```

## Arguments

|  |  |
| --- | --- |
| destfile | Optional. Character string with the name of where the downloaded file is saved. |
| url | Optional. A character string naming the URL of the resource to download. Default is the .xlsx from the WHO immunization data website. |
| use\_cache | Logical. Look for an already downloaded file is the `destfile` location? Default is `TRUE`. |
| quiet | Should status messages and progress bars be hidden? Default is `FALSE`. |
| attempts | integer of the number of attempts to download the file. Default is 3. |
| mode | a character string specifying the write mode. 'wb' is most common to write binary files. |
| return\_ic | Should an object of type `ic.df` be returned? Default is `TRUE`. |
| add\_region | Optional. Character string for the type of region code to add based on the ISO3 code of the country. Options are: 'who' or 'm49'. |
| ... | additional arguments to be passed on to `download.file`. |
| reduce | For surveys only, should records with insufficient coverage be dropped? Default is `TRUE`. |
| minSample | For surveys only, what is the minimum sample size to keep survey records? Default is 300. |
| adjust | For survey only, should a recall-bias adjustment be applied? Default is `TRUE`. |
| adjVacc | Character vector of vaccines to adjust when `adjust` is `TRUE`. Default is "DTP" and "PCV". |

## Value

The functions return a `data.frame` while
`download_coverage` and `downlaod_survey` return objects of type
`ic.df` unless the option `return_ic` is `FALSE`.

## See also

`ic_data`

## Contents

Developed by Warren C. Jochem, Edson Utazi, Sahu Sujit.

Site built with pkgdown 1.6.1.
