# Supplementary material for: Bayesian Hierarchical Modeling Approaches for Combining Information From Multiple Data Sources to Produce Annual Estimates of National Immunization Coverage
Source: Biom J. 2026 Oct 6;68(5):e70189. doi: 10.1002/bimj.70189 (PMC13652340; doi:10.1002/bimj.70189)
Supplement: Supplementary file 2 [file BIMJ-68-e70189-s002.zip › code_and_data/imcover/imcover_0_2_5.zip!/imcover-main/docs/reference/filter_yovi.html]

Filter ic data records based on year of vaccine introduction
Exclude ic data records for years when a vaccine had not been fully
introduced in a country. — filter\_yovi • imcover


Toggle navigation


imcover
0.2.4

- Reference
- Articles
  - Model-based approaches to immunization coverage estimations in `R`: An introduction to the `imcover` package
- Changelog

# Filter ic data records based on year of vaccine introduction Exclude ic data records for years when a vaccine had not been fully introduced in a country.

`filter_yovi.Rd`

Filter ic data records based on year of vaccine introduction
Exclude ic data records for years when a vaccine had not been fully
introduced in a country.

```
filter_yovi(X, vaccine = NULL, yovi = imcover::get_yovi(), na.rm = FALSE)

# S3 method for ic.df
filter_yovi(X, vaccine = NULL, yovi = imcover::get_yovi(), na.rm = FALSE)

# S3 method for icfit
filter_yovi(X, vaccine = NULL, yovi = imcover::get_yovi(), na.rm = FALSE)

# S3 method for iclist
filter_yovi(X, vaccine = NULL, yovi = imcover::get_yovi(), na.rm = FALSE)
```

## Arguments

|  |  |
| --- | --- |
| X | An object of class `ic.df` or `icfit`. |
| vaccine | Vector of vaccine codes as characters. Default is `NULL` which includes all vaccines in object `X`. |
| yovi | Table of the year of vaccine introduction. See details. Default value comes from `imcover::get_yovi()`. |
| na.rm | Should records which do not have any data for year of introduction be removed? Default is `FALSE` to keep `NA` records in `X`. |

## Value

An object with type of `X` with records filtered by the year of
vaccine introductions.

## Details

The `yovi` argument can take a user-defined table of values.
This must be a `data.frame` object with column names 'ISO3code',
'vaccine' and 'year\_introduced'.

## See also

`get_yovi`

## Contents

Developed by Warren C. Jochem, Edson Utazi, Sahu Sujit.

Site built with pkgdown 1.6.1.
