# Supplementary material for: Bayesian Hierarchical Modeling Approaches for Combining Information From Multiple Data Sources to Produce Annual Estimates of National Immunization Coverage
Source: Biom J. 2026 Oct 6;68(5):e70189. doi: 10.1002/bimj.70189 (PMC13652340; doi:10.1002/bimj.70189)
Supplement: Supplementary file 2 [file BIMJ-68-e70189-s002.zip › code_and_data/imcover/imcover_0_2_5.zip!/imcover-main/docs/reference/get_attr.html]

Get the attributes of ic data
Retrieve the value(s) of attributes from an ic.df dataset. While
intended to be used with the core ic attributes, it will work with any named
attribute. — get\_attr • imcover


Toggle navigation


imcover
0.2.4

- Reference
- Articles
  - Model-based approaches to immunization coverage estimations in `R`: An introduction to the `imcover` package
- Changelog

# Get the attributes of ic data Retrieve the value(s) of attributes from an `ic.df` dataset. While intended to be used with the core ic attributes, it will work with any named attribute.

`get_attr.Rd`

Get the attributes of ic data
Retrieve the value(s) of attributes from an `ic.df` dataset. While
intended to be used with the core ic attributes, it will work with any named
attribute.

```
get_attr(X, attrs = ic_core(), unlist = TRUE)
```

## Arguments

|  |  |
| --- | --- |
| X | An object of class `ic.df`. |
| attrs | A vector of character names of attributes to be retrieved. Default are all core attributes. |
| unlist | Should the function return a named list of attributes and values or a named vector? Default is `TRUE` to return a vector. |

## Contents

Developed by Warren C. Jochem, Edson Utazi, Sahu Sujit.

Site built with pkgdown 1.6.1.
