# Supplementary material for: Bayesian Hierarchical Modeling Approaches for Combining Information From Multiple Data Sources to Produce Annual Estimates of National Immunization Coverage
Source: Biom J. 2026 Oct 6;68(5):e70189. doi: 10.1002/bimj.70189 (PMC13652340; doi:10.1002/bimj.70189)
Supplement: Supplementary file 2 [file BIMJ-68-e70189-s002.zip › code_and_data/imcover/imcover_0_2_5.zip!/imcover-main/docs/reference/get_region.html]

Get regional groupings for country codes
Retrieve a list of the region based on the ISO3c alpha codes. — get\_region • imcover


Toggle navigation


imcover
0.2.4

- Reference
- Articles
  - Model-based approaches to immunization coverage estimations in `R`: An introduction to the `imcover` package
- Changelog

# Get regional groupings for country codes Retrieve a list of the region based on the ISO3c alpha codes.

`get_region.Rd`

Get regional groupings for country codes
Retrieve a list of the region based on the ISO3c alpha codes.

```
get_region(country = NULL, type = "who")
```

## Arguments

|  |  |
| --- | --- |
| country | Vector of country ISO3 codes as characters. Default is `NULL` which returns all regions in the dataset. |
| type | The type of region grouping to return. The default `'who'` returns World Health Organisation codes. The other option is 'm49' for UN Stats Division region groupings. |

## Value

A character vector of region codes of length equal
`length(country)` if `country` is specified or a `data.frame` of
the region table if `country` is `NULL`.

## Details

Data source of M49 regions:
https://unstats.un.org/unsd/methodology/m49/overview/

## Contents

Developed by Warren C. Jochem, Edson Utazi, Sahu Sujit.

Site built with pkgdown 1.6.1.
