# Supplementary material for: Bayesian Hierarchical Modeling Approaches for Combining Information From Multiple Data Sources to Produce Annual Estimates of National Immunization Coverage
Source: Biom J. 2026 Oct 6;68(5):e70189. doi: 10.1002/bimj.70189 (PMC13652340; doi:10.1002/bimj.70189)
Supplement: Supplementary file 2 [file BIMJ-68-e70189-s002.zip › code_and_data/imcover/imcover_0_2_5.zip!/imcover-main/docs/reference/get_yovi.html]

Get year of introduction for vaccines
Retrieve a list of the year when a vaccine was introduced to the entire
country. — get\_yovi • imcover


Toggle navigation


imcover
0.2.4

- Reference
- Articles
  - Model-based approaches to immunization coverage estimations in `R`: An introduction to the `imcover` package
- Changelog

# Get year of introduction for vaccines Retrieve a list of the year when a vaccine was introduced to the entire country.

`get_yovi.Rd`

Get year of introduction for vaccines
Retrieve a list of the year when a vaccine was introduced to the entire
country.

```
get_yovi(vaccine = NULL)
```

## Arguments

|  |  |
| --- | --- |
| vaccine | Vector of vaccine codes as characters. Default is `NULL` which returns all vaccines in the dataset. |

## Details

Data source of vaccine schedules:
https://www.who.int/immunization/monitoring\_surveillance/data/year\_vaccine\_introduction.xlsx

## Contents

Developed by Warren C. Jochem, Edson Utazi, Sahu Sujit.

Site built with pkgdown 1.6.1.
