# Supplementary material for: Bayesian Hierarchical Modeling Approaches for Combining Information From Multiple Data Sources to Produce Annual Estimates of National Immunization Coverage
Source: Biom J. 2026 Oct 6;68(5):e70189. doi: 10.1002/bimj.70189 (PMC13652340; doi:10.1002/bimj.70189)
Supplement: Supplementary file 2 [file BIMJ-68-e70189-s002.zip › code_and_data/imcover/imcover_0_2_5.zip!/imcover-main/docs/reference/ic.df.html]

Extract or replace parts of ic data — ic.df • imcover


Toggle navigation


imcover
0.2.4

- Reference
- Articles
  - Model-based approaches to immunization coverage estimations in `R`: An introduction to the `imcover` package
- Changelog

# Extract or replace parts of ic data

`ic.df.Rd`

Operators to extract or replace subsets of ic data objects.

```
# S3 method for ic.df
[(x, i, j, ..., drop = FALSE)
```

## Arguments

|  |  |
| --- | --- |
| x | Object of class `ic.df` from which to extract element(s) or in which to replace element(s). |
| i, j, ... | Indices specifying the elements to extract or replace. Numeric or character vector or empty (missing) or NULL. |
| drop | Can core ic attribute columns be modified? Default is `FALSE`. |

## Value

For `[` an `ic.df` data frame. For `[[` or `$`, a
column of the ic data frame. For `[<-`, `[[<-`, and `$<-`, a
modified ic data frame.

## Details

The use of `[.ic.df` will generally follow the behaviour of
`data.frame`; however, when `drop = FALSE` the core 'ic'
attributes will always be preserved and will always return an object of
class `ic.df`.

## See also

`ic_data`

## Contents

Developed by Warren C. Jochem, Edson Utazi, Sahu Sujit.

Site built with pkgdown 1.6.1.
