# Supplementary material for: Bayesian Hierarchical Modeling Approaches for Combining Information From Multiple Data Sources to Produce Annual Estimates of National Immunization Coverage
Source: Biom J. 2026 Oct 6;68(5):e70189. doi: 10.1002/bimj.70189 (PMC13652340; doi:10.1002/bimj.70189)
Supplement: Supplementary file 2 [file BIMJ-68-e70189-s002.zip › code_and_data/imcover/imcover_0_2_5.zip!/imcover-main/docs/reference/ic_adjust.html]

Adjust 'ic' coverage data — ic\_adjust • imcover


Toggle navigation


imcover
0.2.4

- Reference
- Articles
  - Model-based approaches to immunization coverage estimations in `R`: An introduction to the `imcover` package
- Changelog

# Adjust 'ic' coverage data

`ic_adjust.Rd`

Make adjustments to vaccination coverage to ensure reported coverages
preserve the ratio of multi-dose vaccines and that coverage is >0% and <100%.

```
ic_adjust(
  X,
  ratio_adj = FALSE,
  numerator = "DTP3",
  denominator = "DTP1",
  coverage_adj = TRUE
)

ic_ratio(X, numerator = "DTP3", denominator = "DTP1")
```

## Arguments

|  |  |
| --- | --- |
| X | Object of class `ic.df` to adjust |
| ratio\_adj | Logical. Should multi-dose vaccine coverages be adjusted by their ratio when coverage > 100%? Default is `FALSE`. |
| numerator | Character vector of vaccine identifiers (with dose numbers) to calculate the ratio adjustment. Default is 'DTP3'. |
| denominator | Character vector of vaccine identifiers (with dose numbers) to calculate the ratio adjustment. Default is 'DTP1'. |
| coverage\_adj | Logical. Should coverage percentage be changed to be >0% and <100%? Default is `TRUE`. |

## Details

This function provides minimal adjustments to immunisation data to
ensure that the coverages are suitable for statistical modelling. Ratio
adjustments help to ensure that multi-dose vaccines report a coverage that
is less-than-or-equal-to the coverage of the first dose.

Coverage adjustment accounts for inaccurate target populations that result
is coverages of >100%. By maintaining a coverage between 0 and 100% a
logistic transformation can be used in the statistical model.

## See also

`ic_data`

## Contents

Developed by Warren C. Jochem, Edson Utazi, Sahu Sujit.

Site built with pkgdown 1.6.1.
