# Supplementary material for: Bayesian Hierarchical Modeling Approaches for Combining Information From Multiple Data Sources to Produce Annual Estimates of National Immunization Coverage
Source: Biom J. 2026 Oct 6;68(5):e70189. doi: 10.1002/bimj.70189 (PMC13652340; doi:10.1002/bimj.70189)
Supplement: Supplementary file 2 [file BIMJ-68-e70189-s002.zip › code_and_data/imcover/imcover_0_2_5.zip!/imcover-main/docs/reference/ic_core.html]

List core attributes for ic data
Retrieve the names of the core attributes that can be expected in
ic.df data. — ic\_core • imcover


Toggle navigation


imcover
0.2.4

- Reference
- Articles
  - Model-based approaches to immunization coverage estimations in `R`: An introduction to the `imcover` package
- Changelog

# List core attributes for ic data Retrieve the names of the core attributes that can be expected in `ic.df` data.

`ic_core.Rd`

List core attributes for ic data
Retrieve the names of the core attributes that can be expected in
`ic.df` data.

```
ic_core(survey = TRUE)
```

## Arguments

|  |  |
| --- | --- |
| survey | Include survey-specific attributes or only `ic.df` attributes? Default is `TRUE`. |

## Contents

Developed by Warren C. Jochem, Edson Utazi, Sahu Sujit.

Site built with pkgdown 1.6.1.
