# Supplementary material for: Bayesian Hierarchical Modeling Approaches for Combining Information From Multiple Data Sources to Produce Annual Estimates of National Immunization Coverage
Source: Biom J. 2026 Oct 6;68(5):e70189. doi: 10.1002/bimj.70189 (PMC13652340; doi:10.1002/bimj.70189)
Supplement: Supplementary file 2 [file BIMJ-68-e70189-s002.zip › code_and_data/imcover/imcover_0_2_5.zip!/imcover-main/docs/reference/ic_coverage.html]

Summarise posterior estimates of immunisation coverage — ic\_coverage • imcover


Toggle navigation


imcover
0.2.4

- Reference
- Articles
  - Model-based approaches to immunization coverage estimations in `R`: An introduction to the `imcover` package
- Changelog

# Summarise posterior estimates of immunisation coverage

`ic_coverage.Rd`

Computes the Bayesian posterior summary intervals, sometimes
called 'credible intervals' for the estimated immunisation coverage term
('mu').

```
ic_coverage(
  X,
  object = "posterior",
  stat = c("mean", "median", "sd", "quantile"),
  probs = c(0.025, 0.25, 0.5, 0.75, 0.975)
)

# S3 method for icfit
ic_coverage(
  X,
  object = "posterior",
  stat = c("mean", "median", "sd", "quantile"),
  probs = c(0.025, 0.25, 0.5, 0.75, 0.975)
)

# S3 method for iclist
ic_coverage(
  X,
  object = "posterior",
  stat = c("mean", "median", "sd", "quantile"),
  probs = c(0.025, 0.25, 0.5, 0.75, 0.975)
)
```

## Arguments

|  |  |
| --- | --- |
| X | A fitted model object of type `icfit` or `iclist`. |
| object | Character specifying the name of the object to summarise. Default is 'posterior'. |
| stat | Character vector of summary statistics to apply. |
| probs | Numeric vector of probabilities with values in 0,1 to be used for `stat` "quantile". |

## Value

A `data.frame` with columns for 'country', 'time', and 'vaccine'
labels and summary statistics, and as many rows as there are 'mu'
observations of immunisation coverage.

## Contents

Developed by Warren C. Jochem, Edson Utazi, Sahu Sujit.

Site built with pkgdown 1.6.1.
