# Supplementary material for: Bayesian Hierarchical Modeling Approaches for Combining Information From Multiple Data Sources to Produce Annual Estimates of National Immunization Coverage
Source: Biom J. 2026 Oct 6;68(5):e70189. doi: 10.1002/bimj.70189 (PMC13652340; doi:10.1002/bimj.70189)
Supplement: Supplementary file 2 [file BIMJ-68-e70189-s002.zip › code_and_data/imcover/imcover_0_2_5.zip!/imcover-main/docs/reference/ic_data.html]

Create an ic data object — ic\_data • imcover


Toggle navigation


imcover
0.2.4

- Reference
- Articles
  - Model-based approaches to immunization coverage estimations in `R`: An introduction to the `imcover` package
- Changelog

# Create an ic data object

`ic_data.Rd`

Create an ic data object, which extends `data.frame`-like objects by
defining consistent attributes for required data elements.

```
ic_data(
  X,
  region = "region",
  country = "code",
  time = "year",
  vaccine = "antigen",
  coverage = "coverage",
  source = "coverage_category",
  dose = "doses",
  population = "target_number",
  drop_cols = FALSE,
  ...
)

# S3 method for ic.df
ic_data(
  X,
  region = "region",
  country = "code",
  time = "year",
  vaccine = "antigen",
  coverage = "coverage",
  source = "coverage_category",
  dose = "doses",
  population = "target_number",
  drop_cols = FALSE,
  ...
)

# S3 method for data.frame
ic_data(
  X,
  region = "region",
  country = "code",
  time = "year",
  vaccine = "antigen",
  coverage = "coverage",
  source = "coverage_category",
  dose = "doses",
  population = "target_number",
  drop_cols = FALSE,
  ...
)
```

## Arguments

|  |  |
| --- | --- |
| X | An `R` object to process and convert to ic data. |
| region, country, time, vaccine, coverage, source, dose, population | Character of the column name within `X` which defines the core value for ic data. See details. |
| drop\_cols | Should other columns in `X` be dropped if they are not core attributes? Default is `FALSE` to retain all data from `X`. |
| ... | Additional arguments. Not currently used. |

## Value

An object of class `ic.df` which extends `data.frame` with
attributes to locate and preserve core data elements on immunisation
coverage.

## Details

`ic_data` is the core function to create a properly formed and
processed dataset for immunisation coverage modelling using `imcover`.
In particular it requires some core data elements are present:

- 'region' Larger grouping, such as WHO-defined groups of countries.
  Used to apply separate models.
- 'country' Primary grouping for data records. Default name is
  'code' referring to an ISO3 code of a country.
- 'time' Defines the time period of immunisation records, typically
  an integer year. Default column name is 'year'.
- 'vaccine' Code to identify the vaccine records, e.g. 'DTP1'.
  Default column name is 'antigen'.
- 'coverage' Pre-calculated coverage percentage for each country,
  time, vaccine observation. Default column name is 'coverage'.
- 'source' Character identifying the source of the data. Default
  column name is 'coverage\_category'.

If 'coverage' is not included in `X`, then two other elements are
required to be specified in order for percent coverage to be calculated.
Else, these are optional elements for ic data.

- 'dose' Number of vaccine doses administered. Default column name is
  'doses'.
- 'population' Total target population for the vaccine. Default
  column name is 'target\_number.

This function is for use with administrative records. For survey datasets,
please use `ic_survey`.

## See also

`ic_survey`, `ic_expand`,
`ic_validate`

## Examples

```
if (FALSE) {
# assume `df` is a data.frame
# convert to an imcover data frame
ic_data(df,
       group = "iso3",  # specify the column names found in `df`
       time = "cohortyear",
       vaccine = "vaccine",
       coverage = "coverage")
}
```

## Contents

Developed by Warren C. Jochem, Edson Utazi, Sahu Sujit.

Site built with pkgdown 1.6.1.
