# Supplementary material for: Bayesian Hierarchical Modeling Approaches for Combining Information From Multiple Data Sources to Produce Annual Estimates of National Immunization Coverage
Source: Biom J. 2026 Oct 6;68(5):e70189. doi: 10.1002/bimj.70189 (PMC13652340; doi:10.1002/bimj.70189)
Supplement: Supplementary file 2 [file BIMJ-68-e70189-s002.zip › code_and_data/imcover/imcover_0_2_5.zip!/imcover-main/docs/reference/ic_expand.html]

Expand ic data to include all potential years. — ic\_expand • imcover


Toggle navigation


imcover
0.2.4

- Reference
- Articles
  - Model-based approaches to immunization coverage estimations in `R`: An introduction to the `imcover` package
- Changelog

# Expand ic data to include all potential years.

`ic_expand.Rd`

Expand the time dimension of an ic dataset to include the full set of
potential observations.

```
ic_expand(X, min = 1999, max, na.remove = TRUE)
```

## Arguments

|  |  |
| --- | --- |
| X | Object of class `ic.df` to expand. |
| min | Start of the potential time period. Default is 1999. |
| max | End of the potential time period. |
| na.remove | Should country-vaccine combinations which have no coverage data be removed? Default is `TRUE` |

## Value

An object of type `ic.df` which has additional rows of `NA`
values added to expand the dataset to min and max time points.

## Details

If `max` is missing, the maximum time value observed in the
dataset will be used.

## Contents

Developed by Warren C. Jochem, Edson Utazi, Sahu Sujit.

Site built with pkgdown 1.6.1.
