# Supplementary material for: Bayesian Hierarchical Modeling Approaches for Combining Information From Multiple Data Sources to Produce Annual Estimates of National Immunization Coverage
Source: Biom J. 2026 Oct 6;68(5):e70189. doi: 10.1002/bimj.70189 (PMC13652340; doi:10.1002/bimj.70189)
Supplement: Supplementary file 2 [file BIMJ-68-e70189-s002.zip › code_and_data/imcover/imcover_0_2_5.zip!/imcover-main/docs/reference/ic_filter.html]

Filter immunisation coverage records — ic\_filter • imcover


Toggle navigation


imcover
0.2.4

- Reference
- Articles
  - Model-based approaches to immunization coverage estimations in `R`: An introduction to the `imcover` package
- Changelog

# Filter immunisation coverage records

`ic_filter.Rd`

Select records from 'ic' objects based on region group, country, time, or
vaccine.

```
ic_filter(X, region, country, time, vaccine)

# S3 method for ic.df
ic_filter(X, region, country, time, vaccine)

# S3 method for icfit
ic_filter(X, country, time, vaccine)

# S3 method for iclist
ic_filter(X, region, country, time, vaccine)
```

## Arguments

|  |  |
| --- | --- |
| X | object of type `ic.df`, `icfit`, or `iclist` |
| region | Character vector of regional groups to select. Not valid for `icfit`. |
| country | Character vector of country identifiers to select |
| time | Vector of time points to select |
| vaccine | Character vector of vaccine records to select, given by abbreviations (e.g. 'DTP1') |

## Value

A new object with type matching `X` with the selected records.

## Details

For `icfit` and `iclist` objects, filtering will only
affect the posterior estimates and prediction (if present). When a
selection parameter is omitted, then all possible values will be selected.
Selection parameters which are not present in `X` will be ignored.

## Contents

Developed by Warren C. Jochem, Edson Utazi, Sahu Sujit.

Site built with pkgdown 1.6.1.
