# Supplementary material for: Bayesian Hierarchical Modeling Approaches for Combining Information From Multiple Data Sources to Produce Annual Estimates of National Immunization Coverage
Source: Biom J. 2026 Oct 6;68(5):e70189. doi: 10.1002/bimj.70189 (PMC13652340; doi:10.1002/bimj.70189)
Supplement: Supplementary file 2 [file BIMJ-68-e70189-s002.zip › code_and_data/imcover/imcover_0_2_5.zip!/imcover-main/docs/reference/ic_fit.html]

Multi-source immunisation coverage model with Stan — ic\_fit • imcover


Toggle navigation


imcover
0.2.4

- Reference
- Articles
  - Model-based approaches to immunization coverage estimations in `R`: An introduction to the `imcover` package
- Changelog

# Multi-source immunisation coverage model with Stan

`ic_fit.Rd`

Multi-source immunisation coverage model with Stan

```
ic_fit(
  X,
  prior_lambda = NULL,
  prior_sigma = NULL,
  upper_sigma = NULL,
  lower_sigma = NULL,
  region = TRUE,
  verbose = TRUE,
  ...
)

# S3 method for ic.df
ic_fit(
  X,
  prior_lambda = NULL,
  prior_sigma = NULL,
  upper_sigma = NULL,
  lower_sigma = NULL,
  region = TRUE,
  verbose = TRUE,
  ...
)

# S3 method for ic.df
ic_fit_single(X, region = TRUE, verbose = TRUE, ...)
```

## Arguments

|  |  |
| --- | --- |
| X | Object of `ic.df` for analysis |
| prior\_lambda | Scale parameter for normal prior on source-specific intercepts. See details. |
| prior\_sigma | Scale parameter for the truncated Cauchy prior on source-specific standard deviations. See details. |
| upper\_sigma | Numeric value (or vector) setting the upper bounds on the source-specific scale parameter. See details. |
| lower\_sigma | Numeric value (or vector) setting the lower bounds on the source-specific scale parameter. See details. |
| region | Logical. Should region-specific models be generated? Default is `TRUE`. |
| verbose | Logical. Should messages be displayed? Default is `TRUE`. |
| ... | Arguments passed to `rstan::sampling` (e.g. iter, chains). |

## Value

An object of class `icfit` for single region models, or
`iclist` for multiple regions. The `icfit` object contains a
`stanfit` object returned by `rstan::sampling` with the fitted
object along with the posterior samples, data used in the model, and other
attributes related to the fitting. An `iclist` object is an extension
of a list containing multiple `icfit` objects.

## Details

The default priors for the source-specific intercepts (lambda) and
standard deviations (sigma) are given by normal and truncated Cauchy
distributions, respectively. Future version may allow users to specify
different distributions. Currently, users may only specify the scale
parameter of these distribution. By default, lambda for all sources is
given a value of 0.5, i.e. a prior distribution of `normal(0, 0.5)`.

For sigma, the default prior varies by source. While administrative and
official data are set to a value of 2, survey data is restricted to 0.2,
reflecting a prior belief in more accurate measurements. These parameters
are used in a half-Cauchy distribution (e.g. `cauchy(0, 0.2)`).

In addition, the upper and lower bounds of the sigma parameter may be set
to define the range of the possible values. In general, the lower bounds
should always be zero and cannot be negative. Similar to the differences in
the prior distributions, an upper bound of 0.4 is placed on 'survey'
estimates. In the absence of a user-defined upper-bound, these are set to
100.

Users setting `prior_lambda`, `prior_sigma`, or
`upper_sigma` should note that the length of values specified must be
1 or match the number of unique sources in `X`. When only one value is
present, it will be applied to all sources. When a vector is provided, the
order of values must match the order of sources as given by
`list_sources(X)`.

## Contents

Developed by Warren C. Jochem, Edson Utazi, Sahu Sujit.

Site built with pkgdown 1.6.1.
