# Supplementary material for: Bayesian Hierarchical Modeling Approaches for Combining Information From Multiple Data Sources to Produce Annual Estimates of National Immunization Coverage
Source: Biom J. 2026 Oct 6;68(5):e70189. doi: 10.1002/bimj.70189 (PMC13652340; doi:10.1002/bimj.70189)
Supplement: Supplementary file 2 [file BIMJ-68-e70189-s002.zip › code_and_data/imcover/imcover_0_2_5.zip!/imcover-main/docs/reference/ic_fit_single.html]

Multi-source immunisation coverage model with Stan — ic\_fit\_single • imcover


Toggle navigation


imcover
0.2.4

- Reference
- Articles
  - Model-based approaches to immunization coverage estimations in `R`: An introduction to the `imcover` package
- Changelog

# Multi-source immunisation coverage model with Stan

`ic_fit_single.Rd`

Sampling from a single likelihood model using a random effect
for data source.

```
ic_fit_single(X, region = TRUE, verbose = TRUE, ...)
```

## Arguments

|  |  |
| --- | --- |
| X | Object of `ic.df` for analysis |
| region | Logical. Should region-specific models be generated? Default is `TRUE`. |
| verbose | Logical. Should messages be displayed? Default is `TRUE`. |
| ... | Arguments passed to `rstan::sampling` (e.g. iter, chains). |

## Value

An object of class `icfit` for single region models, or
`iclist` for multiple regions. The `icfit` object contains a
`stanfit` object returned by `rstan::sampling` with the fitted
object along with the posterior samples, data used in the model, and other
attributes related to the fitting. An `iclist` object is an extension
of a list containing multiple `icfit` objects.

## Details

The current version of `imcover` does not allow for users to
modify the prior distributions in the single likelihood model. Future
versions of the package may enable this functionality. Lambda is assigned a
prior distribution of `normal(0, 1)` and the sigma parameters are
assigned half-Cauchy priors with a scale of 2.

## Contents

Developed by Warren C. Jochem, Edson Utazi, Sahu Sujit.

Site built with pkgdown 1.6.1.
