# Supplementary material for: Bayesian Hierarchical Modeling Approaches for Combining Information From Multiple Data Sources to Produce Annual Estimates of National Immunization Coverage
Source: Biom J. 2026 Oct 6;68(5):e70189. doi: 10.1002/bimj.70189 (PMC13652340; doi:10.1002/bimj.70189)
Supplement: Supplementary file 2 [file BIMJ-68-e70189-s002.zip › code_and_data/imcover/imcover_0_2_5.zip!/imcover-main/docs/reference/ic_plot.html]

Plot ic fit objects — ic\_plot • imcover


Toggle navigation


imcover
0.2.4

- Reference
- Articles
  - Model-based approaches to immunization coverage estimations in `R`: An introduction to the `imcover` package
- Changelog

# Plot ic fit objects

`ic_plot.Rd`

Provides methods to plot estimated time trends of vaccine coverage for
countries.

```
ic_plot(
  X,
  probs = c(0.025, 0.975),
  vaccine,
  observed = TRUE,
  prediction = TRUE,
  wuenic = FALSE,
  filter_yovi = FALSE,
  ncol = 4,
  interactive = FALSE
)

# S3 method for icfit
ic_plot(
  X,
  probs = c(0.025, 0.975),
  vaccine,
  observed = TRUE,
  prediction = TRUE,
  wuenic = FALSE,
  filter_yovi = FALSE,
  ncol = 4,
  interactive = FALSE
)

# S3 method for iclist
ic_plot(
  X,
  probs = c(0.025, 0.975),
  vaccine,
  observed = TRUE,
  prediction = TRUE,
  filter_yovi = FALSE,
  ncol = 4,
  interactive = FALSE
)
```

## Arguments

|  |  |
| --- | --- |
| X | Object of type `icfit` or `iclist` |
| probs | Numeric vector of length 2 for probabilities with values in 0,1 to be used to calculate the upper and lower credible intervals. |
| vaccine | Optional. Character vector to subset which vaccines to plot |
| observed | Logical. Should the observed data used to fit `X` be overlaid on the plot? Default is `TRUE`. |
| prediction | Logical. If predictions are found in the `icfit` object, should they be overlaid on the plot? Default is `TRUE`. |
| wuenic | Logical. Should WUENIC vaccination coverage estimates be overlaid on the plot? Default is `FALSE`. |
| filter\_yovi | Logical. Should the estimates be filtered by year of vaccine introduction? Default is `FALSE`. |
| ncol | When plotting multiple countries, the number of plots per page. |
| interactive | Logical. Should the output be interactive? Default is `FALSE`. |

## Value

A plot of the coverage estimates extracted from the fit, including
credible intervals.

## Details

If observed data are provided, they will be overlaid on the
estimates.

## Contents

Developed by Warren C. Jochem, Edson Utazi, Sahu Sujit.

Site built with pkgdown 1.6.1.
