# Supplementary material for: Bayesian Hierarchical Modeling Approaches for Combining Information From Multiple Data Sources to Produce Annual Estimates of National Immunization Coverage
Source: Biom J. 2026 Oct 6;68(5):e70189. doi: 10.1002/bimj.70189 (PMC13652340; doi:10.1002/bimj.70189)
Supplement: Supplementary file 2 [file BIMJ-68-e70189-s002.zip › code_and_data/imcover/imcover_0_2_5.zip!/imcover-main/docs/reference/ic_regional.html]

Regional immunisation coverage — ic\_regional • imcover


Toggle navigation


imcover
0.2.4

- Reference
- Articles
  - Model-based approaches to immunization coverage estimations in `R`: An introduction to the `imcover` package
- Changelog

# Regional immunisation coverage

`ic_regional.Rd`

Calculates population-weighted regional estimates from `icfit` objects.

```
ic_regional(
  X,
  denom,
  stat = c("mean", "median", "sd", "quantile"),
  probs = c(0.025, 0.25, 0.5, 0.75, 0.975),
  filter_yovi = TRUE
)

# S3 method for icfit
ic_regional(
  X,
  denom,
  stat = c("mean", "median", "sd", "quantile"),
  probs = c(0.025, 0.25, 0.5, 0.75, 0.975),
  filter_yovi = TRUE
)

# S3 method for iclist
ic_regional(
  X,
  denom,
  stat = c("mean", "median", "sd", "quantile"),
  probs = c(0.025, 0.25, 0.5, 0.75, 0.975),
  filter_yovi = TRUE
)
```

## Arguments

|  |  |
| --- | --- |
| X | Object of `icfit` or `iclist` from model fitting |
| denom | Population denominator data. Default is downloaded from WHO. |
| stat | Character vector of summary statistics to apply. |
| probs | Numeric vector of probabilities with values in 0,1 to be used for `stat` "quantile". |
| filter\_yovi | Logical. Should the estimates be filtered by year of vaccine introduction? |

## Value

`data.frame` object with regional summaries

## Contents

Developed by Warren C. Jochem, Edson Utazi, Sahu Sujit.

Site built with pkgdown 1.6.1.
