# Supplementary material for: Bayesian Hierarchical Modeling Approaches for Combining Information From Multiple Data Sources to Produce Annual Estimates of National Immunization Coverage
Source: Biom J. 2026 Oct 6;68(5):e70189. doi: 10.1002/bimj.70189 (PMC13652340; doi:10.1002/bimj.70189)
Supplement: Supplementary file 2 [file BIMJ-68-e70189-s002.zip › code_and_data/imcover/imcover_0_2_5.zip!/imcover-main/docs/reference/ic_survey.html]

Create an ic data object — ic\_survey • imcover


Toggle navigation


imcover
0.2.4

- Reference
- Articles
  - Model-based approaches to immunization coverage estimations in `R`: An introduction to the `imcover` package
- Changelog

# Create an ic data object

`ic_survey.Rd`

Create an ic data object from household survey data on
immunisation coverage.

```
ic_survey(
  X,
  ...,
  drop_cols = FALSE,
  survey = "surveyNameEnglish",
  sample = "Sample_Size",
  evidence = "evidence",
  validity = "validity",
  reduce = TRUE,
  minSample = 300
)

# S3 method for ic.df
ic_survey(
  X,
  ...,
  drop_cols = FALSE,
  survey = "surveyNameEnglish",
  sample = "Sample_Size",
  evidence = "evidence",
  validity = "validity",
  reduce = TRUE,
  minSample = 300
)

# S3 method for ic.df
ic_survey(
  X,
  ...,
  drop_cols = FALSE,
  survey = "surveyNameEnglish",
  sample = "Sample_Size",
  evidence = "evidence",
  validity = "validity",
  reduce = TRUE,
  minSample = 300
)

# S3 method for data.frame
ic_survey(
  X,
  ...,
  drop_cols = FALSE,
  survey = "surveyNameEnglish",
  sample = "Sample_Size",
  evidence = "evidence",
  validity = "validity",
  reduce = TRUE,
  minSample = 300
)
```

## Arguments

|  |  |
| --- | --- |
| X | Any object with immunisation coverage survey data to convert into an ic object. |
| ... | Additional parameters for names of core data elements to pass on to `ic_data`. See details and `ic_data`. |
| drop\_cols | Should other columns in `X` be dropped if they are not core attributes? Default is `FALSE` to retain all data from `X`. |
| survey, sample, evidence, validity | Character of the column name within `X` which defines the core values for ic survey data. |
| reduce | Should country-vaccine combinations which have insufficient coverage data be removed? Default is `TRUE`. |
| minSample | Numeric. Minimum reported sample size needed to keep coverage records. Default is 300. Only used with `reduce` is `TRUE`. |

## Value

An object of class `ic.df` which extends `data.frame` with
attributes to location and preserve core data elements for immunisation
coverage.

## Details

`ic_survey` is is an extension to `ic_data` and is used to
create a properly formed and processed dataset for immunisation coverage
modelling based on household survey datasets.

## See also

`ic_data`

## Examples

```
if (FALSE) {
# assume `df` is a data.frame
# convert to an imcover data frame
ic_survey(df,
         group="iso3",  # specify columns names in df
         time="cohortyear",
         vaccine="vaccine",
         coverage = "coverage",
         survey = "surveynameenglish",
         sample = "sample_size",
         evidence = "evidence",
         validity = "validity",
         biasAdjust = TRUE)
}
```

## Contents

Developed by Warren C. Jochem, Edson Utazi, Sahu Sujit.

Site built with pkgdown 1.6.1.
