# Supplementary material for: Bayesian Hierarchical Modeling Approaches for Combining Information From Multiple Data Sources to Produce Annual Estimates of National Immunization Coverage
Source: Biom J. 2026 Oct 6;68(5):e70189. doi: 10.1002/bimj.70189 (PMC13652340; doi:10.1002/bimj.70189)
Supplement: Supplementary file 2 [file BIMJ-68-e70189-s002.zip › code_and_data/imcover/imcover_0_2_5.zip!/imcover-main/docs/reference/ic_update.html]

Update ic data to adjust denominators. — ic\_update • imcover


Toggle navigation


imcover
0.2.4

- Reference
- Articles
  - Model-based approaches to immunization coverage estimations in `R`: An introduction to the `imcover` package
- Changelog

# Update ic data to adjust denominators.

`ic_update.Rd`

Provide data checking and validation by comparing across official estimates,
administrative records, and survey estimates of immunisation coverage.

```
ic_update(admin, denom, official, survey, dtpCheck = TRUE, limit = 10)
```

## Arguments

|  |  |
| --- | --- |
| admin | Object of class `ic.df` to containing administrative estimates of coverage. |
| denom | Object of class `ic.df` containing official WUENIC denominators. |
| official | Object of class `ic.df` containing official country estimates of coverage. |
| survey | Object of class `ic.svy` containing annual survey estimates of coverage. |
| dtpCheck | Should dose 3 and dose 1 of DTP vaccines be compared for each country and time? If dose 3 coverage exceeds dose 1, these records will marked `NA`. Default is `TRUE`. |
| limit | Maximum acceptable difference between survey and administrative coverage records. When the difference exceeds the limit, the survey estimate is preferred. Default is 10 percent. |

## See also

`ic_data`

## Contents

Developed by Warren C. Jochem, Edson Utazi, Sahu Sujit.

Site built with pkgdown 1.6.1.
