# Supplementary material for: Bayesian Hierarchical Modeling Approaches for Combining Information From Multiple Data Sources to Produce Annual Estimates of National Immunization Coverage
Source: Biom J. 2026 Oct 6;68(5):e70189. doi: 10.1002/bimj.70189 (PMC13652340; doi:10.1002/bimj.70189)
Supplement: Supplementary file 2 [file BIMJ-68-e70189-s002.zip › code_and_data/imcover/imcover_0_2_5.zip!/imcover-main/docs/reference/imcover-package.html]

imcover: An R package for immunisation coverage modelling — imcover-package • imcover


Toggle navigation


imcover
0.2.4

- Reference
- Articles
  - Model-based approaches to immunization coverage estimations in `R`: An introduction to the `imcover` package
- Changelog

# imcover: An R package for immunisation coverage modelling

`imcover-package.Rd`

Provides access to WUENIC immunisation data and a Bayesian statistical
modelling framework to produce country- and region-specific temporal trends
in immunisation coverage.

## Credits

This work was undertaken by members of the WorldPop Research Group at the
University of Southampton(https://www.worldpop.org/).

## Contents

Developed by Warren C. Jochem, Edson Utazi, Sahu Sujit.

Site built with pkgdown 1.6.1.
