# Supplementary material for: Bayesian Hierarchical Modeling Approaches for Combining Information From Multiple Data Sources to Produce Annual Estimates of National Immunization Coverage
Source: Biom J. 2026 Oct 6;68(5):e70189. doi: 10.1002/bimj.70189 (PMC13652340; doi:10.1002/bimj.70189)
Supplement: Supplementary file 2 [file BIMJ-68-e70189-s002.zip › code_and_data/imcover/imcover_0_2_5.zip!/imcover-main/docs/reference/index.html]

Function reference • imcover


Toggle navigation


imcover
0.2.4

- Reference
- Articles
  - Model-based approaches to immunization coverage estimations in `R`: An introduction to the `imcover` package
- Changelog

# Reference

| All functions | |
| --- | --- |
|  |  |
| --- | --- |
| `as.ic_data()` `is.ic_data()` | Convert foreign object to an ic data object |
| `download_wuenic()` `download_coverage()` `download_survey()` `download_denom()` | Download WUENIC data files |
| `filter_yovi()` | Filter ic data records based on year of vaccine introduction Exclude ic data records for years when a vaccine had not been fully introduced in a country. |
| `get_attr()` | Get the attributes of ic data Retrieve the value(s) of attributes from an `ic.df` dataset. While intended to be used with the core ic attributes, it will work with any named attribute. |
| `get_region()` | Get regional groupings for country codes Retrieve a list of the region based on the ISO3c alpha codes. |
| `get_yovi()` | Get year of introduction for vaccines Retrieve a list of the year when a vaccine was introduced to the entire country. |
| `` `[`(<ic.df>) `` | Extract or replace parts of ic data |
| `ic_adjust()` `ic_ratio()` | Adjust 'ic' coverage data |
| `ic_core()` | List core attributes for ic data Retrieve the names of the core attributes that can be expected in `ic.df` data. |
| `ic_coverage()` | Summarise posterior estimates of immunisation coverage |
| `ic_data()` | Create an ic data object |
| `ic_expand()` | Expand ic data to include all potential years. |
| `ic_filter()` | Filter immunisation coverage records |
| `ic_fit()` `ic_fit_single(<ic.df>)` | Multi-source immunisation coverage model with Stan |
| `ic_fit_single()` | Multi-source immunisation coverage model with Stan |
| `ic_plot()` | Plot ic fit objects |
| `ic_regional()` | Regional immunisation coverage |
| `ic_survey()` | Create an ic data object |
| `ic_update()` | Update ic data to adjust denominators. |
| `imcover-package` | imcover: An R package for immunisation coverage modelling |
| `list_vaccines()` `list_countries()` `list_times()` `list_sources()` | List unique attributes |
| `log_lik(<icfit>)` | Extract posterior sample of the pointwise log-likelihood from a icfit object. |
| `loo(<icfit>)` `loo(<iclist>)` | Leave-one-out cross-validation |
| `merge(<ic.df>)` | Merge two ic data objects Merge two `ic.df` data objects by common columns while preserving the attributes |
| `` `names<-`(<ic.df>) `` `names(<ic.df>)` | The names of an ic object |
| `plot(<icfit>)` | Plot ic fit objects |
| `plot(<iclist>)` | Plot ic fit objects |
| `predict(<icfit>)` | Posterior predictions of vaccination coverage |
| `rbind(<ic.df>)` | Combine ic data objects by rows |
| `summary(<icfit>)` `summary(<iclist>)` `print(<summary.icfit>)` `print(<summary.iclist>)` | Summary methods for 'ic' fit objects |
| `survey_adjust()` | Survey recall-bias adjustment |
| `survey_reduce()` | Select survey records from an `ic_survey` dataset |
| `waic(<icfit>)` `waic(<iclist>)` | Widely applicable information criterion (WAIC) |

## Contents

Developed by Warren C. Jochem, Edson Utazi, Sahu Sujit.

Site built with pkgdown 1.6.1.
