# Supplementary material for: Bayesian Hierarchical Modeling Approaches for Combining Information From Multiple Data Sources to Produce Annual Estimates of National Immunization Coverage
Source: Biom J. 2026 Oct 6;68(5):e70189. doi: 10.1002/bimj.70189 (PMC13652340; doi:10.1002/bimj.70189)
Supplement: Supplementary file 2 [file BIMJ-68-e70189-s002.zip › code_and_data/imcover/imcover_0_2_5.zip!/imcover-main/docs/reference/list_vaccines.html]

List unique attributes — list\_vaccines • imcover


Toggle navigation


imcover
0.2.4

- Reference
- Articles
  - Model-based approaches to immunization coverage estimations in `R`: An introduction to the `imcover` package
- Changelog

# List unique attributes

`list_vaccines.Rd`

Retrieve the unique list of attributes observed in an ic dataset.

```
list_vaccines(X)

# S3 method for ic.df
list_vaccines(X)

# S3 method for icfit
list_vaccines(X)

list_countries(X)

# S3 method for ic.df
list_countries(X)

# S3 method for icfit
list_countries(X)

list_times(X)

# S3 method for ic.df
list_times(X)

# S3 method for icfit
list_times(X)

list_sources(X)

# S3 method for ic.df
list_sources(X)
```

## Arguments

|  |  |
| --- | --- |
| X | Object of class `ic.df`. |

## Contents

Developed by Warren C. Jochem, Edson Utazi, Sahu Sujit.

Site built with pkgdown 1.6.1.
