# Supplementary material for: Bayesian Hierarchical Modeling Approaches for Combining Information From Multiple Data Sources to Produce Annual Estimates of National Immunization Coverage
Source: Biom J. 2026 Oct 6;68(5):e70189. doi: 10.1002/bimj.70189 (PMC13652340; doi:10.1002/bimj.70189)
Supplement: Supplementary file 2 [file BIMJ-68-e70189-s002.zip › code_and_data/imcover/imcover_0_2_5.zip!/imcover-main/docs/reference/log_lik.icfit.html]

Extract posterior sample of the pointwise log-likelihood from a icfit object. — log\_lik.icfit • imcover


Toggle navigation


imcover
0.2.4

- Reference
- Articles
  - Model-based approaches to immunization coverage estimations in `R`: An introduction to the `imcover` package
- Changelog

# Extract posterior sample of the pointwise log-likelihood from a icfit object.

`log_lik.icfit.Rd`

Convenience function to extract a log-likelihood matrix or array from the
fitted Stan model in an `icfit` object.

```
# S3 method for icfit
log_lik(X, permuted = TRUE, ...)
```

## Arguments

|  |  |
| --- | --- |
| X | object of type `icfit` |
| permuted | Logical. Should the draws from each chain be permuted and merged?. Default is `TRUE` to merge. |
| ... | Not currently used. |

## Value

An S x N matrix containing pointwise log-likelihood samples, where S
is the number of samples and N is the number of observations in the data.
If `permuted` is `FALSE` then an S x N x R array is returned,
where R is the number of chains.

## See also

`extract_log_lik`

## Contents

Developed by Warren C. Jochem, Edson Utazi, Sahu Sujit.

Site built with pkgdown 1.6.1.
