# Supplementary material for: Bayesian Hierarchical Modeling Approaches for Combining Information From Multiple Data Sources to Produce Annual Estimates of National Immunization Coverage
Source: Biom J. 2026 Oct 6;68(5):e70189. doi: 10.1002/bimj.70189 (PMC13652340; doi:10.1002/bimj.70189)
Supplement: Supplementary file 2 [file BIMJ-68-e70189-s002.zip › code_and_data/imcover/imcover_0_2_5.zip!/imcover-main/docs/reference/loo.icfit.html]

Leave-one-out cross-validation — loo.icfit • imcover


Toggle navigation


imcover
0.2.4

- Reference
- Articles
  - Model-based approaches to immunization coverage estimations in `R`: An introduction to the `imcover` package
- Changelog

# Leave-one-out cross-validation

`loo.icfit.Rd`

The `loo` method for `icfit` objects. computes the approximate
leave-one-out cross-validation using Pareto smoothed importance sampling
(PSIS-LOO CV).

```
# S3 method for icfit
loo(X, ...)

# S3 method for iclist
loo(X, ...)
```

## Arguments

|  |  |
| --- | --- |
| X | Object of type `icfit` or `iclist` |
| ... | Additional parameters used by `rstan::loo`. |

## Value

an object of class `loo` with the PSIS results.

## See also

`loo`, `loo`

## Contents

Developed by Warren C. Jochem, Edson Utazi, Sahu Sujit.

Site built with pkgdown 1.6.1.
