# Supplementary material for: Bayesian Hierarchical Modeling Approaches for Combining Information From Multiple Data Sources to Produce Annual Estimates of National Immunization Coverage
Source: Biom J. 2026 Oct 6;68(5):e70189. doi: 10.1002/bimj.70189 (PMC13652340; doi:10.1002/bimj.70189)
Supplement: Supplementary file 2 [file BIMJ-68-e70189-s002.zip › code_and_data/imcover/imcover_0_2_5.zip!/imcover-main/docs/reference/merge.ic.df.html]

Merge two ic data objects
Merge two ic.df data objects by common columns while preserving the
attributes — merge.ic.df • imcover


Toggle navigation


imcover
0.2.4

- Reference
- Articles
  - Model-based approaches to immunization coverage estimations in `R`: An introduction to the `imcover` package
- Changelog

# Merge two ic data objects Merge two `ic.df` data objects by common columns while preserving the attributes

`merge.ic.df.Rd`

Merge two ic data objects
Merge two `ic.df` data objects by common columns while preserving the
attributes

```
# S3 method for ic.df
merge(x, y, attr.x = TRUE, ...)
```

## Arguments

|  |  |
| --- | --- |
| x, y | `ic.df` objects to merge |
| attr.x | Logical. Should the attributes (i.e. core field names) be based on the ic object 'x' or 'y' (when `FALSE`)? |
| ... | arguments to be based on `merge`. |

## Contents

Developed by Warren C. Jochem, Edson Utazi, Sahu Sujit.

Site built with pkgdown 1.6.1.
