# Supplementary material for: Bayesian Hierarchical Modeling Approaches for Combining Information From Multiple Data Sources to Produce Annual Estimates of National Immunization Coverage
Source: Biom J. 2026 Oct 6;68(5):e70189. doi: 10.1002/bimj.70189 (PMC13652340; doi:10.1002/bimj.70189)
Supplement: Supplementary file 2 [file BIMJ-68-e70189-s002.zip › code_and_data/imcover/imcover_0_2_5.zip!/imcover-main/docs/reference/multi_lik_stan.html]

Multi-source immunisation coverage model with Stan — multi\_lik\_stan • imcover


Toggle navigation


imcover
0.2.4

- Reference
- Articles
  - Model-based approaches to immunization coverage estimations in `R`: An introduction to the `imcover` package
- Changelog

# Multi-source immunisation coverage model with Stan

`multi_lik_stan.Rd`

Multi-source immunisation coverage model with Stan

```
multi_lik_stan(
  X,
  prior_lambda = NULL,
  prior_sigma = NULL,
  upper_sigma = NULL,
  lower_sigma = NULL,
  verbose = TRUE,
  ...
)
```

## Arguments

|  |  |
| --- | --- |
| X | Object of `ic.df` for analysis |
| prior\_lambda | Scale parameter for half-normal prior on source-specific intercepts. See details. |
| prior\_sigma | Scale parameter for half-Cauchy prior on source-specific standard deviations. See details. |
| upper\_sigma | Numeric value (or vector) setting the upper bounds on the source-specific scale parameter. See details. |
| lower\_sigma | Numeric value (or vector) setting the lower bounds on the source-specific scale parameter. See details. |
| verbose | Logical. Should messages be displayed? Default is `TRUE`. |
| ... | Arguments passed to `rstan::sampling` (e.g. iter, chains). |

## Value

An object of class `stanfit` returned by `rstan::sampling`.

## Contents

Developed by Warren C. Jochem, Edson Utazi, Sahu Sujit.

Site built with pkgdown 1.6.1.
