# Supplementary material for: Bayesian Hierarchical Modeling Approaches for Combining Information From Multiple Data Sources to Produce Annual Estimates of National Immunization Coverage
Source: Biom J. 2026 Oct 6;68(5):e70189. doi: 10.1002/bimj.70189 (PMC13652340; doi:10.1002/bimj.70189)
Supplement: Supplementary file 2 [file BIMJ-68-e70189-s002.zip › code_and_data/imcover/imcover_0_2_5.zip!/imcover-main/docs/reference/names.html]

The names of an ic object — names • imcover


Toggle navigation


imcover
0.2.4

- Reference
- Articles
  - Model-based approaches to immunization coverage estimations in `R`: An introduction to the `imcover` package
- Changelog

# The names of an ic object

`names.Rd`

Functions to get or set the names of an `ic.df`.

```
# S3 method for ic.df
names(x) <- value

# S3 method for ic.df
names(x)
```

## Arguments

|  |  |
| --- | --- |
| x | An `ic.df` object. |
| value | A character vector of up to the same length as `x` or NULL. |

## Value

For `names` a character vector the same length as `x`. For
`names<-`, the updated ic data frame object is returned.

## Details

These access and replacement functions which follow the same
functionality as `base::names` for data frames.

## See also

`ic_data`

## Contents

Developed by Warren C. Jochem, Edson Utazi, Sahu Sujit.

Site built with pkgdown 1.6.1.
