# Supplementary material for: Bayesian Hierarchical Modeling Approaches for Combining Information From Multiple Data Sources to Produce Annual Estimates of National Immunization Coverage
Source: Biom J. 2026 Oct 6;68(5):e70189. doi: 10.1002/bimj.70189 (PMC13652340; doi:10.1002/bimj.70189)
Supplement: Supplementary file 2 [file BIMJ-68-e70189-s002.zip › code_and_data/imcover/imcover_0_2_5.zip!/imcover-main/docs/reference/plot.icfit.html]

Plot ic fit objects — plot.icfit • imcover


Toggle navigation


imcover
0.2.4

- Reference
- Articles
  - Model-based approaches to immunization coverage estimations in `R`: An introduction to the `imcover` package
- Changelog

# Plot ic fit objects

`plot.icfit.Rd`

Provides methods to plot estimated time trends of vaccine coverage for
countries.

```
# S3 method for icfit
plot(X, ...)
```

## Arguments

|  |  |
| --- | --- |
| X | Object of type `icfit` or `iclist` |
| ... | Additional arguments passed to `ic_plot`. |

## Value

Plot

## See also

`ic_plot`

## Contents

Developed by Warren C. Jochem, Edson Utazi, Sahu Sujit.

Site built with pkgdown 1.6.1.
