# Supplementary material for: Bayesian Hierarchical Modeling Approaches for Combining Information From Multiple Data Sources to Produce Annual Estimates of National Immunization Coverage
Source: Biom J. 2026 Oct 6;68(5):e70189. doi: 10.1002/bimj.70189 (PMC13652340; doi:10.1002/bimj.70189)
Supplement: Supplementary file 2 [file BIMJ-68-e70189-s002.zip › code_and_data/imcover/imcover_0_2_5.zip!/imcover-main/docs/reference/predict.icfit.html]

Posterior predictions of vaccination coverage — predict.icfit • imcover


Toggle navigation


imcover
0.2.4

- Reference
- Articles
  - Model-based approaches to immunization coverage estimations in `R`: An introduction to the `imcover` package
- Changelog

# Posterior predictions of vaccination coverage

`predict.icfit.Rd`

Draw from the posterior predictive distribution.

```
# S3 method for icfit
predict(
  X,
  country = NULL,
  vaccine = NULL,
  t = 2,
  ratio_adj = TRUE,
  return_ic = TRUE
)
```

## Arguments

|  |  |
| --- | --- |
| X | object of type `icfit` or `iclist` |
| country | character vector of country codes to predict |
| vaccine | character vector of vaccine abbreviations to predict |
| t | Integer of the number of time steps ahead to predict. Default is 2. |
| ratio\_adj | Logical. Should multi-dose vaccines modelled by a ratio be back-transformed to coverage? Default is `TRUE`. |
| return\_ic | Logical. Should an `icfit` object be returned? |

## Value

If `return_ic` is `FALSE`, then a `data.frame` (or a
list of data frames) with posterior samples for predictions, labelled by
'country', 'vaccine', and 'time' columns. Returning an 'ic' object will
modify object `X` and add/update with a the 'prediction' element and
return the same type as `X`.

The default behaviour is to back-transform multi-dose vaccines which have
been converted to ratios into coverages. This is done by predicting the
ratio and multiplying the later dose ratio by the earlier dose's coverage.

## Contents

Developed by Warren C. Jochem, Edson Utazi, Sahu Sujit.

Site built with pkgdown 1.6.1.
