# Supplementary material for: Bayesian Hierarchical Modeling Approaches for Combining Information From Multiple Data Sources to Produce Annual Estimates of National Immunization Coverage
Source: Biom J. 2026 Oct 6;68(5):e70189. doi: 10.1002/bimj.70189 (PMC13652340; doi:10.1002/bimj.70189)
Supplement: Supplementary file 2 [file BIMJ-68-e70189-s002.zip › code_and_data/imcover/imcover_0_2_5.zip!/imcover-main/docs/reference/rbind.html]

Combine ic data objects by rows — rbind • imcover


Toggle navigation


imcover
0.2.4

- Reference
- Articles
  - Model-based approaches to immunization coverage estimations in `R`: An introduction to the `imcover` package
- Changelog

# Combine ic data objects by rows

`rbind.Rd`

Take a sequence of `ic.df` data-frame arguments and combine by rows.

```
# S3 method for ic.df
rbind(..., fill = TRUE, deparse.level = 1)
```

## Arguments

|  |  |
| --- | --- |
| ... | Objects to bind together. |
| fill | Should missing columns be added to data objects so that they can be combined? Default is `TRUE`. |
| deparse.level | See `rbind` Default is 1. |

## Value

An object of type `ic.df`.

## Details

The attributes of the ic data are used to match columns. Column
names are taken from the first object.

## Contents

Developed by Warren C. Jochem, Edson Utazi, Sahu Sujit.

Site built with pkgdown 1.6.1.
