# Supplementary material for: Bayesian Hierarchical Modeling Approaches for Combining Information From Multiple Data Sources to Produce Annual Estimates of National Immunization Coverage
Source: Biom J. 2026 Oct 6;68(5):e70189. doi: 10.1002/bimj.70189 (PMC13652340; doi:10.1002/bimj.70189)
Supplement: Supplementary file 2 [file BIMJ-68-e70189-s002.zip › code_and_data/imcover/imcover_0_2_5.zip!/imcover-main/docs/reference/single_lik_stan.html]

Multi-source immunisation coverage model with with Stan — single\_lik\_stan • imcover


Toggle navigation


imcover
0.2.4

- Reference
- Articles
  - Model-based approaches to immunization coverage estimations in `R`: An introduction to the `imcover` package
- Changelog

# Multi-source immunisation coverage model with with Stan

`single_lik_stan.Rd`

Sampling from a single likelihood model using a random effect
for data source.

```
single_lik_stan(X, verbose = TRUE, ...)
```

## Arguments

|  |  |
| --- | --- |
| X | Object of `ic.df` for analysis |
| verbose | Logical. Should messages be displayed? Default is `TRUE`. |
| ... | Arguments passed to `rstan::sampling` (e.g. iter, chains). |

## Value

An object of class `stanfit` returned by `rstan::sampling`

## Contents

Developed by Warren C. Jochem, Edson Utazi, Sahu Sujit.

Site built with pkgdown 1.6.1.
