# Supplementary material for: Bayesian Hierarchical Modeling Approaches for Combining Information From Multiple Data Sources to Produce Annual Estimates of National Immunization Coverage
Source: Biom J. 2026 Oct 6;68(5):e70189. doi: 10.1002/bimj.70189 (PMC13652340; doi:10.1002/bimj.70189)
Supplement: Supplementary file 2 [file BIMJ-68-e70189-s002.zip › code_and_data/imcover/imcover_0_2_5.zip!/imcover-main/docs/reference/summary.icfit.html]

Summary methods for 'ic' fit objects — summary.icfit • imcover


Toggle navigation


imcover
0.2.4

- Reference
- Articles
  - Model-based approaches to immunization coverage estimations in `R`: An introduction to the `imcover` package
- Changelog

# Summary methods for 'ic' fit objects

`summary.icfit.Rd`

Summaries of paramter estimates and MCMC convergence diagnostics (Monte Carlo
error, effective sample size, Rhat).

```
# S3 method for icfit
summary(X, pars = NULL, probs = c(0.1, 0.5, 0.9), ..., digits = 2)

# S3 method for iclist
summary(X, ...)

# S3 method for summary.icfit
print(X, digits = max(1, attr(X, "print.digits")), ...)

# S3 method for summary.iclist
print(X, digits = max(1, attr(X, "print.digits")), ...)
```

## Arguments

|  |  |
| --- | --- |
| X | An object of class `"summary.iclist"`. |
| pars | Optional character vector specifying a subset of parameters to display. |
| probs | Optional numeric vector of probability passed to `quantile`. |
| ... | Currently ignored. |
| digits | Number of digits to use for formatting numbers when printing. |

## Value

The `summary` method returns an object of class
`summary.icfit`, which is a matrix of summary statistics and
diagnostics, with attributes storing information for use by the
`print` method. The `print` method for `summary.icfit`
objects is called for its side effect and returns its input.

## Contents

Developed by Warren C. Jochem, Edson Utazi, Sahu Sujit.

Site built with pkgdown 1.6.1.
