# Supplementary material for: Bayesian Hierarchical Modeling Approaches for Combining Information From Multiple Data Sources to Produce Annual Estimates of National Immunization Coverage
Source: Biom J. 2026 Oct 6;68(5):e70189. doi: 10.1002/bimj.70189 (PMC13652340; doi:10.1002/bimj.70189)
Supplement: Supplementary file 2 [file BIMJ-68-e70189-s002.zip › code_and_data/imcover/imcover_0_2_5.zip!/imcover-main/docs/reference/survey_adjust.html]

Survey recall-bias adjustment — survey\_adjust • imcover


Toggle navigation


imcover
0.2.4

- Reference
- Articles
  - Model-based approaches to immunization coverage estimations in `R`: An introduction to the `imcover` package
- Changelog

# Survey recall-bias adjustment

`survey_adjust.Rd`

Apply a bias adjustment for selected vaccines in an immunisation coverage
survey dataset.

```
survey_adjust(X, adjVacc = c("DTP", "PCV"))
```

## Arguments

|  |  |
| --- | --- |
| X | Object of type `ic.df` |
| adjVacc | Character vector of vaccine abbreviations (omitting dose numbers) to adjust. |

## Value

An `ic.df` object with adjusted coverage estimates for selected
vaccines.

## See also

`ic_survey`

## Contents

Developed by Warren C. Jochem, Edson Utazi, Sahu Sujit.

Site built with pkgdown 1.6.1.
