# Supplementary material for: Bayesian Hierarchical Modeling Approaches for Combining Information From Multiple Data Sources to Produce Annual Estimates of National Immunization Coverage
Source: Biom J. 2026 Oct 6;68(5):e70189. doi: 10.1002/bimj.70189 (PMC13652340; doi:10.1002/bimj.70189)
Supplement: Supplementary file 2 [file BIMJ-68-e70189-s002.zip › code_and_data/imcover/imcover_0_2_5.zip!/imcover-main/docs/reference/survey_reduce.html]

Select survey records from an ic\_survey dataset — survey\_reduce • imcover


Toggle navigation


imcover
0.2.4

- Reference
- Articles
  - Model-based approaches to immunization coverage estimations in `R`: An introduction to the `imcover` package
- Changelog

# Select survey records from an `ic_survey` dataset

`survey_reduce.Rd`

Reduce immunisation survey coverage records based on set criteria of sample
size and/or priority types of reporting.

```
survey_reduce(X, minSample = 300, priority = c("card or history", "card"))
```

## Arguments

|  |  |
| --- | --- |
| X | Object of type `ic.df` created with `ic_survey`. |
| minSample | Minimum number of samples in the survey. |
| priority | Character vector of the priority of records when there are duplicates records for a given vaccine, survey, and year. Priority is established by the 'evidence' field of many household surveys. The highest priority record is given first in this argument. |

## Value

An `ic.df` object with possibly fewer records, based on the
selection criteria.

## See also

`ic_survey`

## Contents

Developed by Warren C. Jochem, Edson Utazi, Sahu Sujit.

Site built with pkgdown 1.6.1.
