# Supplementary material for: Bayesian Hierarchical Modeling Approaches for Combining Information From Multiple Data Sources to Produce Annual Estimates of National Immunization Coverage
Source: Biom J. 2026 Oct 6;68(5):e70189. doi: 10.1002/bimj.70189 (PMC13652340; doi:10.1002/bimj.70189)
Supplement: Supplementary file 2 [file BIMJ-68-e70189-s002.zip › code_and_data/imcover/imcover_0_2_5.zip!/imcover-main/docs/reference/waic.icfit.html]

Widely applicable information criterion (WAIC) — waic.icfit • imcover


Toggle navigation


imcover
0.2.4

- Reference
- Articles
  - Model-based approaches to immunization coverage estimations in `R`: An introduction to the `imcover` package
- Changelog

# Widely applicable information criterion (WAIC)

`waic.icfit.Rd`

Implementation of `waic()` methods from `loo` to compute WAIC from the
pointwise log-likelihood.

```
# S3 method for icfit
waic(X, ...)

# S3 method for iclist
waic(X, ...)
```

## Arguments

|  |  |
| --- | --- |
| X | Object of type `icfit` or `iclist`. |
| ... | Additional arguments. See `loo::waic()`. |

## Value

A named list (of class `c("waic", "loo")`) with components:

`estimates`
:   A matrix with two columns (`"Estimate"`, `"SE"`) and three
    rows (`"elpd_waic"`, `"p_waic"`, `"waic"`). This contains
    point estimates and standard errors of the expected log pointwise predictive
    density (`elpd_waic`), the effective number of parameters
    (`p_waic`) and the information criterion `waic` (which is just
    `-2 * elpd_waic`, i.e., converted to deviance scale).

`pointwise`
:   A matrix with three columns (and number of rows equal to the number of
    observations) containing the pointwise contributions of each of the above
    measures (`elpd_waic`, `p_waic`, `waic`).

## Details

For more details see loo. The developers of `stan`
recommend LOO-CV using PSIS (as implemented by the `loo::loo()`
function) because PSIS provides useful diagnostics as well as effective
sample size and Monte Carlo estimates.

## References

Watanabe, S. (2010). Asymptotic equivalence of Bayes cross validation and
widely application information criterion in singular learning theory.
*Journal of Machine Learning Research* **11**, 3571-3594.

## See also

`waic`

## Contents

Developed by Warren C. Jochem, Edson Utazi, Sahu Sujit.

Site built with pkgdown 1.6.1.
